# Antitubercular therapy for uveitis of undetermined cause with positive interferon-gamma release assay: a single-blind, single-centre, phase 2 randomised controlled trial

**DOI:** 10.1016/j.eclinm.2025.103511

**Published:** 2025-09-17

**Authors:** Rina La Distia Nora, Ikhwanuliman Putera, Mei Riasanti, Ratna Sitompul, Lukman Edwar, Made Susiyanti, Yulia Aziza, Muhammad Zakiy Waliyuddin, Erica Widodo, Ulifna Alfiya Sifyana, Priscilla Jessica, Rachel Ethelind, Gurmeet Singh, Willem A. Dik, Saskia M. Rombach, P. Martin van Hagen

**Affiliations:** a Department of Ophthalmology, Faculty of Medicine, Universitas Indonesia – Cipto Mangunkusumo Hospital, Jakarta, Indonesia; b Laboratory of Medical Immunology, Department of Immunology, Erasmus University Medical Centre, Rotterdam, the Netherlands; c Department of Ophthalmology, Erasmus University Medical Centre, Rotterdam, the Netherlands; d Department of Internal Medicine, Section Allergy & Clinical Immunology, Erasmus University Medical Centre, Rotterdam, the Netherlands; e Department of Internal Medicine, Respirology and Critical Illness Division, Faculty of Medicine, Universitas Indonesia, Cipto Mangunkusumo Hospital, Jakarta, Indonesia; f Department of Immunology, Faculty of Medicine, Chulalongkorn University, Bangkok, Thailand

**Keywords:** Antitubercular therapy, Corticosteroids, Interferon-gamma release assay, Steroid, Tuberculosis, Uveitis

## Abstract

**Background:**

No randomised controlled trial (RCT) has previously evaluated the effect of antitubercular therapy (ATT) in patients with uveitis of undetermined cause who tested positive on interferon-gamma release assays (IGRA), despite the absence of other identifiable causes of uveitis. We aimed to assess the efficacy and safety of treatment involving ATT compared to treatment without ATT in these patients, with respect to uveitis resolution and reduction in the risk of relapse.

**Methods:**

We conducted a single-blind, single-centre, phase 2 RCT at the uveitis clinic of Cipto Mangunkusumo Hospital in Jakarta, Indonesia, from August 16, 2021, to February 5, 2024. Seventy-six adults with newly diagnosed, active uveitis of undetermined cause and a positive IGRA were randomised 1:1 using block randomisation (block size 4) into two groups. Participants in the ATT group received an additional full course of ATT in addition to systemic corticosteroids. The control group received systemic corticosteroids without ATT. Investigators were masked to group assignment. The primary endpoint was the complete resolution of uveitis six months after randomisation. The trial is registered at ClinicalTrials.gov (NCT05005637).

**Findings:**

Seventy-six participants were randomly assigned to either ATT (n = 37) or control (n = 39) group. At primary end point, more participants assigned to the ATT group achieved the primary outcome of complete uveitis resolution compared to the control group (30/37, 81.1% vs. 20/39 participants; 51.3%, relative risk [RR] 1.58, 95% CI 1.12–2.23, *p* = 0.0060). Over the subsequent follow-up period, complete uveitis resolution was observed in 34 and 24 participants assigned to the ATT and the control groups, respectively. Additionally, uveitis relapse occurred in fewer participants assigned to the ATT group compared to those assigned to the control group (2/34 participants, 5.9% vs. 7/24 participants, 29.2%; HR 0.20, 95% CI 0.05–0.89, *p* = 0.0210). The findings regarding uveitis resolution and relapse rates were consistent in the per-protocol analysis.

**Interpretation:**

In IGRA-positive patients with uveitis of undetermined cause, initial treatment with ATT resulted in a significant benefit over those not receiving ATT.

**Funding:**

This work was supported by RISPRO-10.13039/501100014538LPDP (Riset Inovatif Produktif—Lembaga Pengelola Dana Pendidikan).

**Research in context**

**Evidence before this study**

We conducted a literature search in the electronic databases PubMed, EMBASE, and the Cochrane Library using the following keywords: “tubercular uveitis”, “uveitis OR intraocular inflammation”, “interferon-gamma release assay OR Mantoux OR tuberculin skin test”, “ocular tuberculosis”, and “treatment”, for articles published up to June 1, 2022. No randomised controlled trials were identified that evaluated the efficacy and safety of antitubercular therapy (ATT) in patients with uveitis of undetermined cause and positive TB immunoreactivity—patients who may represent a subset within the broader spectrum of ocular tuberculosis (TB) or TB-associated uveitis. Interferon-gamma release assays (IGRA), such as QuantiFERON-TB Gold Plus (QFT), can identify individuals with TB infection, but its positivity does not necessarily indicate active disease. It is a dilemma to commence a full course of ATT in patients with uveitis of undetermined cause and IGRA positivity, as there is currently no prospective randomised study assessing the efficacy and safety of ATT in this patient.

**Added value of this study**

This is the first phase 2 randomised controlled trial to assess the impact of ATT in comparison to no ATT (e.g., systemic corticosteroids alone) in patients with uveitis of undetermined cause and IGRA positivity. Our findings demonstrate a significant improvement in complete uveitis resolution and a reduction in relapse rates who received ATT. While adverse effects were observed, the benefit of ATT was considered to outweigh its risks.

**Implications of all the available evidence**

In TB-endemic settings, these results support considering ATT as part of treatment strategy for uveitis of undetermined cause in patients with positive TB immunoreactivity test. With appropriate monitoring for adverse events, this approach could improve outcome and reduce the risk of uveitis relapse. The preliminary data from this study could inform future, larger-scale studies and guide treatment decisions.

## Introduction

Uveitis is an umbrella term for intraocular inflammation caused by infectious or non-infectious causes.^1^^,^^2^ Currently, more than 30 diseases associated with uveitis have been identified.^1^^,^^2^ However, more than one-third of clinical uveitis cases lack definitive aetiologies, falling under the category of “uveitis of undetermined cause” or idiopathic.^2^^,^^3^ In clinical practice, uveitis is considered infectious until proven otherwise.^4^^,^^5^ Therefore, systematic investigations using various diagnostic tests, along with multidisciplinary consultations are often required.^6^^,^^7^ In cases of non-infectious uveitis, immunosuppressants—starting with systemic corticosteroids administered locally, systemically, or both—are the mainstay of treatment.^5^ For infectious uveitis, appropriate antimicrobial treatment is essential for achieving uveitis resolution^2^; otherwise, the condition maintains and may worsens even into blindness such as in tubercular uveitis.^8^

Among the infectious causes of uveitis, tuberculosis (TB) is an aetiology that must always be considered. According to the 2024 report by the World Health Organization (WHO), the incidence of TB in Indonesia was 387 cases per 100,000 population.^9^ Consequently, Indonesia is classified as a high TB burden country and ranks as the second highest globally.^9^ In countries with a high incidence of TB, such as Indonesia, uveitis associated with concurrent active systemic TB accounts for 8% of newly referred uveitis cases in our setting.^10^ Interestingly, among patients with uveitis of undetermined cause, some exhibited TB immunoreactivity as detectable through interferon-gamma release assays (IGRA) or tuberculin skin tests (TST). Up to 40% of all referred uveitis patients at our centre were classified as having uveitis of undetermined cause with a positive IGRA.^10^ The clinical characteristics of these patients revealed that the majority did not present with the classical manifestations typically associated with TB-related uveitis.^11^ Importantly, more than one-third of the affected eyes were already categorised as “blind” at the time of initial presentation to our centre.^12^ In the absence of confirmatory evidence of active TB, these patients were often treated empirically for *presumed* TB,^12^ underscoring both the diagnostic uncertainty and the serious risk of irreversible vision loss. Moreover, the proportion of such patients slightly exceeded the rate of positive TB immunoreactivity in the general population in Indonesia (approximately 35% of the general population),^10^^,^^13^ raising concerns that the presenting uveitis may represent a true manifestation of extrapulmonary TB in the eye.

Before the current trial, multiple retrospective studies reported treatment outcomes in the management of TB uveitis, but no prospective trials had been conducted.^14^ There is no gold standard for diagnosing TB uveitis,^15^^,^^16^ partly due to the suboptimal diagnostic performance of current ocular fluid polymerase chain reaction (PCR) tests for detecting *Mycobacterium tuberculosis* (*Mtb*) genomes in ocular fluid samples.^7^^,^^17^ Most studies evaluating treatment outcome included both uveitis patients with proven active systemic TB and those showing TB immunoreactivity after other causes had been excluded. As previously summarised, treatment with antitubercular therapy (ATT) in these patients demonstrated an overall results toward a better resolution of uveitis but failed to achieve statistical significance.^14^^,^^18^ Cautious interpretation is warranted, as our previous retrospective analysis suggests that the severity of presenting uveitis might influence treatment decisions regarding the initiation of ATT, complicating the interpretation of the actual benefits of ATT in this context.^19^ Of note, based on the opinions of uveitis experts,^16^ in the absence of active systemic TB, there is no strong consensus on initiating ATT unless ophthalmologic presentations such as choroidal granuloma or serpiginous-like choroiditis (SLC) are observed. Other clinical presentations, including chronic anterior uveitis or panuveitis, were considered potential indications for ATT in high TB-endemic regions; however, additional supporting evidence, such as signs of healed or old TB lesions on chest radiography, in combination with a positive TB immunoreactivity test, is necessary for treatment initiation. In light of this, we conducted a phase 2 randomised controlled trial to determine whether ATT would eventually improve outcomes compared to treatment without ATT in terms of uveitis resolution and relapse, during subsequent follow-up of patients with uveitis of undetermined cause who were showing TB immunoreactivity, based on IGRA test positivity.

## Methods

### Study design and participants

This single-blinded, phase 2 randomised controlled trial was conducted at Cipto Mangunkusumo Hospital, a tertiary eye hospital in Jakarta, Indonesia. This trial was conducted to assess whether a full course of ATT would increase the proportion of complete uveitis resolution, as well as reduction of uveitis relapse compared to a non-ATT treatment approach in patients with uveitis of undetermined cause who were IGRA positive. This trial adhered to the CONSORT 2025 reporting guidelines^20^ (see appendix).

All newly referred uveitis patients were evaluated for eligibility at the outpatient uveitis clinic. Inclusion criteria were: (1) adults (age ≥18 years) with newly diagnosed, clinically active uveitis of undetermined cause, following standardised workup at our centre (see Appendix); (2) positive QuantiFERON TB-Gold Plus (QFT) test result (>0.35 U/mL; Kimia Farma Laboratory, Depok, PT. Kimia Farma Diagnostika); (3) local residence in Jakarta, Bogor, Depok, Tangerang, or Bekasi; and (4) consent to participate in the study through the entire monitoring period. Exclusion criteria were: (1) confirmed active systemic TB or living with an active TB patient; (2) HIV-positive status; (3) visual acuity less than hand movement with signs of phthisis on ophthalmological examinations and ultrasound; (4) impaired liver function or other systemic conditions, as assessed by the internist-pulmonologist, that precluded eligibility for ATT; (5) pregnancy, (6) recent antibiotic use, including ATT, or systemic corticosteroid use exceeding 8 mg prednisone equivalent daily within the last 14 days, (7) cases where ocular fluid analysis was performed and yielded positive results for pathogens (including *Mtb*, which is part of our standard panel), and (8) clinical presentation of choroidal granuloma or TB-SLC.

Eligible participants underwent standardised workup and received consultations from the participating internist-pulmonologist at our hospital. The internist-pulmonologist was unaware of the randomisation and provided all patients with the necessary information and counselling regarding ATT prescription and initiation. Following the initial consultation, a research manager who had the first access to the ATT collection informed participants whether ATT would be initiated, in accordance with the randomisation procedure. Routine follow-ups were scheduled for week 2, month 1, and every month until the six-month follow-up at the outpatient uveitis clinic. Dropout criteria included participants who became pregnant or experienced severe systemic adverse events during the study and subsequently chose to discontinue participation; such individuals were withdrawn from the trial.

### Ethics

Ethical approval from the local ethics committee was granted on June 21, 2021 (the Ethics Committee of the Faculty of Medicine, Universitas Indonesia, Jakarta, Indonesia; reference number: KET-616/UN2.F1/ETIK/PPM.00.02/2021, version: 0.3 and ND-373/UN2.F1/ETIK/PPM.00.02/2022), and legal permission from the study site (Cipto Mangunkusumo Hospital; reference number: LB.02.01/2.6.1/0702/2021 and LB.02.03/2.6.1/0699/2022) to conduct the RCT was obtained on July 14, 2021. The trial was registered at ClinicalTrials.gov (NCT05005637) on August 10, 2021. The actual study start date was July 27, 2021, and the first participant was enrolled on August 16, 2021. The time between the actual study start date and the enrolment of the first participant was used to collaborate with our multidisciplinary team (logistics, hospital admission, pharmacy, internal medicine department, diagnostic labs, arrangement of in-house QFT tests, etc.), as participants needed to undergo screenings and multiple visits to different departments before their eligibility could be determined. We consider these activities as part of the study, as the hospital regards them as research-related due to certain adjustments made to routine clinical care. The participant enrolment period spanned from August 16, 2021, to February 5, 2024, with the last follow-up for secondary outcome measurement occurring on November 30, 2024. The extended recruitment period was due to the post-COVID recovery of the healthcare system and the inclusion criteria requiring participants’ commitment for regular follow-up. As the trial site is a tertiary referral hospital in Indonesia, we recruited only patients living nearby or those able to travel regularly, ensuring consistent follow-up.

### Randomisation and masking

After eligibility assessment, participants were randomly assigned in a 1:1 ratio to one of the two treatment groups without stratification factors. Randomisation was conducted by a dedicated research manager using block randomisation (block size of 4). Investigators, including clinical graders, were masked to the treatment allocation of participants throughout the study. However, masking of treatment allocation from patients was not feasible due to differences in dosing, regimen, and treatment duration between the investigational products.

### Procedures

The research manager initially received the ATT and provided it to participants assigned to the ATT group, while those in the control group were withheld from ATT drugs. All included participants received corticosteroids or anti-inflammatory drugs at the discretion of the attending uveitis specialists. Generally, topical corticosteroids were prescribed when significant anterior chamber and anterior vitreous inflammation were observed. Local treatments, such as parabulbar injections of triamcinolone acetate, were allowed if deemed necessary by the attending uveitis specialists, who were unaware of the randomisation. There were no restrictions on topical or local treatments throughout the entire follow-up period, including surgical interventions, if indicated.

A two-independent proportion sample size calculation was conducted. Based on the Multicenter Uveitis Steroid Treatment (MUST) Trial in non-infectious uveitis, the proportion of uveitis patients with active disease at six months following treatment was found to be 44%.^21^ We assumed a 30% difference in uveitis resolution between the two groups. Using a Zα of 1.96 and a Zβ of 0.84 (for a two-sided significance level of 5% and 80% power), the estimated sample size required for each treatment group was 35 participants. We increased this calculated sample size by 10%, resulting in a total of 39 participants in each treatment group.

#### ATT group

The full course of ATT comprised two months in the intensive phase, followed by seven months in the continuation phase. During the intensive phase, a fixed-dose combination (FDC) was administered, with each tablet containing 150 mg of rifampicin, 75 mg of isoniazid, 400 mg of pyrazinamide, and 275 mg of ethambutol. The continuation phase used an FDC that included rifampicin and isoniazid. The ATT dosage was determined based on body weight: individuals weighing 30–37 kg received 2 tablets; those weighing 38–54 kg received 3 tablets; individuals weighing 55–70 kg received 4 tablets; and those over 70 kg received 5 tablets. During the intensive phase, ATT was administered once daily, while in the continuation phase, it was given three times per week. Participant monitoring, including assessments of adverse events and laboratory parameters, was conducted by the Department of Internal Medicine.

In addition to ATT, selected participants were prescribed local or systemic corticosteroids, including oral methylprednisolone, starting at a dosage of 0.8 mg/kg of body weight per day and tapered gradually (e.g., every three days or weekly) based on the severity of intraocular inflammation observed during presentation and follow-up visits. The decision to initiate combination treatment or to add systemic corticosteroids during follow-up was made by the uveitis specialist, who was unaware whether the patients were in the ATT or control group. The dosing of systemic corticosteroids was not adjusted when combined with ATT and was therefore similar to that of the control group (see below).

#### Control group

The treatment for the control group primarily involved systemic corticosteroids without ATT. Local corticosteroids were prescribed according to the standard care by the attending uveitis specialists and tailored to the severity of the intraocular inflammation. Additionally, all participants in this group received oral methylprednisolone at a dosage of 0.8 mg/kg of body weight per day (maximum of 56 mg/day), which was tapered gradually based on the intraocular inflammation observed. Tapering of oral methylprednisolone involved reducing the dose by 8 mg for doses above 20 mg and by 4 mg for doses below 20 mg. All patients receiving systemic corticosteroids, including those in the ATT group, were also prescribed vitamin D, calcium supplements, and gastric protectors (e.g., omeprazole or antacids). Treatment switching could be requested by study participants at their discretion and was to be communicated to the research manager, upon which the treatment regimen was adjusted accordingly.

### Outcomes

Participants underwent clinical assessments at baseline, week 2, month 1, and monthly until month 6. The research manager maintained close contact with participants through phone calls to remind them of their scheduled visits. Subsequent follow-up reminders after six months were provided to participants by the research manager, although these extended visits were not mandatory. Clinical assessments included standard ophthalmological evaluations using slit-lamp biomicroscopy to quantify intraocular inflammation and any relevant pathology. Ocular imaging modalities, such as optical coherence tomography (OCT), fluorescein angiography, or ultrasound, could be performed in addition to the standard ophthalmological examination if necessary. To maintain masking, participants were instructed not to disclose their ATT intake to the attending uveitis specialists assigned as clinical graders. At each visit, the research manager accompanied the patient to ensure trial integrity. The uveitis specialist graded the inflammation and adjusted the dose of corticosteroids accordingly. The team of uveitis specialists consisted of five individuals, and each patient was not restricted to follow-up with a single designated uveitis specialist (the preliminary agreement on disease activity based on anterior chamber cells showed a kappa value of 0.64).

The primary outcome measure was the complete resolution of uveitis at the patient level (both eyes) at six months. Criteria for complete uveitis resolution applied to both eyes, in cases with bilateral uveitis, included: (1) fewer than or equal to 0.5+ anterior chamber cells according to the Standardised Uveitis Nomenclature (SUN) grading system, fewer than or equal to 0.5+ vitreous haze based on clinical grading using the NEI (National Eye Institute) scale, and no active retinal or choroidal lesions (new increases in size and number, as well as changes in margins, as assessed by the attending uveitis specialist); and (2) no more than 7.5 mg of oral prednisone daily (i.e., methylprednisolone at 6 mg or less daily) and fewer than or equal to 2 drops of prednisolone acetate 1% (or equivalent) per day. These were in line with the previous criteria to define treatment response in uveitis.^22^ These criteria had to be maintained for at least 90 days following the visit when the first complete uveitis resolution was recorded. If there was insufficient follow-up after resolution, maintained uveitis resolution was still considered, as patients were contacted via telephonic consultation to confirm the absence of persistent symptoms or new complaints during the missed visits. Patients who achieved uveitis inactivity at any time within the 6-month period were considered resolved, provided that no disease activity occurred during the subsequent 90 days from the start of inactivity and all other criteria were met. Thus, resolution at 6 months does not necessarily require inactivity to have begun before month 3. Participants who met criterion 1 but not criterion 2 were classified as having partial uveitis resolution, and for the primary endpoint classification, they were considered to have non-complete uveitis resolution. Secondary outcomes included the details of treatment failure (partial resolution or non-responsive), time to uveitis resolution, uveitis relapse following complete resolution, and eye-level outcomes, which consisted of the differences in visual acuity between baseline and month 6, as well as the incidence of secondary glaucoma (e.g., occurrences of increased intraocular pressure (IOP) above 21 mmHg in at least two consecutive visits that necessitated prescription of IOP-lowering medications). Visual acuity was considered to increase if the difference in best-corrected visual acuity between month 6 and baseline visits (measured with Snellen, in decimal equivalent) was equal to or greater than 0.1, stable if within the range of −0.1 to 0.1, and decreased if less than −0.1. If any cataract or vitreoretinal surgery was performed before the six-month follow-up, the last recorded visual acuity prior to the surgery was used. Uveitis relapse was defined as any worsening of ocular inflammation (including a two-step increase in anterior chamber or vitreous cells as per the SUN grading system) or the occurrence of clinically new inflammatory activity (such as choroidal or retinal lesions) that necessitated a modification of local or systemic uveitis treatment after a minimum of 90 days of complete uveitis resolution at the patient level.^23^^,^^24^ As per its definition, time to relapse was calculated from the first visit showing inactive uveitis (uveitis resolution, rather than the six-month time point) until the first notification of uveitis relapse. Any symptoms reported during telephonic follow-up were not directly counted as relapse events, as participants were required to attend a clinic visit for relapse to be formally defined according to the study criteria. Otherwise, such cases were right-censored accordingly.

### Statistical analysis

We developed a statistical analysis plan before the final analysis (see details in Appendix). No interim analysis was conducted. All analyses were first conducted using the intention-to-treat (ITT) approach. The ITT population included all participants who underwent randomisation, regardless of any treatment switching that occurred. At the six-month visit (±28 days), outcome data were missing for 1 participant in the ATT group and 7 participants in the control group. These missing values were addressed using last observation carried forward (LOCF) imputation for the primary analysis, as we relied on objective clinical assessment during clinic visits. Telephonic follow-up, as described in the previous section, was conducted to assess the general condition and explore reasons for missed visits. Based on these interviews, we determined that LOCF remained appropriate as the primary analytical approach, as most participants did not report meaningful changes in symptoms compared to their last clinic visit; in 6 of 8 participants with missing six-month data, the most recent visit had occurred at month four or later. Nevertheless, as a sensitivity analysis, multiple imputation (MI) was conducted under the missing-at-random (MAR) assumption. Ten imputed datasets were generated using fully conditional specification, with treatment group and baseline clinical characteristics included in the imputation model. Logistic regression was then used to analyse the pooled dataset, accounting for the imputed values. To further supplement this analysis, per-protocol analysis was also performed. The per-protocol population included all participants who were randomised, excluding those who deviated from the treatment protocols. This included individuals who received treatment modifications due to adverse events, non-compliance, or treatment crossover (these participants were completely excluded from the per-protocol analysis). Missing primary outcome data were handled using the LOCF method in the per-protocol analysis. Adherence to treatment was assessed by the internist-immunologist through logbook documentation and tablet count review. We also performed an analysis accounting for treatment regimen switches, presenting this as an analysis by last treatment received. This analysis was conducted only for the uveitis relapse outcome following resolution. The results of the two analyses, with and without adjustment for switching (analysis by last treatment received), were reported. However, we consider the ITT analysis (without adjustment for switching) to be more appropriate to evaluate the primary outcome, given that the purpose of the study was to compare the two strategies according to the treatment received at the time of randomisation. For the analysis of the uveitis relapse outcome, we also stratified participants in the ATT group based on whether they received ATT in combination with systemic corticosteroids or without it, resulting in three treatment groups. Analysis by last treatment received was performed with recalculation of the six-month follow-up time based on the actual treatment received, beginning from the start of their actual treatment.

Baseline characteristics were summarised according to trial group for the ITT populations. The primary outcome analysis focused on the proportion of complete uveitis resolution and the corresponding relative risk between the ATT and control groups. Cox proportional-hazards models along with Kaplan–Meier estimates were used to estimate any uveitis resolution (partial or complete, whenever the first was recorded) between the ATT and control groups. For uveitis relapse outcomes, we first identified all participants who achieved complete uveitis resolution, regardless of the follow-up time. The Cox regression analysis used individual right censoring, and no data imputation, either LOCF or multiple imputation, was used to estimate the mean time to uveitis resolution. The number of participants experiencing any adverse events was also reported. A *p*-value of less than 0.05 was considered statistically significant. All statistical analyses were performed using IBM SPSS Statistics version 28.0.1.0 (142) for Windows (SPSS Inc.).

### Role of the funding source

The funders of the study had no role in study design, data collection, data analysis, data interpretation, or writing of the report.

## Results

**Fig. 1:**
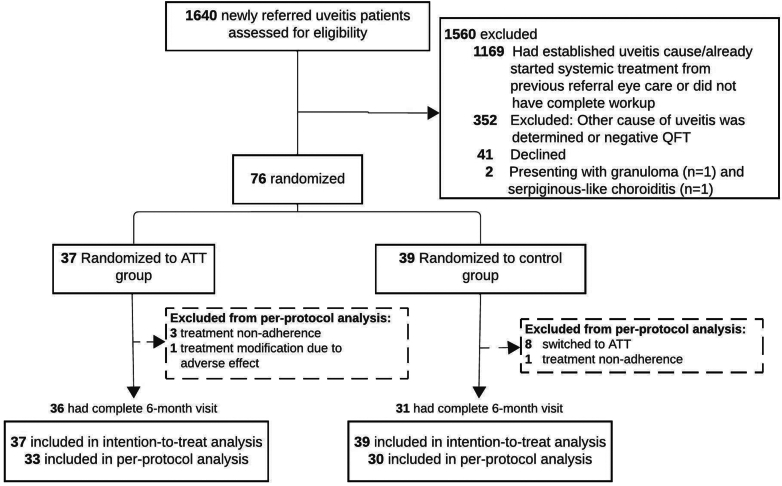
Screening, randomisation, and analysis. Among patients initially receiving steroids alone, eight were later switched to ATT. All randomised patients were included in the primary ITT analysis. Per-protocol analysis was conducted based on the actual treatment received, excluding participants with protocol deviations (treatment switching, deviation, and non-adherence). Participants with missing 6-month visits were included in the outcome analysis for both the intention-to-treat and per-protocol analyses, with missing data handled as described in the Methods section.

A total of 76 participants provided written informed consent and were randomised, with 37 assigned to the ATT and 39 to the control groups. In the ATT group, four participants from the ITT population were excluded from the per-protocol analysis: three due to non-adherence to the ATT regimen (defined as missing more than two weeks of prescribed drugs) and one whose ATT regimen was adjusted due to severe adverse events. In the control group, nine participants were excluded from the per-protocol analysis: eight who opted to receive ATT instead of continuing with systemic corticosteroids only, and one who was non-adherent to the corticosteroid treatment regimen. As a result, 33 participants in the ATT group and 30 in the control group were included in the per-protocol analysis (Fig. 1). No study participants discontinued the trial (dropped out), including through withdrawal.

Baseline characteristics in the ITT population were balanced across groups (Table 1). In both groups, uveitis symptoms in nearly half of the participants had lasted for less than three months. We calculated the cumulative systemic corticosteroid intake (prednisone equivalent; cortiser.ser.es/) during the six-month observation period. The ATT group had a lower dosage compared to the control group (median: 0 mg, Q1–Q3: 0–1170 mg vs. 3275 mg, Q1–Q3: 2445–3960 mg; *p* < 0.0001). Eleven participants in the ATT group (11/37; 29.7%) received systemic corticosteroids within the first six months following randomisation. Local steroid injections (i.e., sub-Tenon's triamcinolone acetonide) were administered once in two patients in the ATT group and one patient in the control group. In terms of topical steroid use, prednisolone acetate 1% eye drops were prescribed to 26 participants (70.3%) in the ATT group and 33 participants (84.6%) in the control group, initially at 4–6 drops per day. No intravitreal corticosteroid implant or injection was performed.

**Table 1:** Baseline characteristics of patients in the intention-to-treat population.

	ATT group (N = 37)	Control group (N = 39)
**Patient-level characteristics**		
Age, in years (SD)	42 (13)	40 (12)
Sex		
Male	12 (32%)	8 (20%)
Female	25 (68%)	31 (80%)
Duration of uveitis symptoms		
<3 months	15 (41%)	17 (44%)
3–12 months	12 (32%)	15 (38%)
>12 months	10 (27%)	7 (18%)
Diabetes mellitus type 2	5 (14%)	3 (8%)
Previous TB diagnosis or treatment	0 (0%)	0 (0%)
Erythrocyte sedimentation rate (mm/hour)	27 (10–37)	21 (13–29)
Leucocyte count (/mm)	8820 (7220–9820)	7860 (6660–9718)
Chest radiology		
Healed tuberculosis lesions (e.g., fibrotic changes)	4 (11%)	2 (5%)
No remarkable abnormality to tuberculosis infection	33 (89%)	37 (95%)
**Ocular characteristics**	**ATT group (N eyes = 55)**	**Control group (N eyes = 60)**

Visual acuity (Snellen)		
≥6/18	20 (36%)	26 (43%)
6/60–6/18	8 (14%)	14 (23%)
3/60–<6/60	3 (6%)	6 (10%)
<3/60	24 (44%)	14 (23%)
Keratic precipitates		
Mutton-fat	4 (7%)	5 (8%)
Other types	16 (29%)	28 (47%)
No keratic precipitates	35 (64%)	27 (45%)
Any corneal lesions (e.g., infiltrate, cicatrices)	7 (13%)	2 (3%)
Iris nodules	1 (2%)	2 (3%)
Anterior chamber cells grade ≥2+ (SUN)	15 (27%)	16 (27%)
Posterior synechiae	42 (76%)	37 (62%)
Broad-based synachiae	7/42 (17%)	4/37 (11%)
Vitritis	26 (45%)	30 (50%)
Retinal vasculitis (any type)	4 (7%)	10 (17%)
Specific lesions		
Multifocal choroiditis	0 (0%)	0 (0%)
Other choroidal/retinal lesions^a^	4 (7%)	6 (10%)
Non-glaucomatous optic nerve involvement (e.g., edematous)	8 (14%)	13 (22%)
Uveitic macular edema	8 (14%)	11 (18%)
Retinal detachment (any type)	4 (7%)	1 (2%)
Anatomical subtype of uveitis		
Keratouveitis	4 (7%)	2 (3%)
Anterior uveitis	6 (11%)	11 (18%)
Intermediate uveitis	3 (6%)	5 (8%)
Anterior-intermediate uveitis	1 (2%)	4 (7%)
Posterior uveitis	9 (16%)	9 (15%)
Panuveitis	31 (56%)	27 (45%)
Scleritis/sclerouveitis	1 (2%)	2 (3%)

SD, standard deviations.

a All choroidal retinal lesions/infiltrates/changes (old or new) excluding serpiginous, granulomatous, or typical multifocal lesions visible on slit-lamp biomicroscopy.

By six months, 30 out of 37 participants (81.1%) in the ATT group achieved complete uveitis resolution compared to 20 out of 39 participants (51.3%) in the control group (relative risk [RR], 1.58; 95% CI, 1.12–2.23; *p* = 0.0060). The values represent the observed outcomes following LOCF. Sensitivity analysis using MI method produced consistent results, with the ATT group showing higher odds of resolution (odds ratio [OR], 3.15; 95% CI, 1.11–8.93; p = 0.0310). An additional sensitivity analysis including only participants with non-remarkable chest radiographic findings (i.e., no evidence suggestive of TB infection) also demonstrated a consistently higher likelihood of uveitis resolution in the ATT group (26/33, 78.8%) compared to the control group (19/37, 51.4%; RR, 1.53; 95% CI, 1.07–2.20; p = 0.0170). Per-protocol analysis showed a consistent finding (RR, 1.53; 95% CI, 1.06–2.22; *p* = 0.0150, Table 2).

**Table 2:** Analysis of primary and secondary outcomes.

	ATT group	Control group	Relative risk or Hazard ratio (95% CI)	*p*
**Primary outcomes**				
As intention-to-treat population				
N	37	39		
Complete uveitis resolution	30 (81.1%)	20 (51.3%)	1.58 (1.12–2.23)	0.0060
As per-protocol population				
N	33	30		
Complete uveitis resolution	27 (81.8%)	16 (53.3%)	1.53 (1.06–2.22)	0.0150
**Secondary outcomes (intention-to-treat analysis)**				
Details of uveitis resolution				
Complete	30 (81.1%)	20 (51.3%)	–	0.0040
Partial	0 (0%)	8 (20.5%)	–	
Non-responsive	7 (18.9%)	11 (28.2%)	–	
Estimated mean time-to-any resolution (in months)	3.6 (2.1–5.7)	3.0 (1.8–4.2)	0.96 (0.59–1.58)	0.8888
Incidence of uveitis relapse	2/34 (5.9%)	7/24 (29.2%)	0.20 (0.05–0.89)	0.0210
Estimated mean time-to-uveitis relapse (in months)	17.7 (16.1–19.4)	14.3 (9.2–19.4)	0.14 (0.03–0.69)	0.0160
Ocular outcomes				
Visual acuity changes				
Increase	8 (14.5%)	11 (18.3%)	–	0.7550
Stable	33 (60.0%)	32 (53.3%)	–	
Decrease	14 (25.5%)	17 (28.3%)	–	
Secondary glaucoma or increased intraocular pressure	18 (32.7%)	18 (30.0%)	1.05 (0.61–1.81)	0.7530

The last observation carried forward (LOCF) method was used to handle missing outcomes in both the intention-to-treat (ITT) and per-protocol analyses.

Mean time-to-any resolution was comparable between groups, estimated at 3.6 months for the ATT group and 3.0 months for the control group (hazard ratio [HR], 0.96; 95% CI, 0.59–1.58; *p* = 0.8888) (Table 2). In the ATT group, no participant had partial uveitis resolution, while 8 (20.5%) in the control group achieved partial resolution, as they still required corticosteroids to maintain uveitis control. The ATT group also had fewer cases of non-responsive uveitis than the control group (18.9% vs. 28.2%, *p* = 0.0040).

**Fig. 2:**
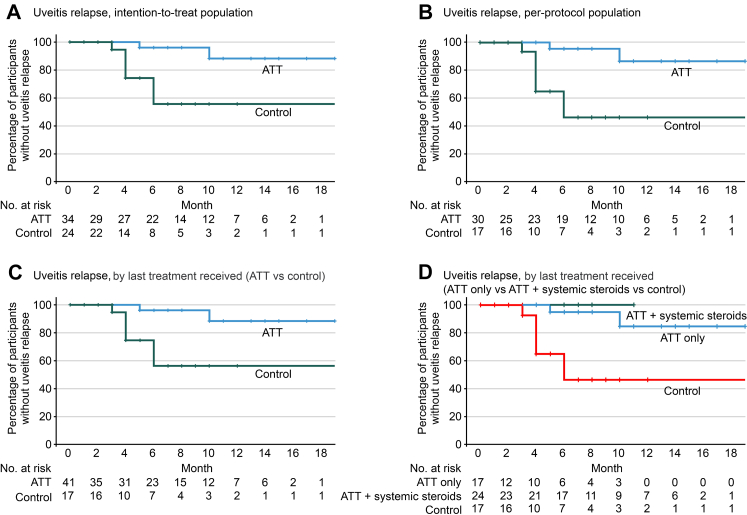
Uveitis relapse after achieving complete uveitis resolution. Panel A shows uveitis relapse in intention-to-treat population. Panel B shows uveitis relapse in per-protocol population. Panel C shows uveitis relapse by last treatment received (antitubercular therapy (ATT) vs steroids). Panel D shows uveitis relapse by last treatment received with three arms. The ATT group is further categorised into those who received a combination of systemic steroids along with the prescribed ATT and those who received ATT only. For panel C and D, each participant is represented only once in the analysis, according to the final treatment allocation they actually received. Month in the x axis was calculated from the time complete uveitis resolution was achieved.

Participants were encouraged to continue follow-up beyond the six-month endpoint. The duration of follow-up from randomisation to the last clinic visit was comparable between the ATT and control groups (mean follow-up duration: 13.8 months for the ATT group vs. 10.2 months for the control group, *p* = 0.1130). Among all participants who achieved complete uveitis resolution at any point during follow-up, including those with resolution beyond the six-month visit (34 in the ATT group and 24 in the control group), relapse rates were lower in the ATT group than in the control group. Specifically, uveitis relapse occurred in 2 of 34 participants (5.9%) in the ATT group, compared to 7 of 24 participants (29.2%) in the control group (*p* = 0.0210). The estimated mean time to uveitis relapse was 17.7 months in the ATT group and 14.3 months in the control group. These estimates reflect the area under the survival curve and are subject to right-censoring (HR, 0.14; 95% CI, 0.03–0.69; *p* = 0.0160) (Fig. 2A). Additional analysis conducted in the per-protocol (HR with ATT, 0.13; 95% CI, 0.03–0.65; *p* = 0.0130, Fig. 2B) also showed a significant reduction in uveitis relapse with ATT treatment.

Treatment switching was observed in this trial (Fig. 1). The eight participants (8/39, 20.5%) in the control group who switched to the ATT group all had active uveitis at the time of switching. Further stratification of the ATT group into participants receiving ATT with and without systemic corticosteroids according to the last treatment received also showed a consistent findings in terms of uveitis relapse following complete uveitis resolution (see Fig. 2C and D). For ocular outcomes, comparable changes in visual acuity and incidence of secondary glaucoma were observed between the ATT and control groups.

Adverse events were reported in 13 participants (35.1%) in the ATT group and 5 participants (12.8%) in the control group. Drug-induced liver injury (DILI) was observed in 1 participant in the ATT group. In this participant, close monitoring was undertaken. ATT was discontinued and subsequently reinitiated without pyrazinamide under the supervision of the internist-pulmonologist. Additional adverse events included dyspepsia, elevated uric acid levels, and hypersensitivity reactions, were all more frequent in the ATT group (Table 3). Four participants in the ATT group who experienced dyspepsia were managed effectively with antacids or proton-pump inhibitors, while the remaining cases were self-managed without additional medication. All cases of hyperuricemia were asymptomatic. Uric acid-lowering therapy (i.e., allopurinol) was prescribed for two participants. In patients who developed intolerable side effects due to the use of systemic corticosteroids, these drugs were discontinued, and methotrexate was initiated as a second-line immunosuppressant. Skin hypersensitivity reactions were managed with antihistamine medication. No serious opportunistic infections or new active TB cases were identified among participants receiving systemic corticosteroids alone during the study period.

**Table 3:** Adverse events according to the intention-to-treat population.

	ATT group (N = 37)		Control group (N = 39)	
	N events	Percentage (%)	N events	Percentage (%)
Drug-induced liver injury (DILI)	1	2.7%	0	0
Dyspepsia or gastritis	7	18.9%	1	2.6%
Rectal bleeding	0	0	1	2.6%
Chronic fatigue	0	0	1	2.6%
Intolerable Cushing's syndrome	0	0%	1	2.6%
Skin lesions/hypersensitivity reaction	2	5.4%	1	2.6%
Elevated uric acid level	3	8.1%	0	0
Total	13	35.1%	5	12.8%

## Discussion

This phase 2 randomised controlled trial provides the first evidence that a treatment approach consisting of ATT was more beneficial than systemic corticosteroids alone for patients with uveitis of undetermined cause who were IGRA-positive. In the extended follow-up period beyond six months, the rate of recurrence or relapse of the uveitis was less in the ATT treatment arm. However, careful monitoring for adverse events is essential in patients receiving ATT, as well as those receiving systemic corticosteroids. Based on our findings, we are of the opinion that the benefits of ATT outweigh the risks.

Treating patients with uveitis of undetermined cause with positive for TB immunoreactivity and no clinical signs of active TB remains challenging.^25^ Clinicians are generally reluctant to start ATT on positive TB testing alone,^16^^,^^25^ in accordance to the current recommendations.^26^, ^27^, ^28^ This dilemma is heightened when uveitis is non-responsive to corticosteroids or when biologics such as anti-TNF therapy are considered.^29^ However, initiating anti-TNF treatment in recalcitrant cases of uveitis of undetermined cause with a positive IGRA is problematic if ATT has not been initiated first, as the risk of active TB disease is significantly increased.^30^ Hence, our study provides evidence that initial treatment incorporating ATT yields more favourable outcomes in this patient spectrum, particularly in high TB-burden settings. The recent European Alliance of Associations for Rheumatology (EULAR) recommendation mandates TB screening in autoimmune inflammatory rheumatic diseases before initiating immunosuppressants (including glucocorticoids). ATT, whether as prophylaxis or a full course regimen, is recommended.^31^ Although no TB reactivation was observed during follow-up, full course of ATT herein demonstrated benefits in uveitis control. It is worth mentioning that, although some participants in the ATT group received a combination with systemic corticosteroids, no corticosteroid dosage adjustment was made. Rifampicin is known to reduce the bioavailability of glucocorticoids,^32^ and therefore, the uveitis resolution observed in the ATT group is perceived to be mainly attributed to the effect of ATT.

While ATT showed superior outcomes in our trial, a cautious interpretation is still warranted. The current study was conducted at a single centre in Indonesia, where TB prevalence is high. In high TB-burden settings, the likelihood of uveitis being a true manifestation of extrapulmonary TB is greater than in low-burden settings.^33^ It is also important to note that the effectiveness of the standard ATT regimen observed in this trial may, in part, be attributed to the relatively low proportion (approximately 2–3%) of newly diagnosed patients with bacteriologically confirmed pulmonary TB in our setting who have multidrug-resistant TB (MDR-TB) or rifampicin-resistant TB (RR-TB), which can reasonably be extrapolated to this context.^9^ Next, we observed that treatment with systemic corticosteroids alone, without ATT, might lead to a tendency for slightly faster initial uveitis resolution (partial or complete), even though the difference is not statistically significant. However, at six months, fewer patients in this group, compared to the ATT group, were able to taper treatment to 8 mg prednisone equivalent or two drops or fewer of prednisolone acetate daily. Once tapering below these thresholds and achieving complete uveitis resolution, uveitis relapse was more frequently observed in this non-ATT treated group. This suggests that intraocular inflammation may not be fully controlled without ATT, highlighting the importance of assessing treatment outcomes not only by uveitis resolution end point but also by the risk of uveitis relapse over an extended follow-up period. Nevertheless, we observed that half of the patients in the control group, who did not receive ATT, still achieved complete uveitis resolution. Additionally, the varied adverse events observed following ATT treatment emphasise the need for close monitoring and follow-up visits in both uveitis clinics and with internists or pulmonologists throughout the treatment period. Based on our findings, we consider that the benefits of ATT outweigh the risks. However, in settings with low TB prevalence or transmission, where avoiding potential side effects may be preferable, withholding unnecessary ATT might offer greater benefit. Thus, the generalisability of our results may be limited in settings with lower TB prevalence and transmission.

The current trial has some limitations. First, referral bias may impact the generalisability of our findings in general settings, as the trial was conducted at a single tertiary eye care centre. We aimed to minimise this by including only patients without prior high-dose systemic corticosteroids or immunosuppressants that could alter disease progression. Second, we could only conduct a single-blind trial due to the lack of available placebo for the FDC ATT, meaning that participants were not masked, and no placebo was used. However, the attending uveitis specialist who served as the clinical grader determining the studied outcomes was masked; no participant-reported outcomes were included. Third, the obligatory baseline consultations with the internist-pulmonologist which included counselling on ATT initiation, which may partly explain why eight patients withdrew from the control group allocation. This decision could have also been influenced by their perceived severity of ophthalmic conditions. Consequently, we supplemented our primary analysis with per-protocol analysis, as well as analysis last treatment received, to assess the consequences for our trial results. In this trial, we could not assess whether a shorter duration of ATT (six months) would also result in a reduction of uveitis relapse compared to a nine-month duration, as all patients ultimately completed nine-month ATT regimen. Even though ATT is administered for nine months, the choice of 6 months as the primary time point is based on the majority of reported outcomes for ocular TB treatment. Moreover, we supplemented our analysis with a longer follow-up period as a secondary study outcome (e.g., relapse). We did not perform a dedicated analysis of the rate of systemic corticosteroid tapering, which may have influenced the outcomes. In terms of visual acuity outcomes, no differences were observed between the two treatment allocations; however, we did not assess for possible optic nerve toxicity related to ethambutol use.

A strength of our trial is its direct comparison between ATT and non-ATT treatment approaches as a first-line option for patients with uveitis of undetermined cause and positive IGRA—a comparison that was not yet explored and reported in current literature. Randomisation prevents indication-for-treatment bias and avoids potential baseline imbalances in both recognised and unrecognised confounders. This is important because prior observational retrospective studies may have biases, particularly in cases where patients with more severe disease received ATT.^14^^,^^19^ Previous findings from this retrospective study indicated that, ATT was more often prescribed in patients with a more severe uveitis presentation, which were also the patients experiencing a uveitis relapse more often.^34^

We did not analyse the agreement between QFT and TST in this trial, however we believe our findings remain applicable in settings where TST is the only available TB immunoreactivity test.

Given the preliminary nature of this phase 2 trial conducted at a single centre with a limited sample size, future studies should evaluate the efficacy and safety of ATT in IGRA-positive patients with uveitis of undetermined cause using a multi-centre design. Ideally, such studies should include diverse geographical settings, particularly low TB-endemic regions, to enhance generalizability and statistical power. Nonetheless, our results provide an important foundation for future research in this area. Additionally, the identification of biomarkers for better precision in determining which patients would benefit most from ATT is warranted.

In conclusion, we observed that an initial treatment approach incorporating a full course of ATT in IGRA-positive patients with uveitis of undetermined cause was beneficial for achieving complete uveitis resolution at six months and reducing uveitis relapse during follow-up after the initial six months. Adverse events were frequently encountered with either ATT or systemic corticosteroids treatment, highlighting the importance of a multidisciplinary approach in monitoring of treatment course.

## Contributors

RLDN and IP contributed equally to the study and shared first authorship. IP, RLDN, and MR conceived and designed the study. IP, RLDN, MR, RS, LE, MS, YA, MZW, EW, UAS, PJ, RE, and GS conducted the study, including acquisition, analysis, or interpretation of data. IP drafted the first version of the manuscript. RLDN, WAD, SMR, PMvH performed supervision on analysis and interpretation of the results. All authors critically revised the manuscript. All authors gave final approval of the manuscript. The corresponding author attests that all listed authors meet authorship criteria and that no others meeting the criteria have been omitted. Contributors who had access to the underlying data were RLDN, IP, EW, UAS, PJ, and RE. RLDN, IP, and EW accessed and verified the data presented in this study.

## Data sharing statement

De-identified participant data associated with this trial can be requested, and inquiries should be submitted to rina.ladistia@ui.ac.id for consideration. Access to anonymised data may be granted upon formal request.

## Declaration of interests

All authors declared no competing interest. This study was supported by RISPRO-LPDP (Riset Inovatif Produktif—Lembaga Pengelola Dana Pendidikan, grant number: RISPRO/KI/B1/KOM/5/15219/4/2020). The grant provider had no role in study design, data collection, analysis, interpretation, or manuscript preparation.

## Appendix A Supplementary data

Systematic Workup Uveitis

App 3 Ethical approval and legal permission

App 4 Protocol and statistical analysis plan
