# Supplementary material for: Antitubercular therapy for uveitis of undetermined cause with positive interferon-gamma release assay: a single-blind, single-centre, phase 2 randomised controlled trial
Source: eClinicalMedicine. 2025 Sep 17;88:103511. doi: 10.1016/j.eclinm.2025.103511 (PMC12475421; doi:10.1016/j.eclinm.2025.103511)
Supplement: Systematic Workup Uveitis [file mmc1.docx]

Step-wise uveitis diagnostic procedure

| **Initial step**  (Routine) | **Subsequent steps**  These consist of workups that were not performed during the initial step and may be selected based on the anatomical subtype of uveitis or combined with the first step if clinical clues are present. A multidisciplinary approach is warranted when systemic involvement is suspected. The third step is performed if no positive findings are obtained from the second step or if the results are inconclusive. | | |
| --- | --- | --- | --- |
| **1^st^ step** | **Uveitis anatomical subtype/group** | **2^nd^ step** | **3^rd^ step** |
| Routine systemic investigations:   1. Complete blood counts 2. Erythrocyte sedimentation rate or C-reactive protein 3. Tuberculin skin test (TST) or interferon gamma-release assay (IGRA) 4. Syphilis serological tests (VDRL/RPR, TPHA) 5. Chest X-ray 6. Liver and renal functions 7. HIV screening 8. T helper lymphocytes (CD4+   CD8+), if immunodeficiency is suspected or the presence of clinical presentations of cytomegalovirus retinitis  Additional investigations, if required:   1. Ocular fluid (aqueous tap) from the initial visit if infectious uveitis is suspected, as follows:  - Unilateral presentation - Granulomatous inflammation - Anterior uveitis with iris atrophy or increased intraocular pressure (IOP) - Immunocompromised patients - The presence of retinal focal lesion - The presence of extensive retinitis - No uveitis improvement with previous empiric/standard treatment.  1. Diagnostic vitrectomy may be performed if malignancy is suspected 2. The 2^nd^ step can be directly considered if there is a suspicion of a relevant clinical presentations. | **Acute anterior uveitis** | - HLA-B27 - IGRA, if not performed | - Aqueous fluid analysis if HLA-B27 is negative - Complete urine workups if there is a history of conjunctivitis with suspicion of reactive arthritis - If TINU (tubulointerstitial nephritis & uveitis) is suspected, beta-2 microglobulin from urine sample - β-2 microglobulin in urine sample and Angiotensin converting enzyme (ACE) for pediatric patients, adolescents, and young adults |
|  | **Chronic anterior uveitis** | - Angiotensin Converting Enzyme (ACE) - Chest CT - IGRA, if not performed | - HLA-B27 - Serology anti PGL1 for leprae suspicion |
|  | **Chronic granulomatous uveitis or multifocal choroiditis** | - Angiotensin Converting Enzyme (ACE) - Chest CT - IGRA, if not performed - Toxocariasis serology - Anti Toxoplasma IgG, IgM from peripheral blood and/or PCR from ocular fluid sample | If there are clinical indications only:   - Minor salivary gland biopsy - Bronchoscopy dan bronchoalveolar lavage - 18-FDG PET or 67Ga scintigraphy - Cryptococcus serology - Serology anti PGL1 |
|  | **Chronic intermediate uveitis** |  | - Lumbal puncture - Brain MRI |
|  | **Chronic posterior uveitis** | - Anti Toxoplasma IgG, IgM from peripheral blood and/or PCR from ocular fluid. - Angiotensin converting enzyme (ACE) - Chest CT - IGRA, if not performed - ANCA (if there is a suspicion of Wegener’s granulomatosis (WG) or granulomatosis with polyangitis (GPA)) - If clinically suspected: fungal investigations from ocular fluid | - Lumbal puncture for cytology - Brain MRI |
|  | **Panuveitis** | - Anti Toxoplasma IgG, IgM from peripheral blood and/or PCR from ocular fluid. - Angiotensin converting enzyme (ACE) - Chest CT - IGRA, if not performed | - Lumbal puncture - Brain MRI - HLA-B27 |
|  | **Isolated retinal vasculitis** | - IGRA, if not performed - Complement factors - Antinuclear antibody (ANA) - Antiphospholipid antibody - Anti cytoplasmic antineutrophil antibody (ANCA) | - Angiotensin converting enzyme (ACE) - Complete urine workups |
|  | **Severe uveitis recalcitrant to immunosuppressants** | - IGRA, if not performed - Ocular fluid (aqueous tap/vitrectomy): for malignancy investigations or infection | - Re-vitrectomy (if malignancy is suspected) - Brain MRI - Angiotensin converting enzyme (ACE) |
|  | **Scleritis** | - IGRA - ANCA - Rheumatoid factor /anti-CCP - ANA | - Complete urine workups - HLA-B27 - Angiotensin converting enzyme (ACE) - Anti HCV - HbsAg - C3, C4 |
|  | **Paediatric cases** | - IGRA, if not performed - ANA - If immunodeficiency is suspected (IgG, IgM) anti Toxoplasma, CMV, HSV. Ocular fluid analysis if indicated. | - Complete urine workups - HLA-B27 |
