# Supplementary material for: Antitubercular therapy for uveitis of undetermined cause with positive interferon-gamma release assay: a single-blind, single-centre, phase 2 randomised controlled trial
Source: eClinicalMedicine. 2025 Sep 17;88:103511. doi: 10.1016/j.eclinm.2025.103511 (PMC12475421; doi:10.1016/j.eclinm.2025.103511)
Supplement: App 3 Ethical approval and legal permission [file mmc2.pdf]

UNIVERSITAS INDONESIA
FAKULTAS KEDOKTERAN

Gedung Fakultas Kedokteran UI
Jl. Salemba Raya No.6, Jakarta 10430
PO.Box 1358
T. 62.21.3912477, 31930371, 31930373, 3922977, 3927360, 3153236,
F 62 21 3912477, 31930372, 3157288,
E. humas@fk.ui.ac.id, office@fk.ui.ac.id
fk.ui.ac.id

Nomor : KET- 616 /UN2.F1/ETIK/PPM.00.02/2021

KETERANGAN LOLOS KAJI ETIK
ETHICAL APPROVAL

Komite Etik Penelitian Kesehatan Fakultas Kedokteran Universitas Indonesia – RSUPN Dr. Cipto Mangunkusumo dalam upaya melindungi hak asasi dan kesejahteraan subjek penelitian kedokteran, telah mengkaji dengan teliti protokol penelitian yang berjudul:

The Ethics Committee of the Faculty of Medicine, University of Indonesia – Cipto Mangunkusumo Hospital with regards of the Protection of human rights and welfare in medical research, has carefully reviewed the research entitled:

"Efektivitas Obat Anti Tuberkulosis sebagai Terapi Uveitis Idiopatik IGRA Positif: Penelitian Uji Klinis Terandomisasi."

Protocol Number : **21-04-0437**

Peneliti Utama
Principal Investigator : **dr. Rina La Distia Nora, Sp.M(K), Ph.D**

Nama Institusi
Name of the Institution : **Ilmu Kesehatan Mata FKUI -RSCM Kirana**

Lokasi Penelitian
Site : **Rumah Sakit Umum Pusat Nasional Cipto Mangunkusumo, Jakarta**

Tanggal Persetujuan
Date of Approval : 2 1 JUN 2021
(valid for one year beginning from the date of approval)

Dokumen Disetujui
Document Approved : **Proposal Penelitian, Version 0.3 tanggal 16 Juni 2021**
Lembar Penjelasan kepada Calon Subjek, Version 0.3 tanggal 16 Juni 2021

dan telah menyetujui protokol berikut dokumen terlampir.
and approves the above mentioned protocol including the attached document.

Ditetapkan di : Jakarta
Specified in

Ketua
Chair

UNIVERSITAS INDONESIA FAKULTAS KEDOKTERAN

Prof. dr. Rita Sita Sitorus, Ph.D., Sp.M(K)

** Peneliti berkewajiban
1. Menjaga kerahasiaan identitas subjek penelitian.
2. Memberitahukan status penelitian apabila:
   a. Setelah masa berlakunya keterangan lolos kaji etik, penelitian masih belum selesai, dalam hal ini *ethical approval* harus diperpanjang. Harap pengajuan perpanjangan etik dilakukan 2 minggu sebelum masa aktif lolos kaji etik habis.
   b. Penelitian berhenti ditengah jalan.
3. Melaporkan kejadian serius yang tidak diinginkan (*serious adverse events*).
4. Peneliti tidak boleh melakukan tindakan apapun pada subjek sebelum protokol penelitian mendapat lolos kaji etik dan sebelum memperoleh *informed consent* dari subjek penelitian.
5. Menyampaikan laporan akhir, bila penelitian sudah selesai.
6. Cantumkan nomor protokol ID pada setiap komunikasi dengan KEPK FKUI-RSCM.

Scanned with CamScanner

KEMENTERIAN KESEHATAN
DIREKTORAT JENDERAL PELAYANAN KESEHATAN
RSUP NASIONAL Dr. CIPTO MANGUNKUSUMO
Jalan Diponegoro No. 71 Jakarta 10430 Kotak Pos 1086
Call Center : 1500135 Fax : (021) 3148991, 3914661 *Website* : www.rscm.co.id

RSCM

NOTA DINAS
NOMOR : LB.02.01/2.6.1/0702/2021

Yth.	:	1. Kepala Instalasi Pelayanan Kesehatan Mata Terpadu RSCM Kirana<br>2. Ketua Kelompok Staff Medis Kesehatan Mata
Dari	:	Kepala Instalasi Pengelolaan Inovasi dan Kekayaan Intelektual
Hal	:	Persetujuan Izin Penelitian
Tanggal	:	19 Juli 2021

Bersama ini kami sampaikan, penelitian :

No. Agenda	:	6613
Nama	:	dr. Rina La Distia Nora, SpM(K), Ph.D
Unit Kerja	:	KSM Kesehatan Mata
No. Telp	:	0811198910
Judul	:	Efektivitas Obat Anti Tuberkulosis sebagai Terapi Uveitis Idiopatik IGRA Positif : Penelitian Uji Klinis Terandomisasi
Lokasi	:	Instalasi Pelayanan Kesehatan Mata Terpadu RSCM Kirana

Pada prinsipnya kami mengijinkan, selanjutnya agar peneliti dapat meregistrasikan penelitiannya dan menginput data pasien penelitian di link : http://his.rscm.co.id/his/ dengan menggunakan akun EHR.

Demikian kami sampaikan. Atas perhatian dan kerjasamanya, diucapkan terima kasih.

Dr. dr. Andri MT Lubis, SpOT(K)

Tembusan Yth :
1. Plt. Direktur SDM, Pendidikan dan Penelitian
2. Direktur Pelayanan Medik, Keperawatan dan Penunjang
3. Ka. Bagian Pendidikan dan Penelitian
4. Koord. Penelitian dan Pengembangan, KSM Kesehatan Mata
5. Peneliti yang bersangkutan

Catatan :
* **Surat persetujuan ijin penelitian berlaku satu tahun dari tanggal persetujuan.**
** Peneliti berkewajiban
1. Menjaga kerahasiaan identitas subyek penelitian.
2. Memberitahukan status penelitian apabila
   a. Setelah masa berlakunya persetujuan ijin penelitian, penelitian masih belum selesai, dalam hal ini persetujuan ijin penelitian harus diperpanjang.
   b. Penelitian berhenti di tengah jalan.
3. Melaporkan KTD, KNC dan kejadian serius yang tidak diinginkan (*serious adverse event*) ke Komite Mutu, Keselamatan dan Kinerja (KMKK) dan Komite Etik Penelitian Kesehatan FKUI-RSCM.
4. Peneliti tidak boleh melakukan tindakan apapun pada subyek sebelum mendapat lolos kaji etik, surat persetujuan ijin penelitian dan sebelum memperoleh informed consent dari subyek penelitian.
5. Menyampaikan laporan akhir, bila penelitian sudah selesai.

Scanned with CamScanner
