# Supplementary material for: Antitubercular therapy for uveitis of undetermined cause with positive interferon-gamma release assay: a single-blind, single-centre, phase 2 randomised controlled trial
Source: eClinicalMedicine. 2025 Sep 17;88:103511. doi: 10.1016/j.eclinm.2025.103511 (PMC12475421; doi:10.1016/j.eclinm.2025.103511)
Supplement: App 4 Protocol and statistical analysis plan [file mmc3.pdf]

PROTOCOL

This document contains the following items:

1. The original protocol, final protocol, and summary of changes
   Original protocol: page 1
   Final protocol: page 32
   Summary of changes: page 64

2. The original statistical analysis plan, final statistical analysis plan, and summary of changes
   Original statistical analysis plan: page 65
   Final statistical analysis plan: page 73
   Summary of changes: page 82

UNIVERSITAS INDONESIA

RESEARCH PROTOCOL

Version 1.0

The Effectivity of Anti-Tuberculosis Therapy in Idiopathic Uveitis With Positive *Interferon Gamma Release Assay* (IGRA): A Randomized Clinical Trial

Principal investigator:

dr. Rina La Distia Nora, Sp.M(K), Ph.D

Research team members:

dr. Ikhwanuliman Putera, Sp.M

Prof. Dr. dr. Ratna Sitompul, SpM(K)

Dr. dr. Made Susiyanti, SpM(K)

Dr. dr. Lukman Edwar, SpM(K)

dr. Yulia Aziza, SpM

Mei Riasanti, S.Si

Dr. dr. Aria Kekalih, M.T.I

Luluk Yunaini, S.Si, M.Biomed

Andriansjah, S.Si, M.Biomed, PhD

Dr. Andi Yasmon, S.Pi, M.Biomed

dr. Priscilla Jessica

dr. Ulifna Alfiya Sifyana

Department of Ophthalmology

Faculty of Medicine University of Indonesia

Cipto Mangunkusumo Hospital

Jakarta

2021

TABLE OF CONTENTS (Version 1.0)

List of abbreviations 3

Protocol summary 4

- Introduction and rationale 4
- Objective(s) 4
- Study design and population 5
- Intervention 5
- Main study parameters (endpoints) 6
- Nature and extent of the burden and risks 7

1. Introduction and Study Objectives 8
2. Study type 9
3. Study Population 10
4. Methods 12
5. Unexpected discoveries 15
6. Statistical Analysis 16
7. Recruitment and consent 17
8. Handling and storage of data and images 18
9. Handling and storage of human material 19
10. Publication 20
11. References 20
12. Attachments 23

LIST OF ABBREVIATIONS

COTS	The Collaborative Ocular Tuberculosis Study
LTBI	Latent tuberculosis infection
Mtb	*Mycobacterium tuberculosis*
OTB	Ocular Tuberculosis
PCR	Polymerase Chain Reaction
QFT	QuantiFERON-Gold TB
IGRA	Interferon Gamma Release Assay
SUN	The Standardization of Uveitis Nomenclature
TB	Tuberculosis
TST	Tuberculin Skin Test
ATT	Anti-Tubercular Therapy
NEI	National Eye Institute
RCT	Randomized Controlled Trial
MTA	Material Transfer Agreement
PBMC	Peripheral Blood Mononuclear Cells
RNA	Ribonucleic Acid

PROTOCOL SUMMARY

Introduction and Rationale:

Among the infectious causes of uveitis, TB is an etiology that must always be considered. In countries with a high incidence of TB, such as Indonesia, uveitis associated with concurrent active systemic TB accounts for 8% of newly referred uveitis cases in our setting. Interestingly, among patients with uveitis of undetermined cause, some exhibited TB immunoreactivity as detectable through interferon-gamma release assays (IGRA) or tuberculin skin tests (TST). Moreover, the proportion of such patients exceeded the rate of positive TB immunoreactivity in the general population, raising concerns that the presenting uveitis may represent a true manifestation of extrapulmonary TB in the eye.

Before the current trial, multiple retrospective studies reported treatment outcomes in the management of TB uveitis. There is no gold standard for diagnosing TB uveitis, partly due to the suboptimal diagnostic performance of current ocular fluid polymerase chain reaction (PCR) tests for detecting *Mycobacterium tuberculosis* (*Mtb*) genomes in ocular fluid samples. Most studies evaluating treatment outcome included both uveitis patients with proven active systemic TB and those showing TB immunoreactivity after other causes had been excluded. As previously summarized, treatment with antitubercular therapy (ATT) in these patients demonstrated an overall trend toward a better resolution of uveitis but failed to achieve statistical significance. Cautious interpretation is warranted, as our retrospective analysis suggests that the severity of presenting uveitis might influence treatment decisions regarding the initiation of ATT, complicating the interpretation of the actual benefits of ATT in this context. Of note, based on the opinions of uveitis experts, in the absence of active systemic TB, there is no strong consensus on initiating ATT unless ophthalmologic presentations such as choroidal granuloma or serpiginous-like choroiditis are observed.

Objective:

To determine whether ATT would eventually improve outcomes compared to treatment without ATT in terms of uveitis resolution and relapse, during subsequent follow-up of patients with uveitis of undetermined cause who were showing TB immunoreactivity, based on QuantiFERON-TB Gold Plus (QFT) test positivity.

Study design & study population:

Single blind randomized controlled trial (RCT) – single center

All newly referred uveitis patients will be evaluated for eligibility at the outpatient uveitis clinic, Department of Ophthalmology, Faculty of Medicine, Universitas Indonesia - Cipto Mangunkusumo Hospital, Jakarta, Indonesia.

Inclusion criteria are: (1) adults (age ≥18 years) with newly diagnosed, clinically active uveitis of undetermined cause, following standardized workups at our center (see Supplementary file); (2) positive QFT test result (>0.35 U/mL; Kimia Farma Laboratory, Depok, PT. Kimia Farma Diagnostika); (3) local residence in Jakarta, Bogor, Depok, Tangerang, or Bekasi; and (4) consent to participate in the study through the entire monitoring period. Exclusion criteria were: (1) confirmed active systemic TB or living with an active TB patient; (2) HIV-positive status; (3) visual acuity less than hand movement with signs of phthisis on ophthalmological examinations and ultrasound; (4) impaired liver function or other systemic conditions, as assessed by the internist-pulmonologist, that precluded eligibility for ATT; (5) pregnancy, and (6) recent antibiotic use, including ATT, or systemic steroid use exceeding 8 mg prednisone equivalent daily within the last 14 days.

Ocular PCR will be performed in selected cases based on the clinical judgement of the attending uveitis specialist. This will be done (1) to confirm the clinical diagnosis, or (2) to exclude other differential diagnosis. If it is performed, any positive results will result in exclusion from trial entry.

After eligibility assessment, participants will be randomly assigned in a 1:1 ratio to one of the two treatment groups without stratification factors. Randomization will be conducted by a dedicated research manager using block randomization (block size of 4) to ensure equal distribution between the groups.

Intervention:

All included participants will receive immunosuppressants at the discretion of the attending uveitis specialists. There will be no restrictions on topical or local treatments throughout the entire follow-up period, including surgical interventions, if indicated.

- *Arm 1: ATT*

The full course of ATT will consist two months in the intensive phase, followed by seven months in the continuation phase (2RHZE/4RH).

- *Arm 2: Control group*

The treatment for the control group primarily involved immunosuppressants without ATT.

Main study parameters/endpoints:

<u>Primary Outcome</u>

- Complete resolution of uveitis at the patient level at six months.

Criteria for complete uveitis resolution (applied to both eyes: patient-level outcome): (1) fewer than or equal to 0.5+ anterior chamber cells according to the Standardized Uveitis Nomenclature (SUN) grading system, fewer than or equal to 0.5+ vitreous haze based on clinical grading using the NEI (National Eye Institute) scale, and no active retinal or choroidal lesions; and (2) no more than 7.5 mg of oral prednisone daily (i.e., methylprednisolone at 6 mg or less daily) and fewer than or equal to 2 drops of prednisolone acetate 1% (or equivalent) per day. These criteria have to be maintained for at least 90 days following the visit when the first complete uveitis resolution was recorded.

If there is insufficient follow-up after resolution, maintained uveitis resolution is still considered. Participants who meet criterion 1 but not criterion 2 will be classified as having partial uveitis resolution, and for the primary endpoint classification, they will be considered to have non-complete uveitis resolution.

<u>Secondary outcome</u>

- The details of treatment failure (partial resolution or non-responsive)
- Time to uveitis resolution
- Uveitis relapse following complete resolution,

Nature and extent of the burden and risks associated with participation, benefit and group relatedness:

Adverse events

Adverse events will be recorded during the trial. Baseline consultation and workup include assessment of potential adverse events risks. Participants will be closely monitored by the internal medicine department. Participants can withdraw their consent at any time during the trial.

1. INTRODUCTION AND STUDY OBJECTIVES

1.1. Introduction

Uveitis is an umbrella term for intraocular inflammation caused by infectious or non-infectious causes[1, 2]. Currently, more than 30 diseases associated with uveitis have been identified[1, 2]. However, more than one-third of clinical uveitis cases lack definitive etiologies, falling under the category of "uveitis of undetermined cause" or idiopathic[2, 3]. In clinical practice, uveitis is considered infectious until proven otherwise[4, 5]. Therefore, systematic investigations using various diagnostic tests, along with multidisciplinary consultations are often required[6, 7]. In cases of non-infectious uveitis, immunosuppressants—starting with steroids administered locally, systemically, or both—is the mainstay of treatment[5]. For infectious uveitis, appropriate antimicrobial treatment is essential for achieving uveitis resolution[2]; otherwise, the condition maintains and may worsens even into blindness such as in tubercular (TB) uveitis [8].

Among the infectious causes of uveitis, TB is an etiology that must always be considered. In countries with a high incidence of TB, such as Indonesia, uveitis associated with concurrent active systemic TB accounts for 8% of newly referred uveitis cases in our setting[9]. Interestingly, among patients with uveitis of undetermined cause, some exhibited TB immunoreactivity as detectable through interferon-gamma release assays (IGRA) or tuberculin skin tests (TST). Moreover, the proportion of such patients exceeded the rate of positive TB immunoreactivity in the general population[9, 10], raising concerns that the presenting uveitis may represent a true manifestation of extrapulmonary TB in the eye.

Before the current trial, multiple retrospective studies reported treatment outcomes in the management of TB uveitis[11]. There is no gold standard for diagnosing TB uveitis[12, 13], partly due to the suboptimal diagnostic performance of current ocular fluid polymerase chain reaction (PCR) tests for detecting *Mycobacterium tuberculosis* (Mtb) genomes in ocular fluid samples[7, 14]. Most studies evaluating treatment outcome included both uveitis patients with proven active systemic TB and those showing TB immunoreactivity after other causes had been excluded. As previously summarized, treatment with antitubercular therapy (ATT) in these patients demonstrated an overall trend toward a better resolution of uveitis but failed to achieve statistical significance[11, 15]. Cautious interpretation is warranted, as our

retrospective analysis suggests that the severity of presenting uveitis might influence treatment decisions regarding the initiation of ATT, complicating the interpretation of the actual benefits of ATT in this context [16]. Of note, based on the opinions of uveitis experts, in the absence of active systemic TB, there is no strong consensus on initiating ATT unless ophthalmologic presentations such as choroidal granuloma or serpiginous-like choroiditis are observed[13].

In light of this, we conducted a randomized clinical trial to determine whether ATT would eventually improve outcomes compared to treatment without ATT in terms of uveitis resolution and relapse, during subsequent follow-up of patients with uveitis of undetermined cause who were showing TB immunoreactivity, based on QuantiFERON-TB Gold Plus (QFT) test positivity.

1.2. Objective (s)

Primary objective

To compare the clinical effectivity of initial treatment approach that include ATT in addition to immunosuppressants versus immunosuppressants alone in undetermined cause of uveitis cases with positive IGRA test.

Secondary objectives

1. To explore details of treatment details of treatment failure (partial resolution or non-responsive), time to uveitis resolution, and uveitis relapse following complete resolution.
2. To explore relevant biomarkers at baseline, during, and at the end of primary end point (6 months follow-up).

1.3 Hypothesis

Patients that received initial treatment incorporating a full course of ATT in QFT-positive patients with uveitis of undetermined cause is achieving complete uveitis resolution at six months and reducing uveitis relapse during follow-up after the initial six months.

2. Study type

2.1 Study type:

☐ Retrospective

☒ Prospective

☐ Combination Retrospective/Prospective

2.2 Check all the applicable boxes:

☐ Medical records (re-use of data from healthcare, including AI)

☐ Case report

☐ Re-use data from research

☐ Evaluations of quality of healthcare (retrospective)

☐ Research with additional use of residual material from regular healthcare

☐ Research with re-use of human materials from research or existing biobank

☐ Research with human materials without biobank

☐ De novo biobank (human material obtained without burdensome or invasive procedures)

☒ Interventional

☐ Phase IV research

☐ Healthcare evaluation research (prospective)

☐ Medical devices

☐ In Vitro Diagnostic Tests

☐ Other research, *describe*

3. Study population

a. Population

Patients diagnosed with Uveitis of undetermined cause with positive TB immunoreactivity (IGRA) at the outpatient clinics in Dr. Cipto Mangunkusumo National Central Public Hospital, Jakarta.

b. Inclusion criteria:

1. Patient that is newly diagnosed with uveitis of unknown origin/idiopathic (as evidenced by a series of uveitis work-up tests) and tested positive for IGRA (>0.35 U/ml).
2. Age >18 years old.
3. Lives in Jakarta/Bogor/Depok/Tangerang/Bekasi area or willing to participate in research until the end of monitoring program.
4. Willing to participate in the research and sign the informed consent after receiving explanation regarding the research.

c. Exclusion criteria:
   1. Patients with positive aqueous tap examination results on one of the examination panels for the bacteria causing infectious uveitis according to the standard examination.
   2. Anterior uveitis patient with a positive HLA-B27 test result.
   3. The patient is proven to have active TB or lives in the same house with an active TB patient.
   4. Patients are included in the TB reactivation risk index group according to the 2018 WHO LTBI (Latent Tuberculosis Incident) Guideline.
   5. HIV positive patient.
   6. Patients with inactive uveitis at the first visit.
   7. Patients with visual acuity less than 1/300 or showing signs of preptisis based on ophthalmological examination and ultrasound of the eye.
   8. The patient has a history of previous ATT consumption.
   9. Patients with impaired liver function or other systemic conditions which according to the Internal Medicine Department are not eligible to receive ATT.
   10. The patient has a history of taking antibiotics in the last 1-2 weeks.
   11. The patient is not willing to sign the informed consent.
   12. The patient was pregnant at the first visit or was planning to become pregnant during the study period.

d. Sample size calculation

The sample size is calculated using a formula of two-independent proportion sample size calculation. The primary end point of uveitis resolution at 6 month is used for this sample size calculation.

Based on the Multicenter Uveitis Steroid Treatment (MUST) trial in non-infectious uveitis, the proportion of uveitis patients with active disease at six months following treatment was found to be 44%[17]. A 30% difference in uveitis resolution between the two groups is predetermined. Using a Zα of 1.96 and a Zβ of 0.84 (for a two-sided significance level of 5% and 80% power), the estimated sample size required for each treatment group will be 35 participants. With 10% increase to anticipate dropout, a total of 39-40 participants in each treatment group is required.

4. $$N1 = N2 = \left(\frac{Z\alpha\sqrt{2PQ}+Z\beta\sqrt{P1Q1+P2Q2}}{P1-P2}\right)^2$$

Zα (1.96), power (80%, Zβ = 0.84), P1 based on MUST trial 44% (Q2 = 56%). Estimated P1 – P2 = 30%. Thus, P2 = 14% (Q2 = 86%). As 10% drop out rate is being anticipated, N1 = N2 = 39 ~ 40 (total 78-80 participants)

e. (Planned) start date

Date start collection data: August 2021 (tentative after ethical and legal approval)

f. (Planned) end data

Date end collection data: 31 December 2022

4. Methods

4.1 *Please describe how you do conduct research and which methods are used, e.g.*

This single-blinded, randomized controlled trial was conducted at Cipto Mangunkusumo Hospital, a tertiary eye hospital in Jakarta, Indonesia.

All newly referred uveitis patients will be evaluated for eligibility at the outpatient uveitis clinic, Department of Ophthalmology, Faculty of Medicine, Universitas Indonesia - Cipto Mangunkusumo Hospital, Jakarta, Indonesia.

Eligible participants will undergo standardized workups and received consultations from the participating internist-pulmonologist at our hospital. The internist-pulmonologist will be unaware of the randomization and provided all patients with the necessary information and counseling regarding ATT prescription and initiation.

Treatment arms

Study arms	Intervention	Control
**Anti-Tuberculosis**	Yes	No
**Therapy**	Dosage form: ATT fixed-dose combination (FDC). FDC intensive phase containing 150 mg rifampicin, 75 mg isoniazid, 400 mg pyrazinamide, and 275 mg ethambutol), while FDC continuation phase containing rifampicin-isoniazid. Dosage: according to body weight, 30-37 kg: 2 tablets, 38-54 kg: 3 tablets, 55-70 kg: 4 tablets, more than 70 kg: 5 tablets. Frequency: Intensive phase: once daily. Continuation phase: 3 times/week. Duration: ≥ 6 months* (2 months of FDC intensive phase + 4 months of FDC continuation phase). Treatment duration will be determined by the pulmonologist according to local guidelines. Once decided, the duration will be standardized for all included participants, with exceptions for those requiring ATT adjustments due to other factors (e.g., adverse effects, allergies, etc.).	-

**Immunosuppressants**	Possible (Depending on the clinical presentation and the clinical judgment of the blinded attending uveitis specialist)	Yes
	Oral methylprednisolone at a dosage of 0.8 mg/kg of body weight per day (maximum of 56 mg/day), which will be tapered gradually based on the intraocular inflammation observed. This dose is equivalent to 1 mg/kgBW/day (max 60 mg/day).<br>Tapering of oral methylprednisolone involve reducing the dose by 8 mg for doses above 20 mg and by 4 mg for doses below 20 mg.<br>All patients receiving systemic steroids, including those in the ATT group, will also receive vitamin D, calcium supplements, and gastric protectors (e.g., omeprazole or antacids).<br>Local steroids (e.g., steroid drops) will be prescribed based on the clinical presentation.<br>Disease-modifying anti-rheumatic drugs (DMARDs) may be prescribed based on the clinical judgment of the attending pulmonologist/internist-immunologist.	

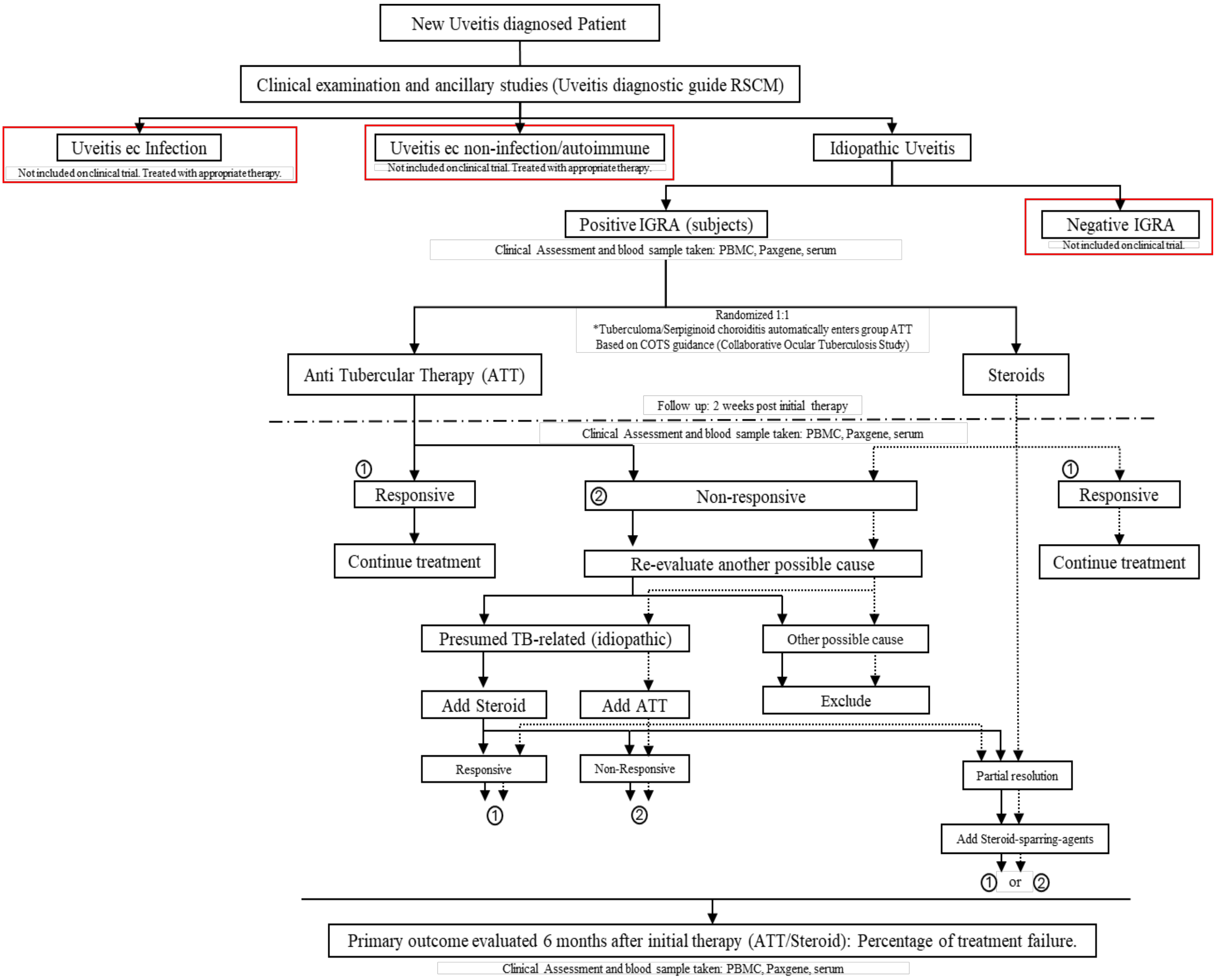

5. Unexpected discoveries

- Is there a chance of unexpected discoveries?

  ☐ Yes

  ☒ No

- If yes, describe the procedures, who will be notified, how the subjects are notified.

6. Statistical analysis

- The intention-to-treat (ITT) approach

All analyses will be first conducted using the ITT approach. The ITT population will include all participants who underwent randomization, regardless of any treatment switching that occurred. For participants who missed the six-month visit (±28 days), if any, last observation carried forward (LOCF) imputation will be performed..

- The per-protocol approach

The per-protocol population will include all participants who were randomized, excluding those who do not have six-month visits or deviated from the treatment protocols (e.g., non-compliant, experience severe adverse effect, etc). If any, participants who switch treatment will also be excluded in this per-protocol analysis.

For patients receiving ATT, missing 2 weeks of medication will be considered non-compliance (protocol deviation). The pulmonologist will record the prescribed ATT and count the remaining drugs (i.e., ATT) at each visit to determine treatment non-compliance. For participants receiving systemic immunosuppressants, missing more than 1 week of the prescribed regimen will be considered non-compliance. Topical immunosuppressants will not be taken into account when determining compliance.

6.1. Main study parameter/endpoint:

Response to treatment

At 6 month follow-up:

Criteria for complete uveitis resolution applied to both eyes, in cases with bilateral uveitis, included[18]: (1) fewer than or equal to 0.5+ anterior chamber cells according to the Standardized Uveitis Nomenclature (SUN) grading system, fewer than or equal to 0.5+ vitreous haze based on clinical grading using the NEI (National Eye Institute) scale, and no active retinal or choroidal lesions; and (2) no more than 7.5 mg of oral prednisone daily (i.e., methylprednisolone at 6 mg or less daily) and

fewer than or equal to 2 drops of prednisolone acetate 1% (or equivalent) per day. Treatment will be categorized as a failure if subject did not meet the following criteria at the 6-month follow-up.

6.2.Additional study parameters (endpoints)

a. Time to uveitis resolution: Calculated from the time of randomization. These criteria had to be maintained for at least 90 days following the visit when the first complete uveitis resolution was recorded. If there was insufficient follow-up after resolution, maintained uveitis resolution was still considered. Participants who met criterion 1 but not criterion 2 were classified as having partial uveitis resolution (non-complete uveitis resolution)

a. Uveitis relapse (subsequent follow-up, beyond 6 months). Uveitis relapse is defined as any worsening of ocular inflammation (including a two-step increase in anterior chamber or vitreous cells as per the SUN grading system) or the occurrence of clinically new inflammatory activity (such as choroidal or retinal lesions) that necessitated a modification of local or systemic uveitis treatment after a minimum of 90 days of complete uveitis resolution at the patient level[19, 20].

b. Time to relapse was calculated from the first visit showing inactive uveitis until the first notification of uveitis relapse.

c. Biomarkers associated with trial from samples obtained from study participants. Exploratory investigations may be conducted as supplementary analyses. Samples will be collected and stored following the institution's standardized procedures for biobanking of human study materials. Details of further analyses will be provided in a separate document.

7 Recruitment and consent

7.1 Will the subjects be asked for informed consent?

☒ Yes *(Upload Patient Information Letter and Informed Consent)*

☐ No, only anonymous data is used, i.e. the data can never be traced back to an individual subject

☐ No, this research will be performed under the exception consent *(Upload form Care for data Template)*

☐ Other (e.g. partly, indirectly) *Please describe the situation.*

7.2 *If yes, please give a description of the recruitment and informed consent procedures. How and by whom (investigator, supervising doctor, other person) participants will be informed about the study and asked for their consent, how much time will they be given to consider the decision.The patient information letter with informed consent form should be attached as a separate document.*

The principal investigator or a designated team member responsible for participant recruitment will obtain informed consent directly and in person. Participants, or their legal guardian if applicable, must provide consent before any intervention associated with the trial is administered. Participants have the right to ask questions, accept, or refuse any intervention related to the trial. Additionally, they have the right to withdraw consent at any time.

7.3.If no, exception consent: *describe how it is safeguarded that subjects are excluded who have objected against the re-use of their data, human material, images.*

Not applicable

8. Handling and storage of data and images

8.1 *Describe how subject's privacy is protected. Describe how, when and by whom data is coded, and how the key table is safeguarded.*

Data will be collected using a designated research form and entered into an electronic database. There will be two databases: The first database will contain a unique ID linked to a particular participant's data in the trial. The second database will include a newly generated ID and data relevant to the trial, without any patient names, initials, or medical record numbers. Anonymity will be preserved, and subject identification will be based on a specific code assigned to each participant. The master dataset, which is password-protected and links specific codes to individual patients, will only be accessible to selected investigators.

8.2 *Describe how data is stored (i.e. which data management system/data capture system), who has access to the coded source data, how long data will be kept, which steps are*

taken to ensure data security, what happens with the data after the research has been completed.

The data from the medical records will be manually input and stored into electronic data capture (CASTOR) with a pre-set database. Each investigator will have a specific role to view, edit, and validate the data. The data will only be accessed for this research purpose for this specific study duration. All investigators guarantee that the entered data will not be sent or used outside this study purpose. Once this study completed, any further analysis with the data should obtain a new permission.

8.3 *Describe how images are stored, how the subject's privacy is protected, what happens with images after the research has been completed.*

All data, including images, will be stored in CASTOR during this study period. All data will be stored without patient name, medical records number, or any identifiable clues toward the particular patient.

9. Handling and storage of human material

IGRA testing will be performed as part of the study, separate from the routine procedures conducted by the hospital. Blood collection for this test will be combined with the collection of participant samples for biobanking and biomarker discovery.

Blood Sample Collection: Conducted at the Department of Ophthalmology, Faculty of Medicine University of Indonesia, by trained nurses/staff.

- Procedure:
    - The patient sits with the arm extended.
    - The staff washes hands and wears non-sterile gloves.
    - Identifies a clearly visible vein.
    - A tourniquet is applied 10 cm above the phlebotomy site.
    - Asepsis is performed using 70% alcohol swab and allowed to dry.
    - A needle is inserted at a 15-30° angle until blood flashback is observed.
    - A 20 mL blood sample is collected.

- Sample Storage and Processing:

Samples for biobanking will be stored as PBMC, serum, and whole blood (PAXgene) in the laboratory.

- PAXgene samples are stored at low temperatures per protocol.
- PBMC is isolated using Ficoll gradient in a Biosafety Level II Cabinet and stored in liquid nitrogen.
- RNA Isolation and Genetic Analysis:
- PBMC is thawed in 37°C water (<5 minutes) before further processing.
- RNA is extracted from PBMC and whole blood using an RNA Extraction Kit.

When the vacutainer arrives at the processing laboratory, it will be processed immediately. The aliquot sample will be stored at 4°C during processing and transferred to ultra-low temperature long-term storage using liquid nitrogen as soon as possible after processing to ensure that all samples are preserved in cryopreservation. Any subsequent material transfer will follow the Material Transfer Agreement (MTA) procedure under national regulations (Ministry of Health of the Republic of Indonesia No. 657/MENKES/PER/VIII/2009).

10. Publication

Do you have the intention to submit the study results in a manuscript for publication in a journal:

☒ Yes

☐ No, *please motivate*

11. References

1. Burkholder BM, Jabs DA. Uveitis for the non-ophthalmologist. *Bmj* 2021; **372:** m4979.

2. Jabs DA, Busingye J. Approach to the diagnosis of the uveitides. *Am J Ophthalmol* 2013; **156**(2)**:** 228-236.

3. Tsirouki T, Dastiridou A, Symeonidis C, Tounakaki O, Brazitikou I, Kalogeropoulos C, Androudi S. A Focus on the Epidemiology of Uveitis. *Ocul Immunol Inflamm* 2018; **26**(1)**:** 2-16.

4. Agrawal R, Thng ZX, Gupta A, Toy BC, Dick AD, Smith JR *et al.* Infectious Uveitis: Conversations with the Experts. *Ocul Immunol Inflamm* 2023; **31**(7)**:** 1333-1341.

5. Writers AM. Manage non-infectious uveitis in adults with individualized immunosuppressive therapy and regular monitoring. *Drugs & Therapy Perspectives* 2016; **32**(6)**:** 244-248.

6. Sève P, Cacoub P, Bodaghi B, Trad S, Sellam J, Bellocq D *et al.* Uveitis: Diagnostic work-up. A literature review and recommendations from an expert committee. *Autoimmunity Reviews* 2017; **16**(12)**:** 1254-1264.

7. Ludi Z, Sule AA, Samy RP, Putera I, Schrijver B, Hutchinson PE *et al.* Diagnosis and biomarkers for ocular tuberculosis: From the present into the future. *Theranostics* 2023; **13**(7)**:** 2088-2113.

8. Basu S, Das T. Pitfalls in the management of TB-associated uveitis. *Eye (Lond)* 2010; **24**(11)**:** 1681-1684.

9. La Distia Nora R, Sitompul R, Bakker M, Susiyanti M, Edwar L, Sjamsoe S *et al.* Tuberculosis and other causes of uveitis in Indonesia. *Eye (Lond)* 2018; **32**(3)**:** 546-554.

10. Shrestha AB, Siam IS, Tasnim J, Dahal A, Roy P, Neupane S *et al.* Prevalence of latent tuberculosis infection in Asian nations: A systematic review and meta-analysis. *Immun Inflamm Dis* 2024; **12**(2)**:** e1200.

11. Betzler BK, Putera I, Testi I, La Distia Nora R, Kempen J, Kon OM *et al.* Anti-tubercular therapy in the treatment of tubercular uveitis: A systematic review and meta-analysis. *Surv Ophthalmol* 2023; **68**(2)**:** 241-256.

12. Agrawal R, Testi I, Mahajan S, Yuen YS, Agarwal A, Rousselot A *et al.* The Collaborative Ocular Tuberculosis Study (COTS) Consensus (CON) Group Meeting Proceedings. *Ocul Immunol Inflamm* 2020; **28**(sup1)**:** 85-95.

13. Agrawal R, Testi I, Bodaghi B, Barisani-Asenbauer T, McCluskey P, Agarwal A *et al.* Collaborative Ocular Tuberculosis Study Consensus Guidelines on the Management of Tubercular Uveitis-Report 2: Guidelines for Initiating Antitubercular Therapy in Anterior Uveitis, Intermediate Uveitis, Panuveitis, and Retinal Vasculitis. *Ophthalmology* 2021; **128**(2)**:** 277-287.

14. Vasconcelos-Santos DV, Zierhut M, Rao NA. Strengths and weaknesses of diagnostic tools for tuberculous uveitis. *Ocul Immunol Inflamm* 2009; **17**(5)**:** 351-355.

15. Putera I, Schrijver B, Ten Berge J, Gupta V, La Distia Nora R, Agrawal R *et al.* The immune response in tubercular uveitis and its implications for treatment: From anti-tubercular treatment to host-directed therapies. *Prog Retin Eye Res* 2023; **95:** 101189.

16. Putera I, Ten Berge J, Thiadens A, Dik WA, Agrawal R, van Hagen PM *et al.* Clinical Features and Predictors of Treatment Outcome in Patients with Ocular Tuberculosis from the Netherlands and Indonesia: The OculaR TB in Low versus High Endemic Countries (ORTEC) Study. *Ocul Immunol Inflamm* 2024**:** 1-12.

17. Multicenter Uveitis Steroid Treatment Trial Research G, Kempen JH, Altaweel MM, Holbrook JT, Jabs DA, Louis TA *et al.* Randomized comparison of systemic anti-inflammatory therapy versus fluocinolone acetonide implant for intermediate, posterior, and panuveitis: the multicenter uveitis steroid treatment trial. *Ophthalmology* 2011; **118**(10)**:** 1916-1926.

18. Rathinam SR, Gonzales JA, Thundikandy R, Kanakath A, Murugan SB, Vedhanayaki R *et al.* Effect of Corticosteroid-Sparing Treatment With Mycophenolate Mofetil vs Methotrexate on Inflammation in Patients With Uveitis: A Randomized Clinical Trial. *Jama* 2019; **322**(10)**:** 936-945.

19. Jabs DA, Nussenblatt RB, Rosenbaum JT, Standardization of Uveitis Nomenclature Working G. Standardization of uveitis nomenclature for reporting clinical data. Results of the First International Workshop. *Am J Ophthalmol* 2005; **140**(3)**:** 509-516.

20. Agrawal R, Agarwal A, Jabs DA, Kee A, Testi I, Mahajan S *et al.* Standardization of Nomenclature for Ocular Tuberculosis - Results of Collaborative Ocular Tuberculosis Study (COTS) Workshop. *Ocul Immunol Inflamm* 2020; **28**(sup1)**:** 74-84.

21. Majumder PD, Sudharshan S, Biswas J. Laboratory support in the diagnosis of uveitis. *Indian journal of ophthalmology* 2013; **61**(6)**:** 269-276.

22. Macarie SS, Kadar A. Eye involvement in ANCA positive vasculitis. *Rom J Ophthalmol* 2020; **64**(1)**:** 3-7.

Attachement 1. Informed consent *(in Bahasa as it was in the original file).*

LEMBAR PENJELASAN KEPADA CALON SUBJEK

Saya, dr. Rina La Distia Nora, SpM(K), PhD dan tim, dari Departemen Ilmu Kesehatan Mata FKUI - RSCM akan melakukan penelitian dengan judul **"Efektivitas obat anti tuberkulosis sebagai terapi uveitis idiopatik IGRA positif: Penelitian Uji Klinis Terandomisasi".**

Saya akan memberikan informasi kepada (Bapak/Ibu/Saudara) mengenai penelitian ini dan mengundang (Bapak/Ibu/Saudara) untuk menjadi bagian dari penelitian ini.

Bapak/lbu/Saudara yang terhomat,

Bapak/lbu/Saudara saat ini menderita uveitis, yaitu peradangan pada bagian dalam mata yang dicurigai terjadi akibat infeksi (virus, bakteri, atau parasit), proses autoimun, atau penyebab yang masih belum dapat ditentukan. Untuk uveitis yang tidak diketahui penyebabnya (idiopatik), kami mengelompokkannya berdasarkan hasil pemeriksaan tes IGRA: positif atau negatif. IGRA adalah pemeriksaan sampel darah untuk mengetahui apakah Bapak/lbu/Saudara pernah terpapar dengan kuman tuberkulosis. Kami ingin menganalisis pengaruh pemberian obat antituberkulosis (OAT) dibandingkan dengan obat steroid (terapi anti radang oral) selama 6 bulan pada Bapak/Ibu/Saudara, yang dinilai dari hilangnya peradangan pada mata.

Bapak/Ibu/Saudara dapat berpartisipasi dalam penelitian ini dengan cara menandatangani formulir ini. Jika Bapak/Ibu/Saudara setuju untuk berpartisipasi dalam penelitian ini, Bapak/Ibu/Saudara kapan saja dapat secara bebas mundur dari penelitian ini. Jika Bapak/Ibu/Saudara menolak untuk berpartisipasi atau mundur dari penelitian ini, keputusan tersebut tidak akan mempengaruhi hubungan Bapak/Ibu/Saudara dengan saya dan tidak akan berdampak pada pelayanan yang berlaku di rumah sakit ini.

Jika Bapak/Ibu/Saudara tidak mengerti tiap pernyataan dalam formulir ini, Bapak/Ibu/Saudara dapat menanyakannya kepada saya.

1. Tujuan penelitian

Penelitian ini bertujuan melihat dampak pemberian obat anti tuberkulosis (OAT) selama enam bulan dibandingkan dengan pemberian steroid (obat anti radang oral) dalam mengobati uveitis yang belum diketahui penyebabnya (idiopatik) namun disertai dengan hasil pemeriksaan IGRA positif (diartikan pernah terpapar dengan kuman tuberkulosis). Di samping itu, penelitian ini juga bertujuan mengetahui pola perjalanan penanda peradangan (yang dianalisis dari sampel darah) pada subjek yang mengalami uveitis idiopatik IGRA positif, mulai dari sebelum hingga selesai menjalani pengobatan.

2. Partisipasi dalam penelitian

Jika Bapak/Ibu/Saudara berkenan diikutsertakan dalam studi ini, Bapak/Ibu/Saudara akan menjalani pemeriksaan mata standar di poliklinik dilanjutkan dengan pengambilan sampel darah untuk pemeriksaan substansi penanda peradangan dalam darah yang berhubungan dengan peradangan pada mata, yang sampai saat ini masih terus diteliti.

Bapak/Ibu/Saudara akan dimasukan ke dalam satu dari dua kelompok yang menerima pengobatan oral yang berbeda secara acak, namun terapi standar tetes mata anti radang tetap diberikan kepada semua peserta. Pada kelompok pertama, kami akan berikan pengantar pemberian obat anti tuberkulosis (OAT) ke bagian Penyakit Dalam RSCM untuk dinilai apakah OAT dapat diberikan, dan dosisnya dihitung sesuai dengan berat badan Bapak/Ibu/Saudara. OAT akan diberikan untuk diminum setiap hari selama 6 bulan sesuai dosis. Bapak/Ibu/Saudara akan tetap menjalani kontrol pemeriksaan mata ke RSCM Kirana secara berkala. Sedangkan pada kelompok kedua, Bapak/Ibu/Saudara akan diberikan obat anti radang metilprednisolon yang dosisnya disesuaikan dengan berat badan Bapak/Ibu/Saudara. Pemberiannya akan dipantau dan dievaluasi secara berkala.

Sebagai catatan, bagi Bapak/Ibu/Saudara yang menerima terapi OAT, apabila selama 2-4 minggu awal terjadi perburukan pada kondisi mata Bapak/Ibu/Saudara, maka Bapak/Ibu/Saudara akan mendapatkan tambahan obat anti radang berupa metilprednisolon oral untuk mengendalikan peradangan. Sebaliknya, bagi Bapak/Ibu/Saudara yang menerima metilprednisolon dan setelah pemantauan dipertimbangkan pemberian OAT, maka Bapak/Ibu/Saudara akan kami pertimbangkan untuk pemberian obat tersebut dengan menilai kondisi penyakit. Obat lain seperti penekan imun (contohnya: azatioprin) juga dapat kami pertimbangkan untuk diberikan apabila diperlukan, dengan evaluasi oleh dokter penyakit dalam terlebih dahulu.

Bapak/Ibu/Saudara, baik yang mendapat OAT maupun yang mendapat obat metilprednosolon, akan kembali diambil sampel darahnya pada minggu ke-2 dan bulan ke-6 setelah pengobatan dimulai, untuk mengetahui perubahan pola penanda peradangan dalam darah Bapak/Ibu/Saudara mulai dari sebelum hingga selesai pengobatan.

3. Alasan memilih Bapak/Ibu/Saudara

Bapak/Ibu/Saudara kami undang sebagai subjek penelitian ini karena Bapak/Ibu/Saudara merupakan pasien RSCM Kirana yang baru terdiagnosis dengan uveitis idiopatik (peradangan pada mata yang belum diketahui penyebabnya) disertai dengan hasil IGRA yang positif (diartikan pernah terpapar dengan kuman tuberkulosis), berusia di atas 18 tahun, dan berdomisili di area Jabodetabek.

4. Prosedur penelitian

4a. Prosedur intervensi

1) Bapak/Ibu/Saudara akan menjalani prosedur pengambilan cairan bola mata yang dilakukan di ruang operasi untuk mencari tahu agen penyebab peradangan pada mata Bapak/Ibu/Saudara dengan langkah-langkah sebagai berikut:
    i. Bapak/Ibu/Saudara dalam posisi berbaring telentang
    ii. Dilakukan tindakan sterilisasi menggunakan betadine pada area mata yang sakit lalu area sekitar mata tersebut ditutup kain steril
    iii. Diberikan anestesi (obat bius) dalam bentuk tetes mata pada mata yang sakit, lalu setelah beberapa saat kelopak mata atas dan bawah diberi penyangga agar tidak mengganggu selama dilakukan tindakan
    iv. Dokter melakukan pengambilan cairan bola mata sebanyak 0.1-0.2 cc menggunakan jarum suntik berukuran 1 cc

v. Jarum ditarik dari mata lalu luka ditutup sementara dengan *cotton bud* yang telah diberi betadine

2) Apabila pada pemeriksaan cairan bola mata tidak ditemukan agen penyebab peradangan pada mata Bapak/Ibu/Saudara, maka Bapak/Ibu/Saudara termasuk dalam kategori uveitis idiopatik (peradangan pada mata yang tidak diketahui penyebabnya), sehingga langkah selanjutnya adalah pemeriksaan IGRA (pemeriksaan yang menilai adanya paparan dengan kuman tuberkulosis) dengan langkah sebagai berikut:

i. Bapak/Ibu/Saudara dalam posisi duduk senyaman mungkin, dengan lengan yang direntangkan dan bersandar pada permukaan yang rata.

ii. Petugas mencuci tangan terlebih dahulu menggunakan air dan sabun atau cairan alkohol, lalu mengenakan sarung tangan.

iii. Petugas memasangkan alat pengikat 10 cm di atas area siku (area pengambilan darah) lalu Bapak/Ibu/Saudara diminta untuk mengepalkan tangan guna memperbesar/memperjelas pembuluh darah.

iv. Petugas mengusapkan alkohol usap 70% pada area pengambilan darah lalu ditunggu hingga kering.

v. Petugas menusukkan jarum ke pembuluh darah balik di siku lalu melakukan pengambilan darah sebanyak 5 ml.

3) Setelah 1-2 minggu kemudian, bila hasil IGRA Bapak/Ibu/Saudara positif, Bapak/Ibu/Saudara akan kembali diambil sampel darahnya oleh petugas yang sudah terbiasa mengambil darah untuk mengetahui penanda peradangan pada tubuh dengan langkah-langkah sebagai berikut:

i. Bapak/Ibu/Saudara dalam posisi duduk senyaman mungkin, dengan lengan yang direntangkan dan bersandar pada permukaan yang rata.

ii. Petugas mencuci tangan terlebih dahulu menggunakan air dan sabun atau cairan alkohol, lalu mengenakan sarung tangan.

iii. Petugas memasangkan alat pengikat 10 cm di atas area siku (area pengambilan darah) lalu Bapak/Ibu/Saudara diminta untuk mengepalkan tangan guna memperbesar/memperjelas pembuluh darah.

iv. Petugas mengusapkan alkohol usap 70% pada area pengambilan darah lalu ditunggu hingga kering.

v. Petugas menusukkan jarum ke pembuluh darah balik di siku lalu melakukan pengambilan darah sebanyak 20 ml.

4) Bapak/Ibu/Saudara akan secara acak dimasukan ke dalam satu dari dua kelompok yang mendapat pengobatan berbeda, namun terapi standar tetes mata anti radang tetap diberikan kepada semua peserta.

a. Pada kelompok pertama, kami akan berikan pengantar ke bagian Penyakit Dalam RSCM untuk dinilai apakah obat anti tuberkulosis (OAT) dapat diberikan, dan dosisnya dihitung sesuai dengan berat badan Bapak/Ibu/Saudara. OAT akan diberikan untuk diminum setiap hari selama 6 bulan. Bapak/Ibu/Saudara akan tetap menjalani kontrol pemeriksaan mata ke RSCM Kirana secara berkala.

b. Pada kelompok kedua, Bapak/Ibu/Saudara akan diberikan obat anti radang (metilprednosolon) yang dosisnya disesuaikan dengan berat badan Bapak/Ibu/Saudara. Pemberiannya akan dipantau dan dievaluasi secara berkala.

5) Bapak/Ibu/Saudara, baik yang mendapat terapi OAT maupun metilprednosolon, akan kembali diambil sampel darahnya pada minggu ke-2 dan bulan ke-6 setelah pengobatan dimulai. Prosedur pengambilan sampel darah sama seperti yang tertera pada poin 3 di atas.

4b. Prosedur alternatif

Tidak ada paksaan kepada Bapak/Ibu/Saudara dalam mengikuti penelitian ini. Bapak/Ibu/Saudara dapat memutuskan untuk tidak ikut serta dalam penelitian ini dan tetap akan mendapatkan terapi tetes mata antiradang dan obat steroid/penekan imun sesuai dengan standar tatalaksana uveitis idiopatik (peradangan pada mata yang belum diketahui penyebabnya) yang berlaku saat ini.

5. Risiko prosedur penelitian

Terdapat kemungkinan terjadinya infeksi pada area pengambilan darah, namun hal ini kami minimalisasi dengan menggunakan alkohol usap dan menjaga kebersihan tangan serta lingkungan ketika dilakukan prosedur pengambilan darah. Rasa nyeri dan pegal mungkin terjadi, namun umumnya hanya berlangsung selama satu hari.

Sampai saat ini, terapi uveitis dengan penyebab yang belum diketahui adalah pemberian obat anti radang tetes mata. Obat steroid/anti radang oral (metilprednisolon) hanya diberikan pada kondisi-kondisi tertentu saja apabila dinilai perlu oleh dokter mata yang merawat. Efek samping pemberian steroid oral meliputi peningkatan tekanan bola mata, gangguan metabolisme gula darah, hingga menurunnya sistem imun tubuh apabila dikonsumsi dalam jangka waktu lama. Namun dalam penelitian ini dosis steroid oral yang diberikan sesuai dengan dosis anjuran yang berlaku, lalu akan diturunkan secara bertahap dan tentunya tetap dalam pengawasan dokter. Bapak/Ibu/Saudara akan mendapatkan jadwal kontrol rutin satu bulan sekali untuk pemantauan respon pengobatan serta efek samping obat yang mungkin terjadi, seperti pemeriksaan berat badan, tekanan darah, gula darah, dan tekanan bola mata di samping pemeriksaan rutin pada organ mata itu sendiri. Apabila terjadi efek samping dari penggunaan obat steroid, maka dosis obat dapat disesuaikan kembali atau bahkan dihentikan sama sekali. Dengan demikian, pemberian steroid oral ini dapat dikatakan relatif aman.

Sedangkan obat anti tuberkulosis (OAT) hingga saat ini belum menjadi terapi standar pada uveitis yang belum diketahui penyebabnya. Efek samping dari pemberian OAT antara lain mual, gangguan penglihatan, gangguan pendengaran, gangguan fungsi hati, kesemutan, hingga reaksi alergi. Namun semua efek samping ini akan berusaha kami cegah sejak awal dengan cara menyesuaikan dosis dengan berat badan Bapak/Ibu/Saudara. Selain itu Bapak/Ibu/Saudara akan mendapat jadwal kontrol rutin ke Departemen Penyakit Dalam yaitu pada akhir bulan ke 2, 5, dan 6 untuk memantau apabila terdapat efek samping dari penggunaan OAT. Apabila terjadi efek samping yang tidak diinginkan maka rejimen OAT dapat disesuaikan kembali atau bahkan dihentikan sama sekali. Dengan demikian, pemberian OAT ini juga dapat dikatakan relatif aman. Apabila dalam perjalanan pengobatan terjadi komplikasi atau kejadian yang tidak diharapkan, Bapak/Ibu/Saudara akan mendapat penanganan secara menyeluruh sesuai dengan prosedur yang berlaku di RSCM. Pada kondisi reaksi obat berat, pemberian OAT dapat dihentikan sewaktu-waktu.

6. Manfaat

Bapak/Ibu/Saudara akan mendapatkan terapi OAT atau steroid anti radang oral untuk penyakit uveitis yang saat ini sedang Bapak/Ibu/Saudara alami. Selain itu terkait dengan kondisi pandemi yang saat ini sedang berlangsung, diberlakukan pembatasan jumlah pasien untuk kontrol ke Poliklinik RSCM, namun dengan berpartisipasi dalam penelitian ini Bapak/Ibu/Saudara akan mendapatkan kuota tambahan pada setiap kesempatan kontrol ke Poliklinik Mata RSCM. Bapak/Ibu/Saudara tidak akan dipungut biaya apapun terkait pemeriksaan/terapi yang menjadi ranah dalam penelitian ini.

7. Kompensasi

Bapak/Ibu/Saudara akan mendapatkan uang ganti transportasi sebesar Rp 75.000 per 1 kali kedatangan kontrol lanjutan yang diperlukan untuk pemeriksaan terkait penelitian.

8. Pembiayaan

Untuk diketahui Bapak/Ibu/Saudara, biaya pemeriksaan laboratorium darah yang menjadi ranah dalam penelitian ini akan ditanggung sepenuhnya oleh peneliti, sedangkan pembiayaan untuk obat-obatan ditanggung oleh BPJS.

9. Kerahasiaan

Semua data dalam rekam medis Bapak/lbu/Saudara akan kami rahasiakan. Selain itu, kami juga meminta kesediaan Bapak/Ibu/Saudara agar darah yang telah diambil dapat kami simpan dalam waktu yang panjang pada penyimpanan khusus yang bertujuan untuk analisis/penelitian di kemudian hari.

10. Kewajiban subyek penelitian

Sebagai subyek penelitian, Bapak/Ibu/Saudara berkewajiban mengikuti aturan atau petunjuk penelitian seperti yang tertulis di atas. Bila ada yang belum jelas, Bapak/Ibu/Saudara bisa bertanya lebih lanjut kepada tim peneliti.

11. Hak untuk menolak/mengundurkan diri

Bapak/lbu/Saudara berhak untuk menolak atau mengundurkan diri dari penelitian ini apabila merasakan sesuatu yang dianggap merugikan. Jika Bapak/Ibu/Saudara menolak untuk berpartisipasi atau mundur dari penelitian ini, keputusan tersebut tidak akan mempengaruhi hubungan Bapak/Ibu/Saudara dengan saya dan tidak akan berdampak pada pelayanan yang berlaku di rumah sakit ini. Bila terjadi hal-hal yang tidak diinginkan atau keluhan lain, Bapak/lbu boleh datang kontrol kapan saja.

Untuk informasi lebih lanjut, silakan hubungi:

dr. Rina La Distia Nora, Sp.M (K), PhD
Departemen Ilmu Kesehatan Mata Fakultas Kedokteran Universitas Indonesia
Rumah Sakit Cipto Mangunkusumo, Jakarta
Telp: 0811-198-910

LEMBAR PERSETUJUAN KEIKUTSERTAAN DALAM PENELITIAN

Semua penjelasan tersebut telah disampaikan kepada saya dan semua pertanyaan saya telah dijawab oleh dr. Rina La Distia Nora, SpM(K), PhD dan tim**.** Saya mengerti bahwa bila memerlukan penjelasan, saya dapat menanyakan kepada peneliti.

<table>
<tr><th colspan="2">Sertifikat Persetujuan (Consent)</th></tr>
<tr><td>Saya telah membaca semua penjelasan tentang penelitian ini. Saya telah diberikan kesempatan untuk bertanya dan semua pertanyaan saya telah dijawab dengan jelas. Saya bersedia untuk berpartisipasi pada studi penelitian ini dengan sukarela.

____
Nama subjek/wali

____
Tanda tangan peserta studi

Tanggal__________________________
hari/bulan/tahun</td><td>Saya mengkonfirmasi bahwa peserta telah diberikan kesempatan untuk bertanya mengenai penelitian ini, dan semua pertanyaan telah dijawab dengan benar. Saya mengkonfirmasi bahwa persetujuan telah diberikan dengan sukarela.

____
Nama peneliti/peminta persetujuan

____
Tanda tangan peneliti/peminta persetujuan

Tanggal__________________________
hari/bulan/tahun</td></tr>
</table>

Informasi Peneliti:

Peneliti utama : dr. Rina La Distia Nora, SpM(K), PhD
Departemen Ilmu Kesehatan Mata FKUI- RSCM
Telp: 0811-198-910

Apabila subjek tuna aksara:

Saya telah menyaksikan pembacaan dari lembar persetujuan (*consent*) kepada subjek/partisipan penelitian dengan akurat, dan telah diberikan kesempatan untuk mengajukan pertanyaan. Saya mengkonfirmasi bahwa subjek/partisipan telah memberikan persetujuannya dengan bebas.

Nama saksi ______________________________ DAN Sidik jari subjek penelitian

Tanda tangan saksi ______________________

Tanggal _______________________________

tanggal/bulan/tahun

Attachment 2. Standardized uveitis workup procedure

<table>
<tr>
<th>Initial step

(Routine)</th>
<th colspan="3">Subsequent steps

These consist of workups that were not performed during the initial step and may be selected based on the anatomical subtype of uveitis or combined with the first step if clinical clues are present. A multidisciplinary approach is warranted when systemic involvement is suspected. The third step is performed if no positive findings are obtained from the second step or if the results are inconclusive.</th>
</tr>
<tr>
<th>1st step</th>
<th>Uveitis anatomical subtype/group</th>
<th>2nd step</th>
<th>3rd step</th>
</tr>
<tr>
<td rowspan="5">Routine systemic investigations:

1. Complete blood counts
2. Erythrocyte sedimentation rate or C-reactive protein
3. Tuberculin skin test (TST) or interferon gamma-release assay (IGRA)
4. Syphilis serological tests (VDRL/RPR, TPHA)
5. Chest X-ray
6. Liver and renal functions
7. HIV screening
8. T helper lymphocytes (CD4+ CD8+), if immunodeficiency is suspected or the presence of clinical presentations of cytomegalovirus retinitis

Additional investigations, if required:

9. Ocular fluid (aqueous tap) from the initial visit if infectious uveitis is suspected, as follows:
• Unilateral presentation
• Granulomatous inflammation
• Anterior uveitis with iris atrophy or increased intraocular pressure (IOP)
• Immunocompromised patients
• The presence of retinal</td>
<td>Acute anterior uveitis</td>
<td>• HLA-B27
• IGRA, if not performed</td>
<td>• Aqueous fluid analysis if HLA-B27 is negative
• Complete urine workups if there is a history of conjunctivitis with suspicion of reactive arthritis
• If TINU (tubulointerstitial nephritis & uveitis) is suspected, beta-2 microglobulin from urine sample
• β-2 microglobulin in urine sample and Angiotensin converting enzyme (ACE) for pediatric patients, adolescents, and young adults</td>
</tr>
<tr>
<td>Chronic anterior uveitis</td>
<td>• Angiotensin Converting Enzyme (ACE)
• Chest CT
• IGRA, if not performed</td>
<td>• HLA-B27
• Serology anti PGL1 for leprae suspicion</td>
</tr>
<tr>
<td>Chronic granulomatous uveitis or multifocal choroiditis</td>
<td rowspan="2">• Angiotensin Converting Enzyme (ACE)
• Chest CT
• IGRA, if not performed
• Toxocariasis serology
• Anti Toxoplasma IgG, IgM from peripheral blood and/or PCR from ocular fluid sample</td>
<td>If there are clinical indications only:
• Minor salivary gland biopsy
• Bronchoscopy dan bronchoalveolar lavage
• 18-FDG PET or 67Ga scintigraphy
• Cryptococcus serology
• Serology anti PGL1</td>
</tr>
<tr>
<td>Chronic intermediate uveitis</td>
<td>• Lumbal puncture
• Brain MRI</td>
</tr>
<tr>
<td>Chronic posterior uveitis</td>
<td>• Anti Toxoplasma IgG, IgM from peripheral blood and/or PCR from ocular fluid.
• Angiotensin converting</td>
<td>• Lumbal puncture for cytology
• Brain MRI</td>
</tr>
</table>

focal lesion<br>• The presence of extensive retinitis<br>• No uveitis improvement with previous empiric/standard treatment.<br><br>10. Diagnostic vitrectomy may be performed if malignancy is suspected<br>11. The 2nd step can be directly considered if there is a suspicion of a relevant clinical presentations.		enzyme (ACE)<br>• Chest CT<br>• IGRA, if not performed<br>• ANCA (if there is a suspicion of Wegener's granulomatosis (WG) or granulomatosis with polyangitis (GPA)) [21, 2221, 2221, 2221, 2221, 22]<br>• If clinically suspected: fungal investigations from ocular fluid	
	**Panuveitis**	• Anti Toxoplasma IgG, IgM from peripheral blood and/or PCR from ocular fluid.<br>• Angiotensin converting enzyme (ACE)<br>• Chest CT<br>• IGRA, if not performed	• Lumbal puncture<br>• Brain MRI<br>• HLA-B27
	**Isolated retinal vasculitis**	• IGRA, if not performed<br>• Complement factors<br>• Antinuclear antibody (ANA)<br>• Antiphospholipid antibody<br>• Anti cytoplasmic antineutrophil antibody (ANCA)	• Angiotensin converting enzyme (ACE)<br>• Complete urine workups
	**Severe uveitis recalcitrant to immunosuppr essants**	• IGRA, if not performed<br>• Ocular fluid (aqueous tap/vitrectomy): for malignancy investigations or infection	• Re-vitrectomy (if malignancy is suspected)<br>• Brain MRI<br>• Angiotensin converting enzyme (ACE)
	**Scleritis**	• IGRA<br>• ANCA<br>• Rheumatoid factor /anti-CCP<br>• ANA	• Complete urine workups<br>• HLA-B27<br>• Angiotensin converting enzyme (ACE)<br>• Anti HCV<br>• HbsAg<br>• C3, C4
	**Paediatric cases**	• IGRA, if not performed<br>• ANA<br>• If immunodeficiency is suspected (IgG, IgM) anti Toxoplasma, CMV, HSV. Ocular fluid analysis if indicated.	• Complete urine workups<br>• HLA-B27

UNIVERSITAS INDONESIA

RESEARCH PROTOCOL

Version 2.0

The Effectivity of Anti-Tuberculosis Therapy in Idiopathic Uveitis With Positive *Interferon Gamma Release Assay* (IGRA): A Randomized Clinical Trial

Principal investigator:

dr. Rina La Distia Nora, Sp.M(K), Ph.D

Research team members:

dr. Ikhwanuliman Putera, Sp.M

Prof. Dr. dr. Ratna Sitompul, SpM(K)

Dr. dr. Made Susiyanti, SpM(K)

Dr. dr. Lukman Edwar, SpM(K)

dr. Yulia Aziza, SpM

Mei Riasanti, S.Si

Dr. dr. Aria Kekalih, M.T.I

Luluk Yunaini, S.Si, M.Biomed

Andriansjah, S.Si, M.Biomed, PhD

Dr. Andi Yasmon, S.Pi, M.Biomed

dr. Priscilla Jessica

dr. Ulifna Alfiya Sifyana

Department of Ophthalmology

Faculty of Medicine University of Indonesia

Cipto Mangunkusumo Hospital

Jakarta

2021

TABLE OF CONTENTS (Version 2.0)

**List of abbreviations**	**34**
**Protocol summary**	**35**
**Introduction and rationale**	**35**
**Objective(s)**	**35**
**Study design and population**	**36**
**Intervention**	**36**
**Main study parameters (endpoints)**	**37**
**Nature and extent of the burden and risks**	**38**
**1. Introduction and Study Objectives**	**39**
**2. Study type**	**41**
**3. Study Population**	**41**
**4. Methods**	**44**
**5. Unexpected discoveries**	**46**
**6. Statistical Analysis**	**47**
**7. Recruitment and consent**	**49**
**8. Handling and storage of data and images**	**50**
**9. Handling and storage of human material**	**51**
**10. Publication**	**52**
**11. References**	**52**
**12. Attachments**	**55**

LIST OF ABBREVIATIONS

COTS	The Collaborative Ocular Tuberculosis Study
LTBI	Latent tuberculosis infection
Mtb	*Mycobacterium tuberculosis*
OTB	Ocular Tuberculosis
PCR	Polymerase Chain Reaction
QFT	QuantiFERON-Gold TB
IGRA	Interferon Gamma Release Assay
SUN	The Standardization of Uveitis Nomenclature
TB	Tuberculosis
TST	Tuberculin Skin Test
ATT	Anti-Tubercular Therapy
NEI	National Eye Institute
RCT	Randomized Controlled Trial
MTA	Material Transfer Agreement
PBMC	Peripheral Blood Mononuclear Cells
RNA	Ribonucleic Acid

PROTOCOL SUMMARY

Introduction and Rationale:

Among the infectious causes of uveitis, TB is an etiology that must always be considered. In countries with a high incidence of TB, such as Indonesia, uveitis associated with concurrent active systemic TB accounts for 8% of newly referred uveitis cases in our setting. Interestingly, among patients with uveitis of undetermined cause, some exhibited TB immunoreactivity as detectable through interferon-gamma release assays (IGRA) or tuberculin skin tests (TST). Moreover, the proportion of such patients exceeded the rate of positive TB immunoreactivity in the general population, raising concerns that the presenting uveitis may represent a true manifestation of extrapulmonary TB in the eye.

Before the current trial, multiple retrospective studies reported treatment outcomes in the management of TB uveitis. There is no gold standard for diagnosing TB uveitis, partly due to the suboptimal diagnostic performance of current ocular fluid polymerase chain reaction (PCR) tests for detecting *Mycobacterium tuberculosis* (*Mtb*) genomes in ocular fluid samples. Most studies evaluating treatment outcome included both uveitis patients with proven active systemic TB and those showing TB immunoreactivity after other causes had been excluded. As previously summarized, treatment with antitubercular therapy (ATT) in these patients demonstrated an overall trend toward a better resolution of uveitis but failed to achieve statistical significance. Cautious interpretation is warranted, as our retrospective analysis suggests that the severity of presenting uveitis might influence treatment decisions regarding the initiation of ATT, complicating the interpretation of the actual benefits of ATT in this context. Of note, based on the opinions of uveitis experts, in the absence of active systemic TB, there is no strong consensus on initiating ATT unless ophthalmologic presentations such as choroidal granuloma or serpiginous-like choroiditis are observed.

Objective:

To determine whether ATT would eventually improve outcomes compared to treatment without ATT in terms of uveitis resolution and relapse, during subsequent follow-up of patients with uveitis of undetermined cause who were showing TB immunoreactivity, based on QuantiFERON-TB Gold Plus (QFT) test positivity.

Study design & study population:

Single blind randomized controlled trial (RCT) – single center

All newly referred uveitis patients will be evaluated for eligibility at the outpatient uveitis clinic, Department of Ophthalmology, Faculty of Medicine, Universitas Indonesia - Cipto Mangunkusumo Hospital, Jakarta, Indonesia.

Inclusion criteria are: (1) adults (age ≥18 years) with newly diagnosed, clinically active uveitis of undetermined cause, following standardized workups at our center (see Supplementary file); (2) positive QFT test result (>0.35 U/mL; Kimia Farma Laboratory, Depok, PT. Kimia Farma Diagnostika); (3) local residence in Jakarta, Bogor, Depok, Tangerang, or Bekasi; and (4) consent to participate in the study through the entire monitoring period. Exclusion criteria were: (1) confirmed active systemic TB or living with an active TB patient; (2) HIV-positive status; (3) visual acuity less than hand movement with signs of phthisis on ophthalmological examinations and ultrasound; (4) impaired liver function or other systemic conditions, as assessed by the internist-pulmonologist, that precluded eligibility for ATT; (5) pregnancy, and (6) recent antibiotic use, including ATT, or systemic steroid use exceeding 8 mg prednisone equivalent daily within the last 14 days.

Ocular PCR will be performed in selected cases based on the clinical judgement of the attending uveitis specialist. This will be done (1) to confirm the clinical diagnosis, or (2) to exclude other differential diagnosis. If it is performed, any positive results will result in exclusion from trial entry.

After eligibility assessment, participants will be randomly assigned in a 1:1 ratio to one of the two treatment groups without stratification factors. Randomization will be conducted by a dedicated research manager using block randomization (block size of 4) to ensure equal distribution between the groups.

Intervention:

All included participants will receive immunosuppressants at the discretion of the attending uveitis specialists. There will be no restrictions on topical or local treatments throughout the entire follow-up period, including surgical interventions, if indicated.

- *Arm 1: ATT*

The full course of ATT will consist two months in the intensive phase, followed by seven months in the continuation phase (2RHZE/7RH).

- *Arm 2: Control group*

The treatment for the control group primarily involved immunosuppressants without ATT.

Main study parameters/endpoints:

Primary Outcome

- Complete resolution of uveitis at the patient level at six months.

Criteria for complete uveitis resolution (applied to both eyes: patient-level outcome): (1) fewer than or equal to 0.5+ anterior chamber cells according to the Standardized Uveitis Nomenclature (SUN) grading system, fewer than or equal to 0.5+ vitreous haze based on clinical grading using the NEI (National Eye Institute) scale, and no active retinal or choroidal lesions; and (2) no more than 7.5 mg of oral prednisone daily (i.e., methylprednisolone at 6 mg or less daily) and fewer than or equal to 2 drops of prednisolone acetate 1% (or equivalent) per day. These criteria have to be maintained for at least 90 days following the visit when the first complete uveitis resolution was recorded.

If there is insufficient follow-up after resolution, maintained uveitis resolution is still considered. Participants who meet criterion 1 but not criterion 2 will be classified as having partial uveitis resolution, and for the primary endpoint classification, they will be considered to have non-complete uveitis resolution.

Secondary outcome

- The details of treatment failure (partial resolution or non-responsive)
- Time to uveitis resolution
- Uveitis relapse following complete resolution
- Ocular-level outcomes: visual acuity changes and secondary glaucoma

Nature and extent of the burden and risks associated with participation, benefit and group relatedness:

Adverse events

Adverse events will be recorded during the trial. Baseline consultation and workup include assessment of potential adverse events risks. Participants will be closely monitored by the internal medicine department. Participants can withdraw their consent at any time during the trial.

1. INTRODUCTION AND STUDY OBJECTIVES

1.1 Introduction

Uveitis is an umbrella term for intraocular inflammation caused by infectious or non-infectious causes[1, 2]. Currently, more than 30 diseases associated with uveitis have been identified[1, 2]. However, more than one-third of clinical uveitis cases lack definitive etiologies, falling under the category of "uveitis of undetermined cause" or idiopathic[2, 3]. In clinical practice, uveitis is considered infectious until proven otherwise[4, 5]. Therefore, systematic investigations using various diagnostic tests, along with multidisciplinary consultations are often required[6, 7]. In cases of non-infectious uveitis, immunosuppressants—starting with steroids administered locally, systemically, or both—is the mainstay of treatment[5]. For infectious uveitis, appropriate antimicrobial treatment is essential for achieving uveitis resolution[2]; otherwise, the condition maintains and may worsens even into blindness such as in tubercular (TB) uveitis [8].

Among the infectious causes of uveitis, TB is an etiology that must always be considered. In countries with a high incidence of TB, such as Indonesia, uveitis associated with concurrent active systemic TB accounts for 8% of newly referred uveitis cases in our setting[9]. Interestingly, among patients with uveitis of undetermined cause, some exhibited TB immunoreactivity as detectable through interferon-gamma release assays (IGRA) or tuberculin skin tests (TST). Moreover, the proportion of such patients exceeded the rate of positive TB immunoreactivity in the general population[9, 10], raising concerns that the presenting uveitis may represent a true manifestation of extrapulmonary TB in the eye.

Before the current trial, multiple retrospective studies reported treatment outcomes in the management of TB uveitis[11]. There is no gold standard for diagnosing TB uveitis[12, 13], partly due to the suboptimal diagnostic performance of current ocular fluid polymerase chain reaction (PCR) tests for detecting *Mycobacterium tuberculosis* (Mtb) genomes in ocular fluid samples[7, 14]. Most studies evaluating treatment outcome included both uveitis patients with proven active systemic TB and those showing TB immunoreactivity after other causes had been excluded. As previously summarized, treatment with antitubercular therapy (ATT) in these patients demonstrated an overall trend toward a better resolution of uveitis but failed to achieve statistical significance[11, 15]. Cautious interpretation is warranted, as our

retrospective analysis suggests that the severity of presenting uveitis might influence treatment decisions regarding the initiation of ATT, complicating the interpretation of the actual benefits of ATT in this context [16]. Of note, based on the opinions of uveitis experts, in the absence of active systemic TB, there is no strong consensus on initiating ATT unless ophthalmologic presentations such as choroidal granuloma or serpiginous-like choroiditis are observed[13].

In light of this, we conducted a randomized clinical trial to determine whether ATT would eventually improve outcomes compared to treatment without ATT in terms of uveitis resolution and relapse, during subsequent follow-up of patients with uveitis of undetermined cause who were showing TB immunoreactivity, based on QuantiFERON-TB Gold Plus (QFT) test positivity.

1.2. Objective (s)

Primary objective

To compare the clinical effectivity of initial treatment approach that include ATT in addition to immunosuppressants versus immunosuppressants alone in undetermined cause of uveitis cases with positive IGRA test.

Secondary objectives

a) To explore details of treatment details of treatment failure (partial resolution or non-responsive), time to uveitis resolution, uveitis relapse following complete resolution, and eye-level outcomes, which consisted of the differences in visual acuity between baseline and month 6, as well as the incidence of secondary glaucoma.
b) To explore relevant biomarkers at baseline, during, and at the end of primary end point (6 months follow-up).

1.3. Hypothesis

Patients that received initial treatment incorporating a full course of ATT in QFT-positive patients with uveitis of undetermined cause is achieving complete uveitis resolution at six months and reducing uveitis relapse during follow-up after the initial six months.

2. Study type

2.1 Study type:

☐ Retrospective

☒ Prospective

☐ Combination Retrospective/Prospective

2.2 Check all the applicable boxes:

☐ Medical records (re-use of data from healthcare, including AI)

☐ Case report

☐ Re-use data from research

☐ Evaluations of quality of healthcare (retrospective)

☐ Research with additional use of residual material from regular healthcare

☐ Research with re-use of human materials from research or existing biobank

☐ Research with human materials without biobank

☐ De novo biobank (human material obtained without burdensome or invasive procedures)

☒ Interventional

☐ Phase IV research

☐ Healthcare evaluation research (prospective)

☐ Medical devices

☐ In Vitro Diagnostic Tests

☐ Other research, *describe*

3. Study population

a. Population (base)

Patients diagnosed with Uveitis of undetermined cause with positive TB immunoreactivity (IGRA) at the outpatient clinics in Dr. Cipto Mangunkusumo National Central Public Hospital, Jakarta.

b. Inclusion criteria:

5. Patient that is newly diagnosed with uveitis of unknown origin/idiopathic (as evidenced by a series of uveitis work-up tests) and tested positive for IGRA (>0.35 U/ml).
6. Age >18 years old.
7. Lives in Jakarta/Bogor/Depok/Tangerang/Bekasi area or willing to participate in research until the end of monitoring program.
8. Willing to participate in the research and sign the informed consent after receiving explanation regarding the research.

c. Exclusion criteria:

13. Patients with positive aqueous tap examination results on one of the examination panels for the bacteria causing infectious uveitis according to the standard examination.
14. Anterior uveitis patient with a positive HLA-B27 test result.
15. The patient is proven to have active TB or lives in the same house with an active TB patient.
16. Patients are included in the TB reactivation risk index group according to the 2018 WHO LTBI (Latent Tuberculosis Incident) Guideline.
17. HIV positive patient.
18. Patients with inactive uveitis at the first visit.
19. Patients with visual acuity less than 1/300 or showing signs of preptisis based on ophthalmological examination and ultrasound of the eye.
20. The patient has a history of previous ATT consumption.
21. Patients with impaired liver function or other systemic conditions which according to the Internal Medicine Department are not eligible to receive ATT.
22. The patient has a history of taking antibiotics in the last 1-2 weeks.
23. The patient is not willing to sign the informed consent.
24. The patient was pregnant at the first visit or was planning to become pregnant during the study period.

d. Sample size calculation

The sample size is calculated using a formula of two-independent proportion sample size calculation. The primary end point of uveitis resolution at 6 month is used for this sample size calculation.

Based on the Multicenter Uveitis Steroid Treatment (MUST) trial in non-infectious uveitis, the proportion of uveitis patients with active disease at six months following treatment was found to be 44%[17]. A 30% difference in uveitis resolution between the two groups is predetermined. Using a Zα of 1.96 and a Zβ of 0.84 (for a two-sided significance level of 5% and 80% power), the estimated sample size required for each treatment group will be 35 participants. With 10% increase to anticipate dropout, a total of 39-40 participants in each treatment group is required.

4. $$N1 = N2 = \left(\frac{Z\alpha\sqrt{2PQ}+Z\beta\,\sqrt{P1Q1+P2Q2}}{P1-P2}\right)^2$$

Zα (1.96), power (80%, Zβ = 0.84), P1 based on MUST trial 44% (Q2 = 56%). Estimated P1 – P2 = 30%. Thus, P2 = 14% (Q2 = 86%). As 10% drop out rate is being anticipated, N1 = N2 = 39 ~ 40 (total 78-80 participants)

g. (Planned) start date

Date start collection data: August 2021 (tentative after ethical and legal approval)

h. (Planned) end data

Date end collection data: February 2024

4. Methods

4.1 *Please describe how you do conduct research and which methods are used, e.g.*

This single-blinded, randomized controlled trial was conducted at Cipto Mangunkusumo Hospital, a tertiary eye hospital in Jakarta, Indonesia.

All newly referred uveitis patients will be evaluated for eligibility at the outpatient uveitis clinic, Department of Ophthalmology, Faculty of Medicine, Universitas Indonesia - Cipto Mangunkusumo Hospital, Jakarta, Indonesia.

Eligible participants will undergo standardized workups and received consultations from the participating internist-pulmonologist at our hospital. The internist-pulmonologist will be unaware of the randomization and provided all patients with the necessary information and counseling regarding ATT prescription and initiation.

Treatment arms

**Study arms**	**Intervention**	**Control**
**Anti-Tuberculosis Therapy**	Yes	No
	Dosage form: ATT fixed-dose combination (FDC). FDC intensive phase containing 150 mg rifampicin, 75 mg isoniazid, 400 mg pyrazinamide, and 275 mg ethambutol), while FDC continuation phase containing rifampicin-isoniazid. Dosage: according to body weight, 30-37 kg: 2 tablets, 38-54 kg: 3 tablets, 55-70 kg: 4 tablets, more than 70 kg: 5 tablets. Frequency: Intensive phase: once daily. Continuation phase: 3 times/week. Duration: 9 months (2 months of FDC intensive phase + 7 months of FDC continuation phase). The duration will be standardized for all included participants, with	-

	exceptions for those requiring ATT adjustments due to other factors (e.g., adverse effects, allergies, etc.).	
**Immunosuppressants**	Possible (Depending on the clinical presentation and the clinical judgment of the blinded attending uveitis specialist)	Yes
	Oral methylprednisolone at a dosage of 0.8 mg/kg of body weight per day (maximum of 56 mg/day), which will be tapered gradually based on the intraocular inflammation observed. This dose is equivalent to 1 mg/kgBW/day (max 60 mg/day).<br>Tapering of oral methylprednisolone involve reducing the dose by 8 mg for doses above 20 mg and by 4 mg for doses below 20 mg.<br>All patients receiving systemic steroids, including those in the ATT group, will also receive vitamin D, calcium supplements, and gastric protectors (e.g., omeprazole or antacids).<br>Local steroids (e.g., steroid drops) will be prescribed based on the clinical presentation.<br>Disease-modifying anti-rheumatic drugs (DMARDs) may be prescribed based on the clinical judgment of the attending pulmonologist/internist-immunologist.	

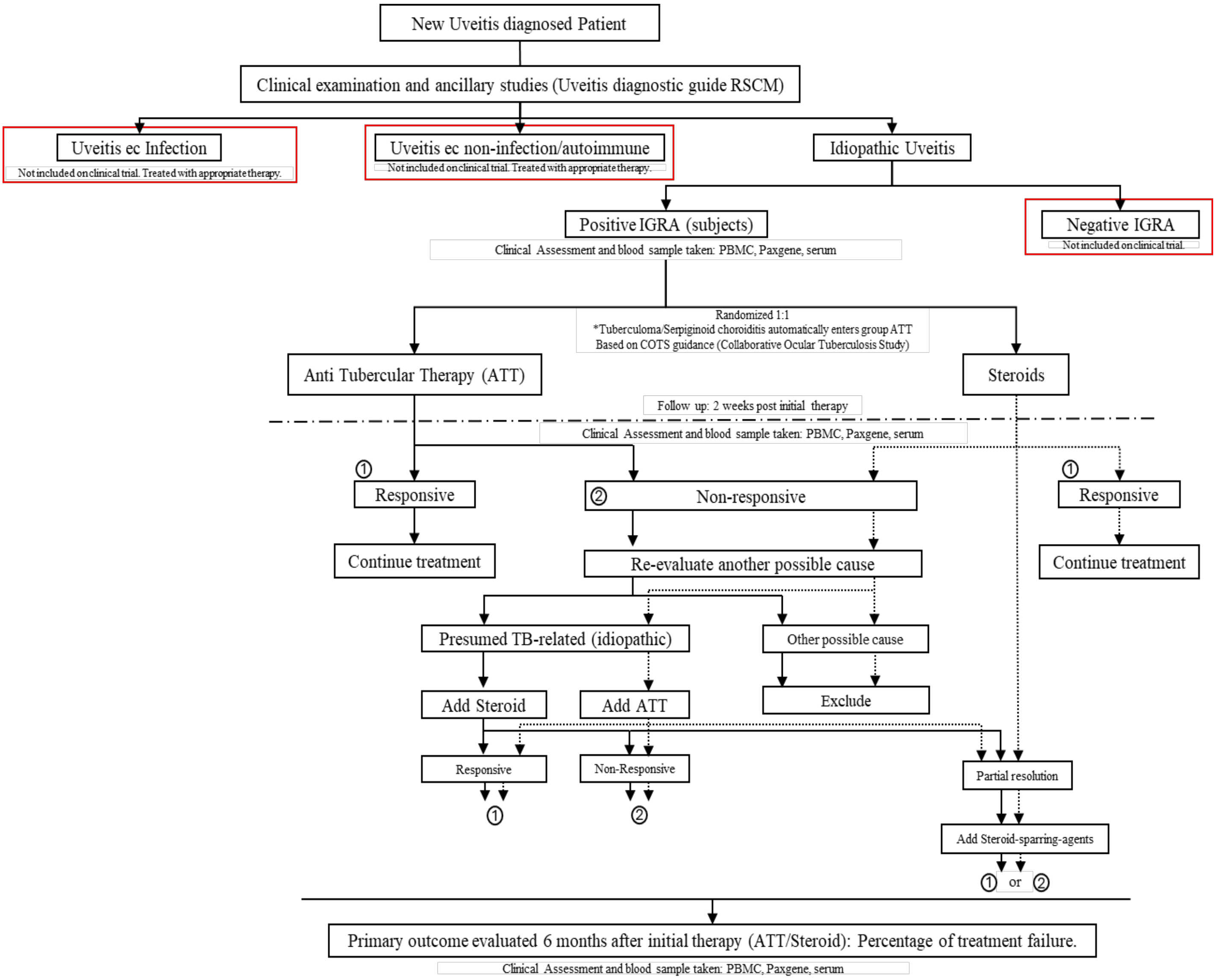

5. Unexpected discoveries

- Is there a chance of unexpected discoveries?

  ☐ Yes

  ☒ No

- If yes, describe the procedures, who will be notified, how the subjects are notified.

6. Statistical analysis

- The intention-to-treat (ITT) approach

All analyses will be first conducted using the ITT approach. The ITT population will include all participants who underwent randomization, regardless of any treatment switching that occurred. For participants who missed the six-month visit (±28 days), if any, last observation carried forward (LOCF) imputation will be performed.

- The per-protocol approach

The per-protocol population will include all participants who were randomized, excluding those who do not have six-month visits or deviated from the treatment protocols (e.g., non-compliant, experience severe adverse effect, etc). If any, participants who switch treatment will also be excluded in this per-protocol analysis.

For patients receiving ATT, missing 2 weeks of medication will be considered non-compliance (protocol deviation). The pulmonologist will record the prescribed ATT and count the remaining drugs (i.e., ATT) at each visit to determine treatment non-compliance. For participants receiving systemic immunosuppressants, missing more than 1 week of the prescribed regimen will be considered non-compliance. Topical immunosuppressants will not be taken into account when determining compliance.

- Additional approach for potential switching

In order to anticipate treatment switch, adjustment will be performed, particularly to analyze the secondary outcomes. Participants grouping will be subdivided into those receiving combination treatment besides the initial group of ATT alone and non-ATT. Specifically, participants that switch from non-ATT group to ATT will be classified as ATT in this adjusted analysis. Analysis following adjustment for switching treatment was performed with recalculation of the six-month follow-up time based on the actual treatment received, beginning from the start of their actual treatment.

However, results from the ITT analysis (without adjustment for switching) is considered to be more appropriate to evaluate the primary outcome, given that the purpose of the study was to compare the two strategies according to the treatment received at the time of randomization.

6.1. Main study parameter/endpoint:

Response to treatment

At 6 month follow-up:

Criteria for complete uveitis resolution applied to both eyes, in cases with bilateral uveitis, included[18]: (1) fewer than or equal to 0.5+ anterior chamber cells according to the Standardized Uveitis Nomenclature (SUN) grading system, fewer than or equal to 0.5+ vitreous haze based on clinical grading using the NEI (National Eye Institute) scale, and no active retinal or choroidal lesions; and (2) no more than 7.5 mg of oral prednisone daily (i.e., methylprednisolone at 6 mg or less daily) and fewer than or equal to 2 drops of prednisolone acetate 1% (or equivalent) per day. Treatment will be categorized as a failure if subject did not meet the following criteria at the 6-month follow-up.

6.2. Additional study parameters (endpoints)

a. Time to uveitis resolution: Calculated from the time of randomization. These criteria had to be maintained for at least 90 days following the visit when the first complete uveitis resolution was recorded. If there was insufficient follow-up after resolution, maintained uveitis resolution was still considered. Participants who met criterion 1 but not criterion 2 were classified as having partial uveitis resolution (non-complete uveitis resolution)
b. Uveitis relapse (subsequent follow-up, beyond 6 months). Uveitis relapse is defined as any worsening of ocular inflammation (including a two-step increase in anterior chamber or vitreous cells as per the SUN grading system) or the occurrence of clinically new inflammatory activity (such as choroidal or retinal lesions) that necessitated a modification of local or systemic uveitis treatment after a minimum of 90 days of complete uveitis resolution at the patient level[19, 20].
c. Time to relapse was calculated from the first visit showing inactive uveitis until the first notification of uveitis relapse.

d. Biomarkers associated with trial from samples obtained from study participants. Exploratory investigations may be conducted as supplementary analyses. Samples will be collected and stored following the institution's standardized procedures for biobanking of human study materials. Details of further analyses will be provided in a separate document.

e. Secondary glaucoma: the occurrence of increased intraocular pressure (IOP) above 21 mmHg in at least two consecutive visits that necessitated prescription of IOP-lowering medications.

f. Visual acuity: Visual acuity was considered to increase if the difference in best-corrected visual acuity between month 6 and baseline visits (measured with Snellen, in decimal equivalent) was equal to or greater than 0.1, stable if within the range of -0.1 to 0.1, and decreased if less than -0.1.

(If any cataract or vitreoretinal surgery was performed before the six-month follow-up, the last recorded visual acuity prior to the surgery was used).

7 Recruitment and consent

7.1 Will the subjects be asked for informed consent?

☒ Yes *(Upload Patient Information Letter and Informed Consent)*

☐ No, only anonymous data is used, i.e. the data can never be traced back to an individual subject

☐ No, this research will be performed under the exception consent *(Upload form Care for data Template)*

☐ Other (e.g. partly, indirectly) *Please describe the situation.*

7.1 *If yes, please give a description of the recruitment and informed consent procedures. How and by whom (investigator, supervising doctor, other person) participants will be informed about the study and asked for their consent, how much time will they be given to consider the decision.The patient information letter with informed consent form should be attached as a separate document.*

The principal investigator or a designated team member responsible for participant recruitment will obtain informed consent directly and in person. Participants, or their legal guardian if applicable, must provide consent before any intervention associated with the trial is administered. Participants have the right to ask questions, accept, or refuse any intervention related to the trial. Additionally, they have the right to withdraw consent at any time.

a. If no, exception consent: *describe how it is safeguarded that subjects are excluded who have objected against the re-use of their data, human material, images.*

Not applicable

8. Handling and storage of data and images

8.1 *Describe how subject's privacy is protected. Describe how, when and by whom data is coded, and how the key table is safeguarded.*

Data will be collected using a designated research form and entered into an electronic database. There will be two databases: The first database will contain a unique ID linked to a particular participant's data in the trial. The second database will include a newly generated ID and data relevant to the trial, without any patient names, initials, or medical record numbers. Anonymity will be preserved, and subject identification will be based on a specific code assigned to each participant. The master dataset, which is password-protected and links specific codes to individual patients, will only be accessible to selected investigators.

8.2 *Describe how data is stored (i.e. which data management system/data capture system), who has access to the coded source data, how long data will be kept, which steps are taken to ensure data security, what happens with the data after the research has been completed.*

The data from the medical records will be manually input and stored into electronic data capture (CASTOR) with a pre-set database. Each investigator will have a specific role to view, edit, and validate the data. The data will only be accessed for this research purpose for this specific study duration. All investigators guarantee that the entered data will not be sent or used outside this study purpose. Once this study completed, any further analysis with the data should obtain a new permission.

8.3 *Describe how images are stored, how the subject's privacy is protected, what happens with images after the research has been completed.*

All data, including images, will be stored in CASTOR during this study period. All data will be stored without patient name, medical records number, or any identifiable clues toward the particular patient.

9. Handling and storage of human material

IGRA testing will be performed as part of the study, separate from the routine procedures conducted by the hospital. Blood collection for this test will be combined with the collection of participant samples for biobanking and biomarker discovery.

Blood Sample Collection: Conducted at the Department of Ophthalmology, Faculty of Medicine University of Indonesia, by trained nurses/staff.

- Procedure:
  - The patient sits with the arm extended.
  - The staff washes hands and wears non-sterile gloves.
  - Identifies a clearly visible vein.
  - A tourniquet is applied 10 cm above the phlebotomy site.
  - Asepsis is performed using 70% alcohol swab and allowed to dry.
  - A needle is inserted at a 15-30° angle until blood flashback is observed.
  - A 20 mL blood sample is collected.

- Sample Storage and Processing:

Samples for biobanking will be stored as PBMC, serum, and whole blood (PAXgene) in the laboratory.

- PAXgene samples are stored at low temperatures per protocol.
- PBMC is isolated using Ficoll gradient in a Biosafety Level II Cabinet and stored in liquid nitrogen.

- RNA Isolation and Genetic Analysis:
- PBMC is thawed in 37°C water (<5 minutes) before further processing.
- RNA is extracted from PBMC and whole blood using an RNA Extraction Kit.

When the vacutainer arrives at the processing laboratory, it will be processed immediately. The aliquot sample will be stored at 4°C during processing and transferred to ultra-low temperature long-term storage using liquid nitrogen as soon as possible after processing to ensure that all samples are preserved in cryopreservation. Any subsequent material transfer will follow the Material Transfer Agreement (MTA) procedure under national regulations (Ministry of Health of the Republic of Indonesia No. 657/MENKES/PER/VIII/2009).

10. Publication

Do you have the intention to submit the study results in a manuscript for publication in a journal:

☒ Yes

☐ No, *please motivate*

11. References

1. Burkholder BM, Jabs DA. Uveitis for the non-ophthalmologist. *Bmj* 2021; **372:** m4979.

2. Jabs DA, Busingye J. Approach to the diagnosis of the uveitides. *Am J Ophthalmol* 2013; **156**(2)**:** 228-236.

3. Tsirouki T, Dastiridou A, Symeonidis C, Tounakaki O, Brazitikou I, Kalogeropoulos C, Androudi S. A Focus on the Epidemiology of Uveitis. *Ocul Immunol Inflamm* 2018; **26**(1)**:** 2-16.

4. Agrawal R, Thng ZX, Gupta A, Toy BC, Dick AD, Smith JR *et al.* Infectious Uveitis: Conversations with the Experts. *Ocul Immunol Inflamm* 2023; **31**(7)**:** 1333-1341.

5. Writers AM. Manage non-infectious uveitis in adults with individualized immunosuppressive therapy and regular monitoring. *Drugs & Therapy Perspectives* 2016; **32**(6)**:** 244-248.

6. Sève P, Cacoub P, Bodaghi B, Trad S, Sellam J, Bellocq D *et al.* Uveitis: Diagnostic work-up. A literature review and recommendations from an expert committee. *Autoimmunity Reviews* 2017; **16**(12)**:** 1254-1264.

7. Ludi Z, Sule AA, Samy RP, Putera I, Schrijver B, Hutchinson PE *et al.* Diagnosis and biomarkers for ocular tuberculosis: From the present into the future. *Theranostics* 2023; **13**(7)**:** 2088-2113.

8. Basu S, Das T. Pitfalls in the management of TB-associated uveitis. *Eye (Lond)* 2010; **24**(11)**:** 1681-1684.

9. La Distia Nora R, Sitompul R, Bakker M, Susiyanti M, Edwar L, Sjamsoe S *et al.* Tuberculosis and other causes of uveitis in Indonesia. *Eye (Lond)* 2018; **32**(3)**:** 546-554.

10. Shrestha AB, Siam IS, Tasnim J, Dahal A, Roy P, Neupane S *et al.* Prevalence of latent tuberculosis infection in Asian nations: A systematic review and meta-analysis. *Immun Inflamm Dis* 2024; **12**(2)**:** e1200.

11. Betzler BK, Putera I, Testi I, La Distia Nora R, Kempen J, Kon OM *et al.* Anti-tubercular therapy in the treatment of tubercular uveitis: A systematic review and meta-analysis. *Surv Ophthalmol* 2023; **68**(2)**:** 241-256.

12. Agrawal R, Testi I, Mahajan S, Yuen YS, Agarwal A, Rousselot A *et al.* The Collaborative Ocular Tuberculosis Study (COTS) Consensus (CON) Group Meeting Proceedings. *Ocul Immunol Inflamm* 2020; **28**(sup1)**:** 85-95.

13. Agrawal R, Testi I, Bodaghi B, Barisani-Asenbauer T, McCluskey P, Agarwal A *et al.* Collaborative Ocular Tuberculosis Study Consensus Guidelines on the Management of Tubercular Uveitis-Report 2: Guidelines for Initiating Antitubercular Therapy in Anterior Uveitis, Intermediate Uveitis, Panuveitis, and Retinal Vasculitis. *Ophthalmology* 2021; **128**(2)**:** 277-287.

14. Vasconcelos-Santos DV, Zierhut M, Rao NA. Strengths and weaknesses of diagnostic tools for tuberculous uveitis. *Ocul Immunol Inflamm* 2009; **17**(5)**:** 351-355.

15. Putera I, Schrijver B, Ten Berge J, Gupta V, La Distia Nora R, Agrawal R *et al.* The immune response in tubercular uveitis and its implications for treatment: From anti-tubercular treatment to host-directed therapies. *Prog Retin Eye Res* 2023; **95:** 101189.

16. Putera I, Ten Berge J, Thiadens A, Dik WA, Agrawal R, van Hagen PM *et al.* Clinical Features and Predictors of Treatment Outcome in Patients with Ocular Tuberculosis from the Netherlands and Indonesia: The OculaR TB in Low versus High Endemic Countries (ORTEC) Study. *Ocul Immunol Inflamm* 2024**:** 1-12.

17. Multicenter Uveitis Steroid Treatment Trial Research G, Kempen JH, Altaweel MM, Holbrook JT, Jabs DA, Louis TA *et al.* Randomized comparison of systemic anti-inflammatory therapy versus fluocinolone acetonide implant for intermediate, posterior, and panuveitis: the multicenter uveitis steroid treatment trial. *Ophthalmology* 2011; **118**(10)**:** 1916-1926.

18. Rathinam SR, Gonzales JA, Thundikandy R, Kanakath A, Murugan SB, Vedhanayaki R *et al.* Effect of Corticosteroid-Sparing Treatment With Mycophenolate Mofetil vs Methotrexate on Inflammation in Patients With Uveitis: A Randomized Clinical Trial. *Jama* 2019; **322**(10)**:** 936-945.

19. Jabs DA, Nussenblatt RB, Rosenbaum JT, Standardization of Uveitis Nomenclature Working G. Standardization of uveitis nomenclature for reporting clinical data. Results of the First International Workshop. *Am J Ophthalmol* 2005; **140**(3)**:** 509-516.

20. Agrawal R, Agarwal A, Jabs DA, Kee A, Testi I, Mahajan S *et al.* Standardization of Nomenclature for Ocular Tuberculosis - Results of Collaborative Ocular Tuberculosis Study (COTS) Workshop. *Ocul Immunol Inflamm* 2020; **28**(sup1)**:** 74-84.

21. Majumder PD, Sudharshan S, Biswas J. Laboratory support in the diagnosis of uveitis. *Indian journal of ophthalmology* 2013; **61**(6)**:** 269-276.

22. Macarie SS, Kadar A. Eye involvement in ANCA positive vasculitis. *Rom J Ophthalmol* 2020; **64**(1)**:** 3-7.

Attachement 1. Informed consent (*in Bahasa as it was in the original file).*

LEMBAR PENJELASAN KEPADA CALON SUBJEK

Saya, dr. Rina La Distia Nora, SpM(K), PhD dan tim, dari Departemen Ilmu Kesehatan Mata FKUI - RSCM akan melakukan penelitian dengan judul **"Efektivitas obat anti tuberkulosis sebagai terapi uveitis idiopatik IGRA positif: Penelitian Uji Klinis Terandomisasi".**

Saya akan memberikan informasi kepada (Bapak/Ibu/Saudara) mengenai penelitian ini dan mengundang (Bapak/Ibu/Saudara) untuk menjadi bagian dari penelitian ini.

Bapak/lbu/Saudara yang terhomat,

Bapak/lbu/Saudara saat ini menderita uveitis, yaitu peradangan pada bagian dalam mata yang dicurigai terjadi akibat infeksi (virus, bakteri, atau parasit), proses autoimun, atau penyebab yang masih belum dapat ditentukan. Untuk uveitis yang tidak diketahui penyebabnya (idiopatik), kami mengelompokkannya berdasarkan hasil pemeriksaan tes IGRA: positif atau negatif. IGRA adalah pemeriksaan sampel darah untuk mengetahui apakah Bapak/lbu/Saudara pernah terpapar dengan kuman tuberkulosis. Kami ingin menganalisis pengaruh pemberian obat antituberkulosis (OAT) dibandingkan dengan obat steroid (terapi anti radang oral) selama 6 bulan pada Bapak/Ibu/Saudara, yang dinilai dari hilangnya peradangan pada mata.

Bapak/Ibu/Saudara dapat berpartisipasi dalam penelitian ini dengan cara menandatangani formulir ini. Jika Bapak/Ibu/Saudara setuju untuk berpartisipasi dalam penelitian ini, Bapak/Ibu/Saudara kapan saja dapat secara bebas mundur dari penelitian ini. Jika Bapak/Ibu/Saudara menolak untuk berpartisipasi atau mundur dari penelitian ini, keputusan tersebut tidak akan mempengaruhi hubungan Bapak/Ibu/Saudara dengan saya dan tidak akan berdampak pada pelayanan yang berlaku di rumah sakit ini.

Jika Bapak/Ibu/Saudara tidak mengerti tiap pernyataan dalam formulir ini, Bapak/Ibu/Saudara dapat menanyakannya kepada saya.

12. Tujuan penelitian

Penelitian ini bertujuan melihat dampak pemberian obat anti tuberkulosis (OAT) selama enam bulan dibandingkan dengan pemberian steroid (obat anti radang oral) dalam mengobati uveitis yang belum diketahui penyebabnya (idiopatik) namun disertai dengan hasil pemeriksaan IGRA positif (diartikan pernah terpapar dengan kuman tuberkulosis). Di samping itu, penelitian ini juga bertujuan mengetahui pola perjalanan penanda peradangan (yang dianalisis dari sampel darah) pada subjek yang mengalami uveitis idiopatik IGRA positif, mulai dari sebelum hingga selesai menjalani pengobatan.

13. Partisipasi dalam penelitian

Jika Bapak/Ibu/Saudara berkenan diikutsertakan dalam studi ini, Bapak/Ibu/Saudara akan menjalani pemeriksaan mata standar di poliklinik dilanjutkan dengan pengambilan sampel darah untuk pemeriksaan substansi penanda peradangan dalam darah yang berhubungan dengan peradangan pada mata, yang sampai saat ini masih terus diteliti.

Bapak/Ibu/Saudara akan dimasukan ke dalam satu dari dua kelompok yang menerima pengobatan oral yang berbeda secara acak, namun terapi standar tetes mata anti radang tetap diberikan kepada semua peserta. Pada kelompok pertama, kami akan berikan pengantar pemberian obat anti tuberkulosis (OAT) ke bagian Penyakit Dalam RSCM untuk dinilai apakah OAT dapat diberikan, dan dosisnya dihitung sesuai dengan berat badan Bapak/Ibu/Saudara. OAT akan diberikan untuk diminum setiap hari selama 6 bulan sesuai dosis. Bapak/Ibu/Saudara akan tetap menjalani kontrol pemeriksaan mata ke RSCM Kirana secara berkala. Sedangkan pada kelompok kedua, Bapak/Ibu/Saudara akan diberikan obat anti radang metilprednisolon yang dosisnya disesuaikan dengan berat badan Bapak/Ibu/Saudara. Pemberiannya akan dipantau dan dievaluasi secara berkala.

Sebagai catatan, bagi Bapak/Ibu/Saudara yang menerima terapi OAT, apabila selama 2-4 minggu awal terjadi perburukan pada kondisi mata Bapak/Ibu/Saudara, maka Bapak/Ibu/Saudara akan mendapatkan tambahan obat anti radang berupa metilprednisolon oral untuk mengendalikan peradangan. Sebaliknya, bagi Bapak/Ibu/Saudara yang menerima metilprednisolon dan setelah pemantauan dipertimbangkan pemberian OAT, maka Bapak/Ibu/Saudara akan kami pertimbangkan untuk pemberian obat tersebut dengan menilai kondisi penyakit. Obat lain seperti penekan imun (contohnya: azatioprin) juga dapat kami pertimbangkan untuk diberikan apabila diperlukan, dengan evaluasi oleh dokter penyakit dalam terlebih dahulu.

Bapak/Ibu/Saudara, baik yang mendapat OAT maupun yang mendapat obat metilprednosolon, akan kembali diambil sampel darahnya pada minggu ke-2 dan bulan ke-6 setelah pengobatan dimulai, untuk mengetahui perubahan pola penanda peradangan dalam darah Bapak/Ibu/Saudara mulai dari sebelum hingga selesai pengobatan.

14. Alasan memilih Bapak/Ibu/Saudara

Bapak/Ibu/Saudara kami undang sebagai subjek penelitian ini karena Bapak/Ibu/Saudara merupakan pasien RSCM Kirana yang baru terdiagnosis dengan uveitis idiopatik (peradangan pada mata yang belum diketahui penyebabnya) disertai dengan hasil IGRA yang positif (diartikan pernah terpapar dengan kuman tuberkulosis), berusia di atas 18 tahun, dan berdomisili di area Jabodetabek.

15. Prosedur penelitian

4a. Prosedur intervensi

6) Bapak/Ibu/Saudara akan menjalani prosedur pengambilan cairan bola mata yang dilakukan di ruang operasi untuk mencari tahu agen penyebab peradangan pada mata Bapak/Ibu/Saudara dengan langkah-langkah sebagai berikut:
   i. Bapak/Ibu/Saudara dalam posisi berbaring telentang
   ii. Dilakukan tindakan sterilisasi menggunakan betadine pada area mata yang sakit lalu area sekitar mata tersebut ditutup kain steril
   iii. Diberikan anestesi (obat bius) dalam bentuk tetes mata pada mata yang sakit, lalu setelah beberapa saat kelopak mata atas dan bawah diberi penyangga agar tidak mengganggu selama dilakukan tindakan
   iv. Dokter melakukan pengambilan cairan bola mata sebanyak 0.1-0.2 cc menggunakan jarum suntik berukuran 1 cc

v. Jarum ditarik dari mata lalu luka ditutup sementara dengan *cotton bud* yang telah diberi betadine

7) Apabila pada pemeriksaan cairan bola mata tidak ditemukan agen penyebab peradangan pada mata Bapak/Ibu/Saudara, maka Bapak/Ibu/Saudara termasuk dalam kategori uveitis idiopatik (peradangan pada mata yang tidak diketahui penyebabnya), sehingga langkah selanjutnya adalah pemeriksaan IGRA (pemeriksaan yang menilai adanya paparan dengan kuman tuberkulosis) dengan langkah sebagai berikut:

i. Bapak/Ibu/Saudara dalam posisi duduk senyaman mungkin, dengan lengan yang direntangkan dan bersandar pada permukaan yang rata.

ii. Petugas mencuci tangan terlebih dahulu menggunakan air dan sabun atau cairan alkohol, lalu mengenakan sarung tangan.

iii. Petugas memasangkan alat pengikat 10 cm di atas area siku (area pengambilan darah) lalu Bapak/Ibu/Saudara diminta untuk mengepalkan tangan guna memperbesar/memperjelas pembuluh darah.

iv. Petugas mengusapkan alkohol usap 70% pada area pengambilan darah lalu ditunggu hingga kering.

v. Petugas menusukkan jarum ke pembuluh darah balik di siku lalu melakukan pengambilan darah sebanyak 5 ml.

8) Setelah 1-2 minggu kemudian, bila hasil IGRA Bapak/Ibu/Saudara positif, Bapak/Ibu/Saudara akan kembali diambil sampel darahnya oleh petugas yang sudah terbiasa mengambil darah untuk mengetahui penanda peradangan pada tubuh dengan langkah-langkah sebagai berikut:

i. Bapak/Ibu/Saudara dalam posisi duduk senyaman mungkin, dengan lengan yang direntangkan dan bersandar pada permukaan yang rata.

ii. Petugas mencuci tangan terlebih dahulu menggunakan air dan sabun atau cairan alkohol, lalu mengenakan sarung tangan.

iii. Petugas memasangkan alat pengikat 10 cm di atas area siku (area pengambilan darah) lalu Bapak/Ibu/Saudara diminta untuk mengepalkan tangan guna memperbesar/memperjelas pembuluh darah.

iv. Petugas mengusapkan alkohol usap 70% pada area pengambilan darah lalu ditunggu hingga kering.

v. Petugas menusukkan jarum ke pembuluh darah balik di siku lalu melakukan pengambilan darah sebanyak 20 ml.

9) Bapak/Ibu/Saudara akan secara acak dimasukan ke dalam satu dari dua kelompok yang mendapat pengobatan berbeda, namun terapi standar tetes mata anti radang tetap diberikan kepada semua peserta.

a. Pada kelompok pertama, kami akan berikan pengantar ke bagian Penyakit Dalam RSCM untuk dinilai apakah obat anti tuberkulosis (OAT) dapat diberikan, dan dosisnya dihitung sesuai dengan berat badan Bapak/Ibu/Saudara. OAT akan diberikan untuk diminum setiap hari selama 6 bulan. Bapak/Ibu/Saudara akan tetap menjalani kontrol pemeriksaan mata ke RSCM Kirana secara berkala.

b. Pada kelompok kedua, Bapak/Ibu/Saudara akan diberikan obat anti radang (metilprednosolon) yang dosisnya disesuaikan dengan berat badan Bapak/Ibu/Saudara. Pemberiannya akan dipantau dan dievaluasi secara berkala.

10) Bapak/Ibu/Saudara, baik yang mendapat terapi OAT maupun metilprednosolon, akan kembali diambil sampel darahnya pada minggu ke-2 dan bulan ke-6 setelah pengobatan dimulai. Prosedur pengambilan sampel darah sama seperti yang tertera pada poin 3 di atas.

4b. Prosedur alternatif

Tidak ada paksaan kepada Bapak/Ibu/Saudara dalam mengikuti penelitian ini. Bapak/Ibu/Saudara dapat memutuskan untuk tidak ikut serta dalam penelitian ini dan tetap akan mendapatkan terapi tetes mata antiradang dan obat steroid/penekan imun sesuai dengan standar tatalaksana uveitis idiopatik (peradangan pada mata yang belum diketahui penyebabnya) yang berlaku saat ini.

16. Risiko prosedur penelitian

Terdapat kemungkinan terjadinya infeksi pada area pengambilan darah, namun hal ini kami minimalisasi dengan menggunakan alkohol usap dan menjaga kebersihan tangan serta lingkungan ketika dilakukan prosedur pengambilan darah. Rasa nyeri dan pegal mungkin terjadi, namun umumnya hanya berlangsung selama satu hari.

Sampai saat ini, terapi uveitis dengan penyebab yang belum diketahui adalah pemberian obat anti radang tetes mata. Obat steroid/anti radang oral (metilprednisolon) hanya diberikan pada kondisi-kondisi tertentu saja apabila dinilai perlu oleh dokter mata yang merawat. Efek samping pemberian steroid oral meliputi peningkatan tekanan bola mata, gangguan metabolisme gula darah, hingga menurunnya sistem imun tubuh apabila dikonsumsi dalam jangka waktu lama. Namun dalam penelitian ini dosis steroid oral yang diberikan sesuai dengan dosis anjuran yang berlaku, lalu akan diturunkan secara bertahap dan tentunya tetap dalam pengawasan dokter. Bapak/Ibu/Saudara akan mendapatkan jadwal kontrol rutin satu bulan sekali untuk pemantauan respon pengobatan serta efek samping obat yang mungkin terjadi, seperti pemeriksaan berat badan, tekanan darah, gula darah, dan tekanan bola mata di samping pemeriksaan rutin pada organ mata itu sendiri. Apabila terjadi efek samping dari penggunaan obat steroid, maka dosis obat dapat disesuaikan kembali atau bahkan dihentikan sama sekali. Dengan demikian, pemberian steroid oral ini dapat dikatakan relatif aman.

Sedangkan obat anti tuberkulosis (OAT) hingga saat ini belum menjadi terapi standar pada uveitis yang belum diketahui penyebabnya. Efek samping dari pemberian OAT antara lain mual, gangguan penglihatan, gangguan pendengaran, gangguan fungsi hati, kesemutan, hingga reaksi alergi. Namun semua efek samping ini akan berusaha kami cegah sejak awal dengan cara menyesuaikan dosis dengan berat badan Bapak/Ibu/Saudara. Selain itu Bapak/Ibu/Saudara akan mendapat jadwal kontrol rutin ke Departemen Penyakit Dalam yaitu pada akhir bulan ke 2, 5, dan 6 untuk memantau apabila terdapat efek samping dari penggunaan OAT. Apabila terjadi efek samping yang tidak diinginkan maka rejimen OAT dapat disesuaikan kembali atau bahkan dihentikan sama sekali. Dengan demikian, pemberian OAT ini juga dapat dikatakan relatif aman. Apabila dalam perjalanan pengobatan terjadi komplikasi atau kejadian yang tidak diharapkan, Bapak/Ibu/Saudara akan mendapat penanganan secara menyeluruh sesuai dengan prosedur yang berlaku di RSCM. Pada kondisi reaksi obat berat, pemberian OAT dapat dihentikan sewaktu-waktu.

17. Manfaat

Bapak/Ibu/Saudara akan mendapatkan terapi OAT atau steroid anti radang oral untuk penyakit uveitis yang saat ini sedang Bapak/Ibu/Saudara alami. Selain itu terkait dengan kondisi pandemi yang saat ini sedang berlangsung, diberlakukan pembatasan jumlah pasien untuk kontrol ke Poliklinik RSCM, namun dengan berpartisipasi dalam penelitian ini Bapak/Ibu/Saudara akan mendapatkan kuota tambahan pada setiap kesempatan kontrol ke Poliklinik Mata RSCM. Bapak/Ibu/Saudara tidak akan dipungut biaya apapun terkait pemeriksaan/terapi yang menjadi ranah dalam penelitian ini.

18. Kompensasi

Bapak/Ibu/Saudara akan mendapatkan uang ganti transportasi sebesar Rp 75.000 per 1 kali kedatangan kontrol lanjutan yang diperlukan untuk pemeriksaan terkait penelitian.

19. Pembiayaan

Untuk diketahui Bapak/Ibu/Saudara, biaya pemeriksaan laboratorium darah yang menjadi ranah dalam penelitian ini akan ditanggung sepenuhnya oleh peneliti, sedangkan pembiayaan untuk obat-obatan ditanggung oleh BPJS.

20. Kerahasiaan

Semua data dalam rekam medis Bapak/lbu/Saudara akan kami rahasiakan. Selain itu, kami juga meminta kesediaan Bapak/Ibu/Saudara agar darah yang telah diambil dapat kami simpan dalam waktu yang panjang pada penyimpanan khusus yang bertujuan untuk analisis/penelitian di kemudian hari.

21. Kewajiban subyek penelitian

Sebagai subyek penelitian, Bapak/Ibu/Saudara berkewajiban mengikuti aturan atau petunjuk penelitian seperti yang tertulis di atas. Bila ada yang belum jelas, Bapak/Ibu/Saudara bisa bertanya lebih lanjut kepada tim peneliti.

22. Hak untuk menolak/mengundurkan diri

Bapak/lbu/Saudara berhak untuk menolak atau mengundurkan diri dari penelitian ini apabila merasakan sesuatu yang dianggap merugikan. Jika Bapak/Ibu/Saudara menolak untuk berpartisipasi atau mundur dari penelitian ini, keputusan tersebut tidak akan mempengaruhi hubungan Bapak/Ibu/Saudara dengan saya dan tidak akan berdampak pada pelayanan yang berlaku di rumah sakit ini. Bila terjadi hal-hal yang tidak diinginkan atau keluhan lain, Bapak/lbu boleh datang kontrol kapan saja.

Untuk informasi lebih lanjut, silakan hubungi:

dr. Rina La Distia Nora, Sp.M (K), PhD
Departemen Ilmu Kesehatan Mata Fakultas Kedokteran Universitas Indonesia
Rumah Sakit Cipto Mangunkusumo, Jakarta
Telp: 0811-198-910

LEMBAR PERSETUJUAN KEIKUTSERTAAN DALAM PENELITIAN

Semua penjelasan tersebut telah disampaikan kepada saya dan semua pertanyaan saya telah dijawab oleh dr. Rina La Distia Nora, SpM(K), PhD dan tim**.** Saya mengerti bahwa bila memerlukan penjelasan, saya dapat menanyakan kepada peneliti.

<table>
<tr><th colspan="2">Sertifikat Persetujuan (Consent)</th></tr>
<tr>
<td>Saya telah membaca semua penjelasan tentang penelitian ini. Saya telah diberikan kesempatan untuk bertanya dan semua pertanyaan saya telah dijawab dengan jelas. Saya bersedia untuk berpartisipasi pada studi penelitian ini dengan sukarela.

____
Nama subjek/wali

____
Tanda tangan peserta studi

Tanggal__________________________
hari/bulan/tahun</td>
<td>Saya mengkonfirmasi bahwa peserta telah diberikan kesempatan untuk bertanya mengenai penelitian ini, dan semua pertanyaan telah dijawab dengan benar. Saya mengkonfirmasi bahwa persetujuan telah diberikan dengan sukarela.

____
Nama peneliti/peminta persetujuan

____
Tanda tangan peneliti/peminta persetujuan

Tanggal__________________________
hari/bulan/tahun</td>
</tr>
</table>

Informasi Peneliti:

Peneliti utama : dr. Rina La Distia Nora, SpM(K), PhD
Departemen Ilmu Kesehatan Mata FKUI- RSCM
Telp: 0811-198-910

Apabila subjek tuna aksara*:*

Saya telah menyaksikan pembacaan dari lembar persetujuan (*consent*) kepada subjek/partisipan penelitian dengan akurat, dan telah diberikan kesempatan untuk mengajukan pertanyaan. Saya mengkonfirmasi bahwa subjek/partisipan telah memberikan persetujuannya dengan bebas.

Nama saksi ______________________________ DAN Sidik jari subjek penelitian

Tanda tangan saksi ______________________

Tanggal ______________________________

tanggal/bulan/tahun

Attachment 2. Standardized uveitis workup procedure

**Initial step**<br>(Routine)	**Subsequent steps**<br>These consist of workups that were not performed during the initial step and may be selected based on the anatomical subtype of uveitis or combined with the first step if clinical clues are present. A multidisciplinary approach is warranted when systemic involvement is suspected. The third step is performed if no positive findings are obtained from the second step or if the results are inconclusive.		
**1st step**	**Uveitis anatomical subtype/group**	**2nd step**	**3rd step**
Routine systemic investigations:<br>12. Complete blood counts<br>13. Erythrocyte sedimentation rate or C-reactive protein<br>14. Tuberculin skin test (TST) or interferon gamma-release assay (IGRA)<br>15. Syphilis serological tests (VDRL/RPR, TPHA)<br>16. Chest X-ray<br>17. Liver and renal functions<br>18. HIV screening<br>19. T helper lymphocytes (CD4+ CD8+), if immunodeficiency is suspected or the presence of clinical presentations of cytomegalovirus retinitis<br><br>Additional investigations, if required:<br>20. Ocular fluid (aqueous tap) from the initial visit if infectious uveitis is suspected, as follows:<br>• Unilateral presentation<br>• Granulomatous inflammation<br>• Anterior uveitis with iris atrophy or increased intraocular pressure (IOP)<br>• Immunocompromised patients<br>• The presence of retinal focal lesion<br>• The presence of extensive retinitis<br>• No uveitis improvement	**Acute anterior uveitis**	• HLA-B27<br>• IGRA, if not performed	• Aqueous fluid analysis if HLA-B27 is negative<br>• Complete urine workups if there is a history of conjunctivitis with suspicion of reactive arthritis<br>• If TINU (tubulointerstitial nephritis & uveitis) is suspected, beta-2 microglobulin from urine sample<br>• β-2 microglobulin in urine sample and Angiotensin converting enzyme (ACE) for pediatric patients, adolescents, and young adults
	**Chronic anterior uveitis**	• Angiotensin Converting Enzyme (ACE)<br>• Chest CT<br>• IGRA, if not performed	• HLA-B27<br>• Serology anti PGL1 for leprae suspicion
	**Chronic granulomatous uveitis or multifocal choroiditis**	• Angiotensin Converting Enzyme (ACE)<br>• Chest CT<br>• IGRA, if not performed<br>• Toxocariasis serology<br>• Anti Toxoplasma IgG, IgM from peripheral blood and/or PCR from ocular fluid sample	If there are clinical indications only:<br>• Minor salivary gland biopsy<br>• Bronchoscopy dan bronchoalveolar lavage<br>• 18-FDG PET or 67Ga scintigraphy<br>• Cryptococcus serology<br>• Serology anti PGL1
	**Chronic intermediate uveitis**		• Lumbal puncture<br>• Brain MRI
	**Chronic posterior uveitis**	• Anti Toxoplasma IgG, IgM from peripheral blood and/or PCR from ocular fluid.<br>• Angiotensin converting enzyme (ACE)<br>• Chest CT<br>• IGRA, if not performed<br>• ANCA (if there is a	• Lumbal puncture for cytology<br>• Brain MRI

with previous empiric/standard treatment.<br><br>21. Diagnostic vitrectomy may be performed if malignancy is suspected<br>22. The 2nd step can be directly considered if there is a suspicion of a relevant clinical presentations.		suspicion of Wegener's granulomatosis (WG) or granulomatosis with polyangitis (GPA)) [21, 2221, 2221, 2221, 2221, 22]<br>• If clinically suspected: fungal investigations from ocular fluid	
	**Panuveitis**	• Anti Toxoplasma IgG, IgM from peripheral blood and/or PCR from ocular fluid.<br>• Angiotensin converting enzyme (ACE)<br>• Chest CT<br>• IGRA, if not performed	• Lumbal puncture<br>• Brain MRI<br>• HLA-B27
	**Isolated retinal vasculitis**	• IGRA, if not performed<br>• Complement factors<br>• Antinuclear antibody (ANA)<br>• Antiphospholipid antibody<br>• Anti cytoplasmic antineutrophil antibody (ANCA)	• Angiotensin converting enzyme (ACE)<br>• Complete urine workups
	**Severe uveitis recalcitrant to immunosuppr essants**	• IGRA, if not performed<br>• Ocular fluid (aqueous tap/vitrectomy): for malignancy investigations or infection	• Re-vitrectomy (if malignancy is suspected)<br>• Brain MRI<br>• Angiotensin converting enzyme (ACE)
	**Scleritis**	• IGRA<br>• ANCA<br>• Rheumatoid factor /anti-CCP<br>• ANA	• Complete urine workups<br>• HLA-B27<br>• Angiotensin converting enzyme (ACE)<br>• Anti HCV<br>• HbsAg<br>• C3, C4
	**Paediatric cases**	• IGRA, if not performed<br>• ANA<br>• If immunodeficiency is suspected (IgG, IgM) anti Toxoplasma, CMV, HSV. Ocular fluid analysis if indicated.	• Complete urine workups<br>• HLA-B27

Summary of protocol changes

<table>
<tr><th>Protocol version</th><th>Date</th><th>Summary of changes</th></tr>
<tr><td>Version 1.0</td><td>20 May 2021</td><td rowspan="2">a. Duration of antitubercular treatment: from 6 months (2RHZE/4RH) to 9 months (2RHZE/7RH)
b. End of study: from December 2022 to February 2024. This prediction is based on the outpatient clinic patient volume during the COVID-19 pandemic
c. Addition of ocular-level outcomes: visual acuity and secondary glaucoma
d. Statistical analysis for potential switching</td></tr>
<tr><td>Version 2.0</td><td>21 June 2021</td></tr>
</table>

STATISTICAL ANALYSIS PLAN

Study title: The Effectivity of Anti Tuberculosis Therapy in Idiopathic Uveitis With Positive Interferon Gamma Release Assay (IGRA): A Randomized Clinical Trial (version 1.0)

1. **Main study objective**

   The primary objective of the study is to determine whether antitubercular treatment (ATT) would eventually improve outcomes compared to treatment without ATT in patients with uveitis of undetermined cause with positive Interferon Gamma Release Assay (IGRA).

2. **Other (secondary) study objective(s)**
   - The details of treatment failure (partial resolution or non-responsive)
   - Time to uveitis resolution
   - Uveitis relapse following complete resolution,

3. **Study design**

   Single-blinded, randomized controlled trial. This study will be conducted at Cipto Mangunkusumo Hospital, a tertiary eye hospital in Jakarta, Indonesia.

Treatment arms

Study arms	Intervention	Control
**Anti-Tuberculosis Therapy**	Yes	No
	Dosage form: ATT fixed-dose combination (FDC). FDC intensive phase containing 150 mg rifampicin, 75 mg isoniazid, 400 mg pyrazinamide, and 275 mg ethambutol), while FDC continuation phase containing rifampicin-isoniazid. Dosage: according to body weight, 30-37 kg: 2 tablets, 38-54 kg: 3 tablets, 55-70 kg: 4 tablets, more than 70 kg: 5 tablets. Frequency: Intensive phase: once daily. Continuation phase: 3 times/week. Duration: ≥ 6 months* (2 months of	-

	FDC intensive phase + ≥4 months of FDC continuation phase). Treatment duration will be determined by the pulmonologist according to local guidelines. Once decided, the duration will be standardized for all included participants, with exceptions for those requiring ATT adjustments due to other factors (e.g., adverse effects, allergies, etc.).	
**Immunosuppressants**	Possible (Depending on the clinical presentation and the clinical judgment of the blinded attending uveitis specialist)	Yes
	Oral methylprednisolone at a dosage of 0.8 mg/kg of body weight per day (maximum of 56 mg/day), which will be tapered gradually based on the intraocular inflammation observed. This dose is equivalent to 1 mg/kgBW/day (max 60 mg/day).<br>Tapering of oral methylprednisolone involve reducing the dose by 8 mg for doses above 20 mg and by 4 mg for doses below 20 mg.<br>All patients receiving systemic steroids, including those in the ATT group, will also receive vitamin D, calcium supplements, and gastric protectors (e.g., omeprazole or antacids).<br>Local steroids (e.g., steroid drops) will be prescribed based on the clinical presentation.<br>Disease-modifying anti-rheumatic drugs (DMARDs) may be prescribed based on the clinical judgment of the attending pulmonologist/internist-immunologist.	

4. **Main study parameter (endpoint)**

<u>Response to treatment</u>

g. At 6 month follow-up:
   Criteria for complete uveitis resolution applied to both eyes, in cases with bilateral uveitis, included[18]: (1) fewer than or equal to 0.5+ anterior chamber cells according to the Standardized Uveitis Nomenclature (SUN) grading system, fewer than or equal to 0.5+

vitreous haze based on clinical grading using the NEI (National Eye Institute) scale, and no active retinal or choroidal lesions; and (2) no more than 7.5 mg of oral prednisone daily (i.e., methylprednisolone at 6 mg or less daily) and fewer than or equal to 2 drops of prednisolone acetate 1% (or equivalent) per day. Treatment will be categorized as a failure if subject did not meet the following criteria at the 6-month follow-up.

Type of data: Categorical

5. Additional study parameters (endpoints)

a. Time to uveitis resolution: Calculated from the time of randomization.
Type of data: time (in months)

b. Uveitis relapse (subsequent follow-up, beyond 6 months)
Relapse: any worsening of ocular inflammation (including a two-step increase in anterior chamber or vitreous cells as per the SUN grading system) or the occurrence of clinically new inflammatory activity (such as choroidal or retinal lesions) that necessitated a modification of local or systemic uveitis treatment after a minimum of 90 days of complete uveitis resolution at the patient level[19, 20].
Type of data: Categorical

c. Time to relapse was calculated from the first visit showing inactive uveitis until the first notification of uveitis relapse.
Type of data: time (in months)

6. Other baseline variables

i. Age (numerical data)
ii. Laterality of ocular inflammation (categorical data): unilateral or bilateral
iii. Sex (categorical data): male or female
iv. Onset (numerical data): days of ocular complaints
v. Chronicity (categorical data): acute or chronic
vi. Previous TB (categorical data): yes or no
vii. Contact TB history (categorical data): yes or no
viii. Baseline visual acuity (numerical data): Snellen/LogMAR visual acuity, and categorical data: mild visual impairment, moderate visual impairment, severe visual impairment, and blindness.

ix. IOP (numerical and categorical data): increased or not
x. Keratic precipitates (categorical data): mutton-fat, fine, or others
xi. Anterior chamber cells (numerical data)
xii. The presence of iris nodule (categorical data): yes or no
xiii. Scleral involvement (categorical data): yes or no
xiv. Synechia formation (categorical data): yes or no
xv. Lens status (categorical data): cataract, pseudophakia, phakic)
xvi. Vitreous haziness (categorical data): yes or no
xvii. Vitreous cells (numerical data)
xviii. Snowball/snowbank (categorical data): yes or no
xix. Retinal vascular changes (categorical data): yes or no
xx. Optic nerve involvement (categorical data): yes or no
xxi. Retinitis or retinal infiltrates (categorical data): yes or no
xxii. Choroiditis (categorical data): yes or no
xxiii. Posterior granuloma/tuberculoma appearance (categorical data): yes or no
xxiv. Retinal detachment (categorical data): yes or no
xxv. Macular edema (categorical data): yes or no

7. **Sample size calculation**

The sample size is calculated using a formula of two-independent proportion sample size calculation. The primary end point of uveitis resolution at 6 month is used for this sample size calculation.

Based on the Multicenter Uveitis Steroid Treatment (MUST) trial in non-infectious uveitis, the proportion of uveitis patients with active disease at six months following treatment was found to be 44%[17]. A 30% difference in uveitis resolution between the two groups is predetermined. Using a $Z\alpha$ of 1.96 and a $Z\beta$ of 0.84 (for a two-sided significance level of 5% and 80% power), the estimated sample size required for each treatment group will be 35 participants. With 10% increase to anticipate dropout, a total of 39-40 participants in each treatment group is required.

$$N1 = N2 = \left(\frac{Z\alpha\sqrt{2PQ}+Z\beta\sqrt{P1Q1+P2Q2}}{P1-P2}\right)^2$$

$Z\alpha$ (1.96), power (80%, $Z\beta = 0.84$), P1 based on MUST trial 44% (Q2 = 56%). Estimated P1 – P2 = 30%. Thus, P2 = 14% (Q2 = 86%). As 10% drop out rate is being anticipated, N1 = N2 = 39 ~ 40 (total 78-80 participants)

8. Randomization

After eligibility assessment, participants will be randomly assigned in a 1:1 ratio to one of the two treatment groups without stratification factors. Randomization will be performed by a dedicated research manager/assistant using block randomization (block size of 4) to ensure equal distribution between the groups.

(Note: Patients presenting with choroidal granuloma or serpiginous-like choroiditis will be directly assigned to the ATT group, in accordance with recently published guidelines coinciding with the start of this trial[13]. Adjustments to the randomization will be made afterwards to maintain the block size)

9. Study analysis population

a. The intention-to-treat (ITT) approach

All analyses will be first conducted using the ITT approach. The ITT population will include all participants who underwent randomization, regardless of any treatment switching that occurred. For participants who missed the six-month visit (±28 days), if any, last observation carried forward (LOCF) imputation will be performed..

b. The per-protocol approach

The per-protocol population will include all participants who were randomized, excluding those who do not have six-month visits or deviated from the treatment protocols (e.g., non-compliant, experience severe adverse effect, etc). If any, participants who switch treatment will also be excluded in this per-protocol analysis.

For patients receiving ATT, missing 2 weeks of medication will be considered non-compliance (protocol deviation). The pulmonologist will record the prescribed ATT and count the remaining drugs (i.e., ATT) at each visit to determine treatment non-compliance. For participants receiving systemic immunosuppressants, missing more than 1 week of the prescribed regimen will be considered non-compliance. Topical immunosuppressants will not be taken into account when determining compliance.

10. Study follow-up

Participants will be assessed at screening, baseline (Week 0), Week 2, and then at monthly intervals until the primary endpoint at 6 months (Month 1, Month 2, Month 3, Month 4, Month 5, and Month 6). If deemed necessary by the attending uveitis specialist or pulmonologist, visits outside this schedule will be possible while preserving blinding. The research manager will continue contacting participants for subsequent monthly visits beyond the primary endpoint at 6 months, though these visits are not mandatory. If a scheduled visit does not occur or if its results are missing, the value measured on the date closest to the target scheduled visit within a window period of ±2 weeks will be used. If the latest visit does not fall within the 6-month period (±2 weeks), the closest visit to this timeframe will be used.

11. Safety

Adverse events will be recorded from all enrolled participants who had received at least one dose of study treatment (exposed) of any drug. Safety analyses will be based on the treatment regimen.

The safety analysis will be based on reported adverse events (AEs) in the case report forms, as well as any other identifiable information from each clinic visit, including those at the uveitis clinic or internal medicine/pulmonology clinic. This will also include data from medical records or laboratory values for each participant during the trial period.

12. Interim analysis

Interim analysis, if necessary, may be performed by an external statistician if recommended by the local ethical committee. The ethical committee will oversee trial monitoring, which may occur at any time during the trial duration.

13. Reporting exploratory biomarkers

As part of this study, samples derived from all enrolled participants will be stored in the local biobank. Any measurement or analysis used to describe the biological process and its relation to treatment derived from this trial will be linked to the actual treatment received.

14. Data analysis

a. All data will be pseudo-anonymized. A separate dataset (in Excel) containing participant IDs will be protected. A separate dataset without direct participant IDs will be generated and analyzed using SPSS.

b. Baseline characteristics will be summarized according to trial group for the ITT populations.

c. The primary outcome analysis will be focused on the proportion of complete uveitis resolution and the corresponding relative risk between the ATT and control groups. Cox proportional-hazards models will be used to estimate any uveitis resolution between the ATT and control groups. This analysis will be performed at the patient level, where resolution of uveitis is considered when both eyes meet the prespecified criteria. In bilateral uveitis cases, if one eye shows uveitis resolution but the other does not, as prespecified in the previous section, these participants will be considered to have "no uveitis resolution".

d. Pairwise comparisons across experimental arms will be done using a Chi-square test or Fisher's exact test as appropriate at the 5% significance level for the efficacy endpoint of uveitis resolution at six month time point (see above section). Relative risk will be calculated and presented with 95% confidence interval in the ITT and per-protocol analysis approaches.

e. Time-to-uveitis resolution will be analyzed using the Cox proportional hazards model and hazard ratio.

f. For uveitis relapse outcomes, the analysis will include participants with complete uveitis resolution, regardless of follow-up duration. Cox proportional hazards models, along with hazard ratio and Kaplan–Meier estimates, will be used for the analysis.

g. The number (proportion) of participants experiencing any adverse events will be reported.

h. A p-value of less than 0.05 will be considered statistically significant.

i. All statistical analyses will be performed using IBM SPSS Statistics version 28.0.1.0 (142) for Windows (SPSS Inc.).

15. References

1. Rathinam SR, Gonzales JA, Thundikandy R, Kanakath A, Murugan SB, Vedhanayaki R *et al.* Effect of Corticosteroid-Sparing Treatment With Mycophenolate Mofetil vs Methotrexate on Inflammation in Patients With Uveitis: A Randomized Clinical Trial. *Jama* 2019; **322**(10)**:** 936-945.

2. Jabs DA, Nussenblatt RB, Rosenbaum JT, Standardization of Uveitis Nomenclature Working G. Standardization of uveitis nomenclature for reporting clinical data. Results of the First International Workshop. *Am J Ophthalmol* 2005; **140**(3)**:** 509-516.

3. Agrawal R, Agarwal A, Jabs DA, Kee A, Testi I, Mahajan S *et al.* Standardization of Nomenclature for Ocular Tuberculosis - Results of Collaborative Ocular Tuberculosis Study (COTS) Workshop. *Ocul Immunol Inflamm* 2020; **28**(sup1)**:** 74-84.

4. Multicenter Uveitis Steroid Treatment Trial Research G, Kempen JH, Altaweel MM, Holbrook JT, Jabs DA, Louis TA *et al.* Randomized comparison of systemic anti-inflammatory therapy versus fluocinolone acetonide implant for intermediate, posterior, and panuveitis: the multicenter uveitis steroid treatment trial. *Ophthalmology* 2011; **118**(10)**:** 1916-1926.

5. Agrawal R, Testi I, Bodaghi B, Barisani-Asenbauer T, McCluskey P, Agarwal A *et al.* Collaborative Ocular Tuberculosis Study Consensus Guidelines on the Management of Tubercular Uveitis-Report 2: Guidelines for Initiating Antitubercular Therapy in Anterior Uveitis, Intermediate Uveitis, Panuveitis, and Retinal Vasculitis. *Ophthalmology* 2021; **128**(2)**:** 277-287.

STATISTICAL ANALYSIS PLAN

Study title: The Effectivity of Anti Tuberculosis Therapy in Idiopathic Uveitis With Positive Interferon Gamma Release Assay (IGRA): A Randomized Clinical Trial (version 2.0)

1. **Main study objective**
   The primary objective of the study is to determine whether antitubercular treatment (ATT) would eventually improve outcomes compared to treatment without ATT in patients with uveitis of undetermined cause with positive Interferon Gamma Release Assay (IGRA).

2. **Other (secondary) study objective(s)**
   - The details of treatment failure (partial resolution or non-responsive)
   - Time to uveitis resolution
   - Uveitis relapse following complete resolution
   - Ocular-level outcomes: visual acuity changes and secondary glaucoma

3. **Study design**
   Single-blinded, randomized controlled trial. This study will be conducted at Cipto Mangunkusumo Hospital, a tertiary eye hospital in Jakarta, Indonesia.

Treatment arms

**Study arms**	**Intervention**	**Control**
**Anti-Tuberculosis Therapy**	Yes	No
	Dosage form: ATT fixed-dose combination (FDC). FDC intensive phase containing 150 mg rifampicin, 75 mg isoniazid, 400 mg pyrazinamide, and 275 mg ethambutol), while FDC continuation phase containing rifampicin-isoniazid. Dosage: according to body weight, 30-37 kg: 2 tablets, 38-54 kg: 3 tablets, 55-70 kg: 4 tablets, more than 70 kg: 5 tablets. Frequency: Intensive phase: once daily.	-

	Continuation phase: 3 times/week. Duration: 9 months (2 months of FDC intensive phase + 7 months of FDC continuation phase). The duration will be standardized for all included participants, with exceptions for those requiring ATT adjustments due to other factors (e.g., adverse effects, allergies, etc.).	
**Immunosuppressants**	Possible (Depending on the clinical presentation and the clinical judgment of the blinded attending uveitis specialist)	Yes
	Oral methylprednisolone at a dosage of 0.8 mg/kg of body weight per day (maximum of 56 mg/day), which will be tapered gradually based on the intraocular inflammation observed. This dose is equivalent to 1 mg/kgBW/day (max 60 mg/day).<br>Tapering of oral methylprednisolone involve reducing the dose by 8 mg for doses above 20 mg and by 4 mg for doses below 20 mg. All patients receiving systemic steroids, including those in the ATT group, will also receive vitamin D, calcium supplements, and gastric protectors (e.g., omeprazole or antacids).<br>Local steroids (e.g., steroid drops) will be prescribed based on the clinical presentation.<br>Disease-modifying anti-rheumatic drugs (DMARDs) may be prescribed based on the clinical judgment of the attending pulmonologist/internist-immunologist.	

4. **Main study parameter (endpoint)**

<u>Response to treatment</u>

a. At 6 month follow-up:
   Criteria for complete uveitis resolution applied to both eyes, in cases with bilateral uveitis, included[18]: (1) fewer than or equal to 0.5+ anterior chamber cells according to the Standardized Uveitis Nomenclature (SUN) grading system, fewer than or equal to 0.5+ vitreous haze based on clinical grading using the NEI (National Eye Institute) scale, and no

active retinal or choroidal lesions; and (2) no more than 7.5 mg of oral prednisone daily (i.e., methylprednisolone at 6 mg or less daily) and fewer than or equal to 2 drops of prednisolone acetate 1% (or equivalent) per day. Treatment will be categorized as a failure if subject did not meet the following criteria at the 6-month follow-up.

Type of data: Categorical

5. Additional study parameters (endpoints)

a. Time to uveitis resolution: Calculated from the time of randomization.
Type of data: time (in months)

b. Uveitis relapse (subsequent follow-up, beyond 6 months)
Relapse: any worsening of ocular inflammation (including a two-step increase in anterior chamber or vitreous cells as per the SUN grading system) or the occurrence of clinically new inflammatory activity (such as choroidal or retinal lesions) that necessitated a modification of local or systemic uveitis treatment after a minimum of 90 days of complete uveitis resolution at the patient level[19, 20].
Type of data: Categorical

c. Time to relapse was calculated from the first visit showing inactive uveitis until the first notification of uveitis relapse.
Type of data: time (in months)

Ocular-level outcomes

d. Secondary glaucoma: the occurrence of increased intraocular pressure (IOP) above 21 mmHg in at least two consecutive visits that necessitated prescription of IOP-lowering medications.
Type of data: Categorical

e. Visual acuity: Visual acuity was considered to increase if the difference in best-corrected visual acuity between month 6 and baseline visits (measured with Snellen, in decimal equivalent) was equal to or greater than 0.1, stable if within the range of -0.1 to 0.1, and decreased if less than -0.1.
(If any cataract or vitreoretinal surgery was performed before the six-month follow-up, the last recorded visual acuity prior to the surgery was used).

Type of data: Categorical

6. Other baseline variables

xxvi. Age (numerical data)

xxvii. Laterality of ocular inflammation (categorical data): unilateral or bilateral

xxviii. Sex (categorical data): male or female

xxix. Onset (numerical data): days of ocular complaints

xxx. Chronicity (categorical data): acute or chronic

xxxi. Previous TB (categorical data): yes or no

xxxii. Contact TB history (categorical data): yes or no

xxxiii. Baseline visual acuity (numerical data): Snellen/LogMAR visual acuity, and categorical data: mild visual impairment, moderate visual impairment, severe visual impairment, and blindness.

xxxiv. IOP (numerical and categorical data): increased or not

xxxv. Keratic precipitates (categorical data): mutton-fat, fine, or others

xxxvi. Anterior chamber cells (numerical data)

xxxvii. The presence of iris nodule (categorical data): yes or no

xxxviii. Scleral involvement (categorical data): yes or no

xxxix. Synechia formation (categorical data): yes or no

xl. Lens status (categorical data): cataract, pseudophakia, phakic)

xli. Vitreous haziness (categorical data): yes or no

xlii. Vitreous cells (numerical data)

xliii. Snowball/snowbank (categorical data): yes or no

xliv. Retinal vascular changes (categorical data): yes or no

xlv. Optic nerve involvement (categorical data): yes or no

xlvi. Retinitis or retinal infiltrates (categorical data): yes or no

xlvii. Choroiditis (categorical data): yes or no

xlviii. Posterior granuloma/tuberculoma appearance (categorical data): yes or no

xlix. Retinal detachment (categorical data): yes or no

l. Macular edema (categorical data): yes or no

7. Sample size calculation

The sample size is calculated using a formula of two-independent proportion sample size calculation. The primary end point of uveitis resolution at 6 month is used for this sample size calculation.

Based on the Multicenter Uveitis Steroid Treatment (MUST) trial in non-infectious uveitis, the proportion of uveitis patients with active disease at six months following treatment was found to be 44%[17]. A 30% difference in uveitis resolution between the two groups is predetermined. Using a Zα of 1.96 and a Zβ of 0.84 (for a two-sided significance level of 5% and 80% power), the estimated sample size required for each treatment group will be 35 participants. With 10% increase to anticipate dropout, a total of 39-40 participants in each treatment group is required.

$$N1 = N2 = \left(\frac{Z\alpha\sqrt{2PQ}+Z\beta\,\sqrt{P1Q1+P2Q2}}{P1-P2}\right)^2$$

Zα (1.96), power (80%, Zβ = 0.84), P1 based on MUST trial 44% (Q2 = 56%). Estimated P1 – P2 = 30%. Thus, P2 = 14% (Q2 = 86%). As 10% drop out rate is being anticipated, N1 = N2 = 39 ~ 40 (total 78-80 participants)

8. Randomization

After eligibility assessment, participants will be randomly assigned in a 1:1 ratio to one of the two treatment groups without stratification factors. Randomization will be performed by a dedicated research manager/assistant using block randomization (block size of 4) to ensure equal distribution between the groups.

(Note: Patients presenting with choroidal granuloma or serpiginous-like choroiditis will be directly assigned to the ATT group, in accordance with recently published guidelines coinciding with the start of this trial[13]. Adjustments to the randomization will be made afterwards to maintain the block size)

9. Study analysis population

c. The intention-to-treat (ITT) approach

All analyses will be first conducted using the ITT approach. The ITT population will include all participants who underwent randomization, regardless of any treatment switching that occurred. For participants who missed the six-month visit (±28 days), if any, last observation carried forward (LOCF) imputation will be performed..

d. The per-protocol approach

The per-protocol population will include all participants who were randomized, excluding those who do not have six-month visits or deviated from the treatment protocols (e.g., non-compliant, experience severe adverse effect, etc). If any, participants who switch treatment will also be excluded in this per-protocol analysis.

For patients receiving ATT, missing 2 weeks of medication will be considered non-compliance (protocol deviation). The pulmonologist will record the prescribed ATT and count the remaining drugs (i.e., ATT) at each visit to determine treatment non-compliance. For participants receiving systemic immunosuppressants, missing more than 1 week of the prescribed regimen will be considered non-compliance. Topical immunosuppressants will not be taken into account when determining compliance.

e. Additional approach for potential switching

In order to anticipate treatment switch, adjustment will be performed, particularly to analyze the secondary outcomes. Participants grouping will be subdivided into those receiving combination treatment besides the initial group of ATT alone and non-ATT. Specifically, participants that switch from non-ATT group to ATT will be classified as ATT in this adjusted analysis. Analysis following adjustment for switching treatment was performed with recalculation of the six-month follow-up time based on the actual treatment received, beginning from the start of their actual treatment.

However, results from the ITT analysis (without adjustment for switching) is considered to be more appropriate to evaluate the primary outcome, given that the purpose of the study was to compare the two strategies according to the treatment received at the time of randomization.

10. Study follow-up

Participants will be assessed at screening, baseline (Week 0), Week 2, and then at monthly intervals until the primary endpoint at 6 months (Month 1, Month 2, Month 3, Month 4, Month 5, and Month 6). If deemed necessary by the attending uveitis specialist or pulmonologist, visits outside this schedule will be possible while preserving blinding. The research manager will continue contacting participants for subsequent monthly visits beyond the primary endpoint at 6 months, though these visits are not mandatory. If a scheduled visit does not occur or if its results are missing, the value measured on the date closest to the target scheduled visit within a

window period of ±2 weeks will be used. If the latest visit does not fall within the 6-month period (±2 weeks), the closest visit to this timeframe will be used.

11. Safety

Adverse events will be recorded from all enrolled participants who had received at least one dose of study treatment (exposed) of any drug. Safety analyses will be based on the treatment regimen.

The safety analysis will be based on reported adverse events (AEs) in the case report forms, as well as any other identifiable information from each clinic visit, including those at the uveitis clinic or internal medicine/pulmonology clinic. This will also include data from medical records or laboratory values for each participant during the trial period.

12. Interim analysis

Interim analysis, if necessary, may be performed by an external statistician if recommended by the local ethical committee. The ethical committee will oversee trial monitoring, which may occur at any time during the trial duration.

13. Reporting exploratory biomarkers

As part of this study, samples derived from all enrolled participants will be stored in the local biobank. Any measurement or analysis used to describe the biological process and its relation to treatment derived from this trial will be linked to the actual treatment received.

14. Data analysis

a. All data will be pseudo-anonymized. A separate dataset (in Excel) containing participant IDs will be protected. A separate dataset without direct participant IDs will be generated and analyzed using SPSS.
b. Baseline characteristics will be summarized according to trial group for the ITT populations.
c. The primary outcome analysis will be focused on the proportion of complete uveitis resolution and the corresponding relative risk between the ATT and control groups. Cox proportional-hazards models will be used to estimate any uveitis resolution between the ATT and control groups. This analysis will be performed at the patient level, where resolution of uveitis is considered when both eyes meet the prespecified criteria. In bilateral

uveitis cases, if one eye shows uveitis resolution but the other does not, as prespecified in the previous section, these participants will be considered to have "no uveitis resolution".

d. Pairwise comparisons across experimental arms will be done using a Chi-square test or Fisher's exact test as appropriate at the 5% significance level for the efficacy endpoint of uveitis resolution at six month time point (see above section). Relative risk will be calculated and presented with 95% confidence interval in the ITT and per-protocol analysis approaches.
e. Time-to-uveitis resolution will be analyzed using the Cox proportional hazards model and hazard ratio.
f. For uveitis relapse outcomes, the analysis will include participants with complete uveitis resolution, regardless of follow-up duration. Cox proportional hazards models, along with hazard ratio and Kaplan–Meier estimates, will be used for the analysis.
g. The number (proportion) of participants experiencing any adverse events will be reported.
h. Eye-level outcomes will be calculated based on the involved eyes for the presenting uveitis. Therefore, bilateral uveitis presentations will be counted as two eyes.
i. A *p*-value of less than 0.05 will be considered statistically significant.
j. All statistical analyses will be performed using IBM SPSS Statistics version 28.0.1.0 (142) for Windows (SPSS Inc.).

15. References

1. Rathinam SR, Gonzales JA, Thundikandy R, Kanakath A, Murugan SB, Vedhanayaki R *et al.* Effect of Corticosteroid-Sparing Treatment With Mycophenolate Mofetil vs Methotrexate on Inflammation in Patients With Uveitis: A Randomized Clinical Trial. *Jama* 2019; **322**(10)**:** 936-945.

2. Jabs DA, Nussenblatt RB, Rosenbaum JT, Standardization of Uveitis Nomenclature Working G. Standardization of uveitis nomenclature for reporting clinical data. Results of the First International Workshop. *Am J Ophthalmol* 2005; **140**(3)**:** 509-516.

3. Agrawal R, Agarwal A, Jabs DA, Kee A, Testi I, Mahajan S *et al.* Standardization of Nomenclature for Ocular Tuberculosis - Results of Collaborative Ocular Tuberculosis Study (COTS) Workshop. *Ocul Immunol Inflamm* 2020; **28**(sup1)**:** 74-84.

4. Multicenter Uveitis Steroid Treatment Trial Research G, Kempen JH, Altaweel MM, Holbrook JT, Jabs DA, Louis TA *et al.* Randomized comparison of systemic anti-inflammatory therapy versus fluocinolone acetonide implant for intermediate, posterior, and panuveitis: the multicenter uveitis steroid treatment trial. *Ophthalmology* 2011; **118**(10)**:** 1916-1926.

5. Agrawal R, Testi I, Bodaghi B, Barisani-Asenbauer T, McCluskey P, Agarwal A *et al.* Collaborative Ocular Tuberculosis Study Consensus Guidelines on the Management of Tubercular Uveitis-Report 2: Guidelines for Initiating Antitubercular Therapy in Anterior Uveitis, Intermediate Uveitis, Panuveitis, and Retinal Vasculitis. *Ophthalmology* 2021; **128**(2)**:** 277-287.

Summary of statistical analysis plan changes

<table>
<tr><th>Protocol version</th><th>Summary of changes</th></tr>
<tr><td>Version 1.0</td><td rowspan="2">a. Addition of ocular-level outcomes: visual acuity and secondary glaucoma
b. Statistical analysis for potential switching</td></tr>
<tr><td>Version 2.0</td></tr>
</table>
